# Synthetic Retinoid Sulfarotene Selectively Inhibits Tumor‐Repopulating Cells of Intrahepatic Cholangiocarcinoma via Disrupting Cytoskeleton by P‐Selectin/PSGL1 N‐Glycosylation Blockage

**DOI:** 10.1002/advs.202407519

**Published:** 2024-11-28

**Authors:** Xiaojing Du, Zhuoran Qi, Sinuo Chen, Jinlan Wu, Ye Xu, Sunkuan Hu, Zhijie Yu, Jiayun Hou, Yuan Fang, Jinglin Xia, Xin Cao

**Affiliations:** ^1^ Liver Cancer Institute Zhongshan Hospital Fudan University 180 Fenglin Road Shanghai 200032 China; ^2^ Endoscopy Center Shanghai East Hospital Tongji University School of Medicine Shanghai 200120 China; ^3^ Department of Pediatrics Jiading District Central Hospital Shanghai 201800 China; ^4^ Department of Gastroenterology The First Affiliated Hospital of Wenzhou Medical University Wenzhou 325000 China; ^5^ Key Laboratory of Diagnosis and Treatment of Severe Hepato‐Pancreatic Diseases of Zhejiang Province The First Affiliated Hospital of Wenzhou Medical University Wenzhou 325000 China; ^6^ Wenzhou Key Laboratory of Hematology The First Affiliated Hospital of Wenzhou Medical University Wenzhou 325000 China; ^7^ Biomedical Research Center Zhongshan Hospital Institute of Clinical Science Fudan University Shanghai 200032 China; ^8^ Department of Liver Surgery Key Laboratory of Carcinogenesis and Cancer Invasion (Ministry of Education) Liver Cancer Institute Zhongshan Hospital Fudan University Shanghai 200032 China; ^9^ Zhejiang Key Laboratory of Intelligent Cancer Biomarker Discovery and Translation First Affiliated Hospital of Wenzhou Medical University Wenzhou 325035 China; ^10^ Institute of Clinical Science Zhongshan Hospital Fudan University Shanghai 200032 China

**Keywords:** core fucosylation, cytoskeleton, intrahepatic cholangiocarcinoma, P‐selectin/PSGL1, synthetic retinoid, tumor‐repopulating cells

## Abstract

Intrahepatic cholangiocarcinoma (ICC) is a highly lethal malignancy that currently lacks effective clinical treatments. Eliminating stem cell‐like cancer cells is an extremely promising but challenging strategy for treating ICC. A recently developed synthetic retinoid, sulfarotene, abrogates proliferation, and induces apoptosis of tumor‐repopulating cells (TRCs) that exhibit stem cell‐like properties, yet its effect and underlying mechanisms remain elusive in ICC. It is found that although 5‐fluorouracil, cisplatin, pemigatinib, and gemcitabine all inhibit ICC‐TRCs, sulfarotene demonstrates superior efficacy. Sulfarotene induces retinoic acid receptor alpha (RARɑ) translocation from the cytoplasm to the nucleus, suppressing P‐selectin expression at the transcriptional level. Moreover, it directly interacts with fucosyltransferase 8 (FUT8), inhibiting the core fucosylation of P‐selectin glycoprotein ligand 1 (PSGL1). These actions collectively inhibit ICC‐TRCs via destroying PSGL1‐regulated cytoskeleton. The findings provide a strategy of inhibiting P‐selectin/PSGL1 interaction and altering PSGL1 glycosylation pattern to compromise the cytoskeletal integrity and eliminate ICC‐TRCs.

## Graphical abstract

Eliminating cancer stem‐like cells is a promising strategy for treating intrahepatic cholangiocarcinoma (ICC). Synthetic retinoid sulfarotene induces RARɑ translocation from the cytoplasm to the nucleus, suppressing P‐selectin expression at the transcriptional level. It also directly interacts with FUT8, inhibiting the core fucosylation of PSGL1. These actions collectively destroy PSGL1‐regulated cytoskeleton, leading to the death of cancer stem‐like cells in ICC.

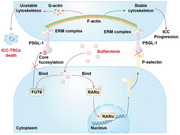

## 1 Introduction

Intrahepatic cholangiocarcinoma (ICC) ranks second among the primary liver cancer with a notably high incidence in China and Southeast Asia, accounting for up to 20% of hepatic malignancies and 3% of all digestive system malignancies.^[^ ^1^ ^]^ It is a highly aggressive and lethal disease, with a 5‐year overall survival (OS) remaining around 9%. Only 20%–30% patients are diagnosed at resectable stage, but the 5‐year OS rate is only about 20%–35% even after surgical resection.^[^ ^1^ ^]^ By comparison, the majority of patients with unresectable disease have a median OS (mOS) of 11.7 months.^[^ ^1^ ^]^ Though the rapidly evolving surgical and systemic therapeutic landscape, including IDH (ivosidenib) and FGFR2 (pemigatinib) target therapy,^[^ ^2^ ^]^ the outcomes remain poor. Therefore, it is urgent to explore more effective treatment options for ICC.

Tumor‐repopulating cells (TRCs) represent a self‐renewing and highly tumorigenic subpopulation of cancer cells that play crucial roles in the key events of cancers. These events include sustaining proliferation, resisting cell death, distant metastasis, immune evasion, metabolic reprogramming, senescence‐associated secretory phenotype, and drug resistance, etc.^[^ ^3^, ^4^, ^5^, ^6^, ^7^, ^8^, ^9^, ^10^ ^]^ Targeting TRCs, which exhibit characteristics similar to cancer stem cells (CSCs), holds promise for developing effective treatments for various cancers, including ICC. To eliminate these TRCs, our group developed a novel synthetic retinoid, termed sulfarotene (WYC‐209, SFT).^[^ ^11^ ^]^ It exerted an outstanding inhibition against TRCs of various cancer types, including hepatocellular carcinoma (HCC), melanoma, and gastric cancer, in vitro and in vivo evaluation including patient‐derived xenograft (PDX) models,^[^ ^11^, ^12^, ^13^, ^14^ ^]^ so it was served as a potential TRCs targeted molecular. The selective inhibition of SFT against TRCs may tackle the current ICC problems as aforementioned.

In this research, we demonstrated that SFT effectively suppresses TRCs of ICC (ICC‐TRCs) as well as xenograft tumors established from ICC‐TRCs. Mechanically, SFT promoted the traffic of retinoic acid receptor alpha (RARɑ) from cytoplasm to nucleus, thereby inhibiting the transcription of SELP and decreasing the expression of P‐selectin. In addition, SFT directly binds to fucosyltransferase 8 (FUT8), impairing its enzymatic activity, which in turn inhibits the core fucosylation of P‐selectin glycoprotein ligand 1 (PSGL1). The disruption of ligand–receptor interaction and alterations in the glycosylation pattern made disorder regulation of PSGL1 on cytoskeleton, ultimately resulting in the death of ICC‐TRCs.

## 2 Results

### 2.1 Sulfarotene Selectively Inhibits ICC‐TRCs In Vitro and In Vivo

Our previous studies showed the potent inhibition of SFT against TRCs in various cancer types.^[^ ^11^, ^12^, ^13^ ^]^ Here, we found that SFT effectively inhibited the cell viability of RBE, HUCCT1, HCCC9810, and TFK1, with a half inhibitory concentration (IC_50_) being 3.83 ± 0.45, 7.11 ± 1.45, 9.08 ± 3.56, and 5.21 ± 0.57 µm, respectively (Table S1, Supporting Information). Its IC_50_ was lower than 5‐fluorouracil (5‐FU), cisplatin (CDDP), pemigatinib (PEM), and all‐trans retinoic acid (ATRA), but higher than gemcitabine (GEM) in 2D ICC cell lines. Further studies showed that SFT possessed a comprehensive effect against the biologic behaviors of 2D ICC cells, including inhibiting colony formation, proliferation, migration, invasion, and promoting apoptosis (Figures S1 and S2, Supporting Information). Taken overall, SFT exhibited superior inhibition against 2D ICC cells to ATRA, 5‐FU, CDDP, PEM, and was not inferior to GEM.

Then, the 3D fibrin gel was used to establish ICC‐TRCs.^[^ ^7^, ^15^, ^16^ ^]^ Our previous studies indicated the high level of stem cell markers in both HUCCT1‐TRCs and RBE‐TRCs, including EpCAM, SOX2, CD90, LGR5, and CD117.^[^ ^16^ ^]^ ICC‐TRCs were more invasive than 2D cells.^[^ ^16^ ^]^ Compared with 2D cells, flow cytometry indicated a higher proportion of EpCAM^+^ or LGR5^+^ cells in ICC‐TRCs (Figure S3A, Supporting Information), which were considered as highly tumorigenic cells.^[^ ^17^, ^18^ ^]^ Additionally, HUCCT1‐TRCs sampled at 5 × 10^5^ cells could form subcutaneous xenograft tumor nodes in BALB/c nude mice, but HUCCT1 failed at the same cell number (Figure S3B–D, Supporting Information). Together, ICC‐TRCs displayed a notable tumorigenic property.

**Figure 1:**
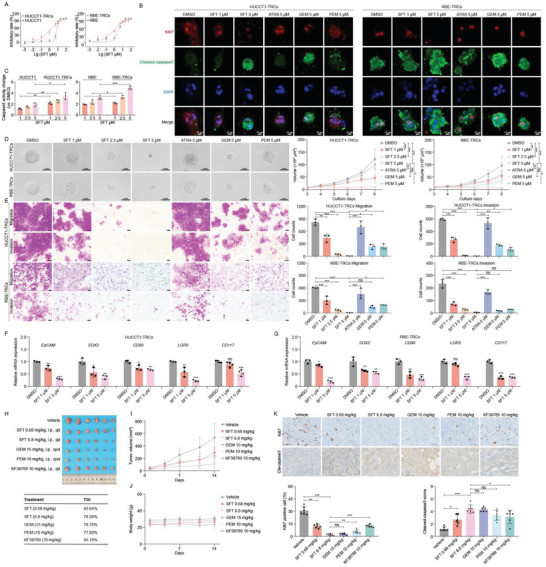
Sulfarotene selectively inhibits ICC‐TRCs. A) The IC_50_ values of SFT for ICC‐TRCs and 2D ICC cells were determined usingCCK8 assay after treatment for 48 h (*n*  =  3). B) Representative immunofluorescence images for the changes of Ki67 (red) and cleaved‐caspase3 (green) in the ICC‐TRCs in response to treatment with SFT (1, 5 µm), ATRA (5 µm), GEM (5 µm), and PEM (5 µm) for 48 h. The nuclear was stained with DAPI (blue). Scale, 10 µm. C) The caspase3 activity level of ICC‐TRCs in response to SFT was detected after treatment for 48 h (*t*‐test). D) Inhibition of colony spheroid growth in ICC‐TRCs. Left panel, the representative images of ICC‐TRCs colony spheroids on day 8 (treatment from day3). Right panel, quantitative analysis of time‐dependent changes of colony spheroid sizes (*n*  =  6, Tukey's multiple comparisons test). E) Effects of SFT, ATRA, GEM, and PEM on the migration and invasion of ICC‐TRCs. Left panel, the representative images. Right panel, quantitative analysis of Transwell assay calculated using Image J (*n* = 3, Tukey's multiple comparisons test). F,G) Effect of SFT on the expression of stemness markers in HUCCT1‐TRCs F) and RBE‐TRCs G). *n* = 3, Tukey's multiple comparisons test. H–J) Inhibitory effects of SFT on the growth of xenograft tumor of HUCCT1‐TRCs subcutaneously transplanted to the flanks of BALB/c nude mice (*n*  =  6). After tumor grew to 50 mm^3^, mice were treated with SFT (0.68 or 6.8 mg kg^−1^, i.p., qd) compared to GEM (15 mg kg^−1^, i.p., qod), PEM (10 mg kg^−1^, i.p., qod), and KF38789 (10 mg kg^−1^, i.p., qd) and the carrier. K) The effect of SFT on the expression of Ki67 and cleaved‐caspase3 in tumor tissues. Upper panel, the representative images. Down panel, quantitative analysis of IHC calculated using Image J (*n* = 6, Tukey's multiple comparisons test). ICC‐TRCs, tumor‐repopulating cells of intrahepatic cholangiocarcinoma; IC50, half inhibitory concentration; SFT, sulfarotene; ATRA, all‐trans retinoic acid; GEM, gemcitabine; PEM, pemigatinib; i.p., intraperitoneal injection; qd, once a day; qod, every other day; TGI, tumor growth inhibition. Data are presented as the mean ± SD; *p < 0.05, **p < 0.01, ***p < 0.001. ns, not significant.

The data of CCK8 assay showed that SFT exhibited an IC_50_ of 1.17 ± 0.44 and 1.53 ± 0.31 µm in RBE‐TRCs and HUCCT1‐TRCs after 48 h treatment, being ≈3.27 and ≈4.65 fold lower than it in parent 2D cells, but ICC‐TRCs showed resistant to chemotherapy drugs (**Figure** **1**A; and Table S1, Supporting Information). Of note, combination of SFT and 5‐FU, CDDP, or GEM showed a significantly more active on ICC‐TRCs than individual drug alone (Figure S4, Supporting Information), while the synergistic effect was not remarkable. It indicated the potential of combination therapy, but the optimum combination strategy needs to be further explored. As previously reported, SFT has S and R configuration, WYC‐209A and WYC‐209B.^[^ ^11^ ^]^ WYC‐209A and WYC‐209B also performed a better inhibition on ICC‐TRCs than 2D cells (Table S1, Supporting Information). The efficacy of WYC‐209A and WYC‐209B was nearly equivalent to SFT in ICC‐TRCs. Evaluation of cell proliferation and apoptosis biomarkers uncovered that SFT significantly reduced the expression of Ki67 but increased cleaved‐caspase3 in ICC‐TRCs compared with dimethylsulfoxide (DMSO; Figure 1B). It also dose‐dependently increased the caspase3 activity in both RBE‐TRCs and HUCCT1‐TRCs, being superior to ATRA, PEM, and not inferior to GEM (Figure S2, Supporting Information). It performed a more significant activation of caspase3 activity in ICC‐TRCs than in parent 2D cells (Figure 1C). These data showed a more potent inhibition of SFT against ICC‐TRCs than 2D ICC cells.

Next, the effects of SFT was investigated on the growth and formation of colony spheroids from ICC‐TRCs. Colony spheroids of ICC‐TRCs were markedly inhibited by SFT at concentrations of 1, 2.5, and 5 µm, which was superior to ATRA, PEM, and GEM at same concentration (Figure 1D). SFT also dose‐dependently inhibited the formation of colony spheroids from ICC‐TRCs (Figure S5A, Supporting Information). The growth of colony spheroids treated with SFT for 48 h was still significantly damaged even after eluting SFT (Figure S5B, Supporting Information). Additionally, SFT effectively blocked the migration and invasion of ICC‐TRCs, and it achieved a more potent inhibition than the same concentration of ATRA, PEM, and GEM (Figure 1E). SFT also significantly decreased the expression of EpCAM, SOX2, CD90, LGR5, and CD117 in ICC‐TRCs (Figure 1F,G). OF note, both WYC‐209A and WYC‐209B performed an equivalent inhibition to SFT on the growth of colony spheroids of ICC‐TRCs (Figure S6, Supporting Information).

Before in vivo estimation of SFT, we conducted a drug metabolism and pharmacokinetics (DMPK) analysis for SFT. Results from Caco‐2 permeability assay showed that apparent permeability coefficient of apical to basolateral (A to B) transport was 0.63 cm s^−1^, and basolateral to apical (B to A) transport was 0.19 cm s^−1^, indicating a moderate absorption rate of SFT in the intestine (Table S2, Supporting Information). In vitro metabolites identification (MID) conducted in liver microsome and liver cells demonstrated that SFT could be widely metabolized, and its metabolites in the human system can be detected in at least one other animal species (monkey, dog, rat, or mouse; Table S3, Supporting Information). In vivo MID in rats could also identify a hydrolysis metabolite M15, which has been detected in the vast majority of in vitro system (Table S3, Supporting Information). We next evaluated the pharmacokinetics of SFT in rats. The concentration of SFT was lower than 1 ng mL^−1^ in rats with oral administration, and could be only detected within 0.25 h in rats with intravenous injection (iv). However, chromatographic peaks between 309.0696 and 341.0959 could be extracted using ions of carboxylic acid metabolites in above MID (Figure S7A, Supporting Information). And these metabolites exhibited a high stability in the blood of rats (Figure S7B, Supporting Information). SFT belongs to ester compounds, and thereby we inferred that it may act as a pro‐drug that needs to be converted into acids for its activity. We also estimated the toxicity of SFT in rats. Oral administration or iv of SFT displayed a well biosafety (Table S4, Supporting Information).

In view of the excellent in vitro activity, stability, and biosafety, SFT's activity was evaluated in a xenograft tumor model. This in vivo estimation showed that SFT decreased volumes and weights of tumor nodes, with no significant alterations on body weight (Figure 1H,J). Among which, SFT administration every day at a concentration of 6.8 mg kg^−1^ achieved a significant inhibition comparable to PEM or GEM administration. Immunohistochemistry (IHC) of cell proliferation and apoptosis biomarkers revealed that SFT significantly decreased Ki67 but increased cleaved‐caspase3 in tumor tissues (Figure 1K).

In addition, the activity of SFT was evaluated on mouse cell line AY‐LTC2.^[^ ^19^ ^]^ SFT displayed a more potent inhibition against AY‐LTC2‐TRCs than AY‐LTC2, so did WYC‐209A and WYC‐209B (Table S1, Supporting Information). SFT, WYC‐209A, and WYC‐209B also dose‐dependently inhibit the growth of colony spheroids of AY‐LTC2‐TRCs (Figure S8A, Supporting Information). Additionally, we used AY‐LTC2‐TRCs to construct the tumor‐bearing C57BL/6 mice, and treated with SFT, WYC‐209A, and WYC‐209B. After treatment, SFT showed a dose‐dependently inhibition on tumor growth (Figure S8B,C, Supporting Information). WYC‐209A and WYC‐209B showed a similar tumor shrinkage to the same concentration of SFT. SFT, WYC‐209A, and WYC‐209B had no impact on mouse weight (Figure S8D, Supporting Information). Taken together, SFT leads to a robust suppression on ICC‐TRCs in vitro and in vivo.

### 2.2 Sulfarotene Modulates the Cellular Location Rather than Expression of RARα

**Figure 2:**
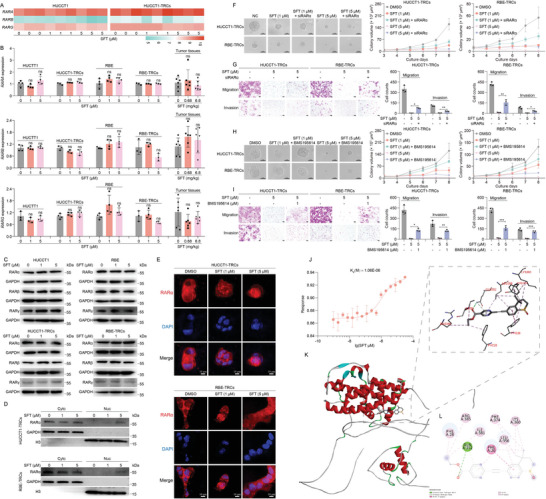
Sulfarotene induces the nuclear translocation of RARɑ in ICC‐TRCs. A) RNA‐Seq analysis revealed that SFT treatment had no significant effects on the mRNA expression of RARα, RARβ, and RARγ in HUCCT1‐TRCs (*n* = 3). B) qRT‐PCR showed that SFT treatment had no significant effects on the mRNA expression of RARα, RARβ, and RARγ in ICC‐TRCs (*n* = 3), 2D ICC cells (*n* = 3), and tumor tissues (*n* = 6). Tukey's multiple comparisons test. C) IB demonstrated that SFT treatment (48 h) had no significant effects on the protein level of RARα, RARβ, and RARγ in ICC‐TRCs as well 2D ICC cells. D) The cytosolic and nuclear fractions of HUCCT1‐TRCs and RBE‐TRCs after SFT treatment for 48 h were isolated by nuclear and cytoplasmic protein extraction kit. The RARα protein level in each fraction was detected using IB. E) Representative immunofluorescence images for the changes of RARα in the ICC‐TRCs with the treatment of SFT (48 h). Scale, 10 µm. F,G) Effects of SFT and RARα siRNA on the colony spheroid formation, migration and invasion ability of ICC‐TRCs (*n* = 3, Tukey's multiple comparisons test). H,I) Effects of SFT and RARα antagonist (BMS195614) on the colony spheroid formation, migration and invasion ability of ICC‐TRCs (*n* = 3, Tukey's multiple comparisons test). J) MST analysis of SFT binding to human recombinant RARα protein. K,L) Representative 3D K) and 2D L) structural diagram of autodocking for SFT and RARα. ICC‐TRCs, tumor‐repopulating cells of intrahepatic cholangiocarcinoma; SFT, sulfarotene; qRT‐PCR, quantitative reverse transcription polymerase chain reaction; IB, immunoblotting; MST, microscale thermophoresis. Data are presented as the mean ± SD; *p < 0.05, **p < 0.01, ***p < 0.001. ns, not significant.

In HCC‐TRCs, SFT could promote the expression of RARɑ and induce the nuclear translocation of RARɑ.^[^ ^12^ ^]^ However, data from RNA‐seq, quantitative reverse transcription polymerase chain reaction (qRT‐PCR), and immunoblotting (IB) showed no significant impacts of SFT on the expression of RARɑ in ICC‐TRCs as well 2D cells, nor the expression of RARβ and RARγ (**Figure** **2**A–C). The cellular location analysis showed that SFT obviously increased the level of nuclear RARɑ in both HUCCT1‐TRCs and RBE‐TRCs (Figure 2D). Compared with 2D ICC cells, the level of RARɑ mRNA and proteins have no obvious changes in ICC‐TRCs, yet the nuclear RARɑ was visibly decreased in ICC‐TRCs (Figure S9, Supporting Information). The data of immunofluorescence (IF) also agreed above results (Figure 2E; and Figure S9D, Supporting Information). These data indicated that the major difference between ICC‐TRCs and 2D cells was the cellular location but not expression of RARɑ, and SFT only changed its location.

Although SFT treatment significantly suppressed the growth of colony spheroids in ICC‐TRCs, as well as migration and invasion in vitro, silencing the expression of RARɑ by siRNA could significantly reverse these inhibitory effects (Figure 2F,G), which markedly decreased the mRNA and protein levels of RARα (Figure S10, Supporting Information). Consistently, pre‐treatment of BMS195614 (a selective RARα activation antagonist) also rescued the inhibition of SFT on the colony spheroid growth as well as the migration and invasion (Figure 2H,I), indicating that RARα is a potential target of SFT in ICC‐TRCs.

To explore the action mode of SFT against RARɑ, we expressed and purified the recombinant human RARɑ protein (Figure S11, Supporting Information). Then, we carried out microscale thermophoresis (MST) with RARɑ protein and a dilution series of SFT. As expected, RARɑ was readily bound by SFT, with a dissociation constant (*K*_d_) value of 1.06E‐06 (Figure 2J). Additionally, data of molecular docking assay showed that the minimum binding energy was −7.6 kcal mol^−1^ between SFT and RARɑ. In detail, SFT could form hydrogen bond with GLN352 and pi–pi T shaped interaction with PHE26, PHE28, LEU356, LYS360, PHE374, ILE381, ARG385 of RARɑ (Figure 2K,L). These data were suggestive of a strong and directed interaction between SFT and RARɑ. Taken together, RARɑ is a directed target for SFT's inhibition against ICC‐TRCs.

### 2.3 Sulfarotene Inhibits P‐Selectin Transcription via Modulating RARα

**Figure 3:**
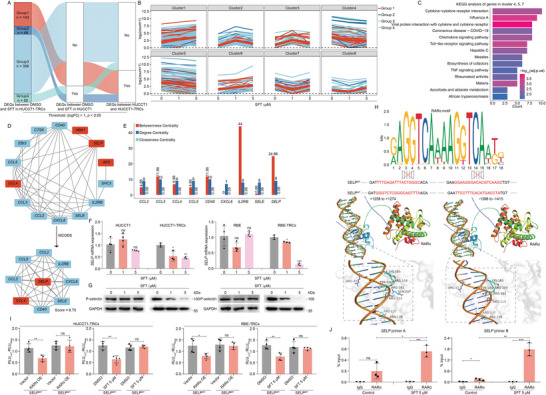
Sulfarotene decreases the transcription of P‐selectin via RARɑ in ICC‐TRCs. A) The sankey plot showed the two classification methods for DEGs. B) The trend of DEGs with increased SFT. C) KEGG analysis of genes from cluster 4, 5, 7. D) PPI and MCODE algorithm of key DEGs. E) Centrality analysis of the hub genes. F,G) ICC‐TRCs were treated with SFT for 48 h. The expression of P‐selectin was detected by using qRT‐PCR (F, *n* ≥ 3, Tukey's multiple comparisons test) and IB G). H) The binding site between RARα motif and *SELP* gene was predicted using JASPAR database and computer simulation docking. I) The transcriptional activity of *SELP* was detected using dual luciferase reporter assay before and after binding site mutation (*n* = 4, *t*‐test). J) Quantitative ChIP‐PCR was utilized to detect the binding of RARɑ at promoter regions of SELP in HUCCT1‐TRCs. IgG served as a negative control. Enrichment relative to the input was shown (*n* = 3, *t*‐test). ICC‐TRCs, tumor‐repopulating cells of intrahepatic cholangiocarcinoma; SFT, sulfarotene; DEGs, differential expression genes; KEGG, Kyoto Encyclopedia of Genes and Genomes; PPI, protein–protein interaction; MCODE, molecular complex detection; qRT‐PCR, quantitative reverse transcription polymerase chain reaction; IB, immunoblotting; ChIP‐PCR, chromatin immunoprecipitation PCR. Data are presented as the mean ± SD; *p < 0.05, **p < 0.01, ***p < 0.001. ns, not significant.

RARɑ belongs to the nuclear receptor superfamily, and primarily influences cancer biology via regulating target gene transcription.^[^ ^20^ ^]^ The EdgeR package of R was employed to perform a differential gene analysis on RNA‐seq data. Setting the threshold as |log_2_(Fold change)| (|logFC|) > 1 and *p* value < 0.05, a total of 279 and 392 differentially expressed genes (DEGs) were obtained after 1 and 5 µm SFT treatment in HUCCT1‐TRCs, respectively (Figure S12, Supporting Information). In totally, SFT treatment yielded 597 DEGs in HUCCT1‐TRCs (Figure S12D, Supporting Information). These 597 DEGs were divided into four groups based on whether they significantly changed between HUCCT1 and HUCCT1‐TRCs, and in SFT‐treated HUCCT1 (**Figure** **3**A). Among that, the genes (*n* = 143) included in group1 that was significantly different between HUCCT1 and HUCCT1‐TRCs but not in SFT‐treated HUCCT1 may be critical for the targeted inhibition of SFT against HUCCT1‐TRCs. Additionally, the dynamic changes of 597 DEGs were classified into 8 patterns with pheatmap package of R. Cluster 4, 5, and 7 showed a decreased trend with the increase of SFT concentrations (Figure 3B). Kyoto Encyclopedia of Genes and Genomes (KEGG) analysis showed the functions of genes in cluster 4, 5, and 7 were enriched in several cancer‐related pathways, such as cytokine–cytokine receptor interaction, chemokine signaling pathway, Toll‐like receptor signaling pathway, tumor necrosis factor (TNF) signaling pathway and hepatitis C (Figure 3C). We conducted a protein–protein interaction (PPI) analysis and molecular complex detection (MCODE) algorithm on these functionally enriched genes, which obtained a core module containing 9 genes (*CCL2*, *CCL3*, *CCL4*, *CCL5*, *CXCL6*, *IL2RB*, *SELE*, *SELP*, *CD40*; Figure 3D). Subsequently, centrality analysis achieved by Cytoscape indicated the central role of *SELP* and *IL2RB* among all DEGs according to betweenness centrality, degree centrality, and closeness centrality (Figure 3E). Of note, *SELP* belongs to group1 while *IL2RB* to group3. Hence, we focused on *SELP*, a coding gene of P‐selectin, belonging to the selectin family.^[^ ^21^ ^]^ The level of *SELP* mRNA was significantly increased in ICC‐TRCs compared to parent 2D cells (Figure S13A, Supporting Information). P‐selectin was also up‐regulated in ICC‐TRCs or HUCCT1‐TRCs formed tumor tissues (Figure S13B,C, Supporting Information). Additionally, SFT caused a significant decrease of P‐selectin in ICC‐TRCs at mRNA and protein levels, with no impact observed in 2D ICC cells (Figure 3F,G). SFT also significantly decreased the level of P‐selectin in tumor tissues (Figure S14, Supporting Information). These data suggested that *SELP* may be the critical gene for SFT's inhibition against ICC‐TRCs.

Based on the JASPAR database (https://jaspar.genereg.net/), typical RARα binding elements located at −1398 to −1415 (TTCCTTTCACATGACCTA) and +1258 to +1274 (GGGTCTCGGGCAGTTTA) bp were sought for *SELP* promoter region, respectively (Figure 3H). Simulated docking using HDOCK Server illustrated a functional binding contact between the critical amino acids (ARG83, TYR100, ARG113, ARG137, ASN138, ARG159, ARG162, and LYS165) in the DNA‐binding domain of RARα and the loci of −1398 to −1415 or +1258 to +1274 in *SELP* gene, with a docking score of −221.55 or −238.89, and a confidence score of 0.81 or 0.86, respectively (Figure 3H). A dual luciferase reporter assay indicated that RARα over‐expression or SFT treatment reduces the luciferase activity driven by the wild‐type *SELP* promoter. Mutation of these two sequences successfully rescued the reduction of *SELP* expression induced by RARα over‐expression or SFT treatment (Figure 3I). Additionally, the data of chromatin immunoprecipitation PCR (ChIP‐PCR) showed the binding potential of RARα to *SELP* gene (Figure 3J; and Figure S15, Supporting Information). Taken together, *SELP* is a direct transcriptional target of RARα targeted by SFT.

### 2.4 P‐Selectin Drives an Oncogenic Factor in ICC‐TRCs Through Regulating Cytoskeleton

**Figure 4:**
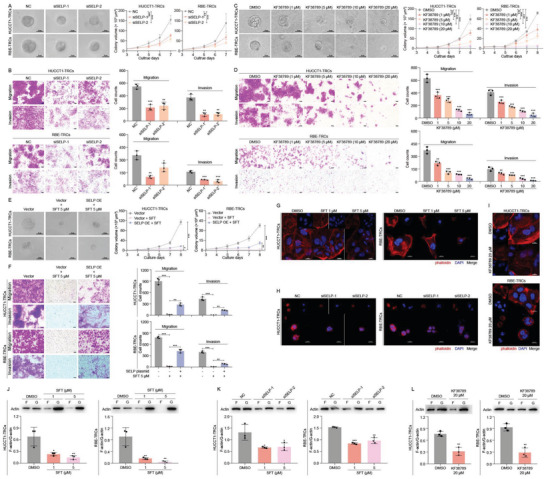
Decreasing P‐selectin destroys the cytoskeleton of ICC‐TRCs. A) Inhibition of colony spheroid formation by silencing P‐selectin in ICC‐TRCs. Left panel, the representative images of ICC‐TRCs colony spheroids on day 7. Right panel, quantitative analysis of time‐dependent changes of colony spheroid sizes (*n*  =  3, *t*‐test). B) Effects of silencing P‐selectin on the migration and invasion of ICC‐TRCs. Left panel, the representative images. Right panel, quantitative analysis of Transwell assay calculated using Image J (*n* = 3, *t*‐test). C) Inhibition of colony spheroid formation by KF38789 (P‐selectin inhibitor) in ICC‐TRCs. Left panel, the representative images of ICC‐TRCs colony spheroids on day 8 (treatment from day 3). Right panel, quantitative analysis of time‐dependent changes of colony spheroid sizes (*n*  =  3, Tukey's multiple comparisons test). D) Effects of KF38789 on the migration and invasion of ICC‐TRCs. Left panel, the representative images. Right panel, quantitative analysis of Transwell assay calculated using Image J (*n* = 3, Tukey's multiple comparisons test). E,F) Over‐expressing P‐selectin rescued the inhibition of SFT on colony spheroid formation, migration and invasion in ICC‐TRCs (*n* = 3, Tukey's multiple comparisons test). G–I) Phalloidin staining showed the inhibition of SFT G), siSELP H), or KF38789 I) on F‐actin of ICC‐TRCs. Red, phalloidin; blue, DAPI. Scale, 20 µm. J–L) F‐actin and G‐actin was separated by using G‐actin/F‐actin in vivo assay kit. Immunoblotting was used to investigate the alteration of F‐/G‐actin induced by SFT J), siSELP K), or KF38789 L) treatment (*n* = 3, Tukey's multiple comparisons test or *t*‐test). ICC‐TRCs, tumor‐repopulating cells of intrahepatic cholangiocarcinoma; SFT, sulfarotene; IB, immunoblotting. Data are presented as the mean ± SD; *p < 0.05, **p < 0.01, ***p < 0.001.

P‐selectin consists of 789 amino acid residues and is structured into three parts: N‐terminal extracellular domain (730 amino acid residues) contains a lectin‐like domain, an epidermal growth factor like domain and nine complement regulatory protein consensus repeat units; Transmembrane region is composed of 24 amino acid residues; C‐terminal intracellular domain contains 35 amino acid residues.^[^ ^21^ ^]^ Silencing P‐selectin significantly decreased the growth of colony spheroids, migration as well invasion in ICC‐TRCs (Figure S16 (Supporting Information); and **Figure** **4**A,B). KF38789 is a selective P‐selectin inhibitor. It dose‐dependently inhibited the growth of colony spheroids, migration, and invasion in ICC‐TRCs (Figure 4C,D). Additionally, KF38789 decreased the volumes and weights of HUCCT1‐TRCs formed tumor nodes in vivo. It reduces the level of Ki67, but increases cleaved‐caspase3 (Figure 1I,L). These data suggested the driving role of P‐selectin in ICC‐TRCs. We next transfected ICC‐TRCs with *SELP* plasmid (Figure S17, Supporting Information). Exogenous P‐selectin weakened the inhibition of SFT against ICC‐TRCs (Figure 4E,F). Additionally, silencing P‐selectin also decreased the inhibition of SFT against ICC‐TRCs (Figure S18, Supporting Information), while it was modest. It may be due to the high level of P‐selectin in ICC‐TRCs, even after interfered by siRNA. Taken together, inhibition of P‐selecting accounts for SFT's selective suppression against ICC‐TRCs.

P‐selectin could trigger intracellular calcium flux and other cancer‐related signaling, such as AKT.^[^ ^22^, ^23^ ^]^ Accordingly, we first investigated the effect of SFT on these events, but SFT had no significant inhibition on the intracellular calcium flux and phosphorylation of AKT (Figure S19, Supporting Information). Previous studies indicated that the binding of P‐selectin to PSGL1 could facilitate the growth and metastasis of cancer.^[^ ^21^, ^24^ ^]^ Worthy of note is that PSGL1 directly binds to cytoskeleton via ezrin/radixin/moesin (ERM) complex.^[^ ^25^ ^]^ Cytoskeleton‐related components, cytoskeleton remodeling as well as its interaction with CSCs play important roles in maintaining and promoting the biological behavior of CSCs.^[^ ^26^ ^]^ We found that SFT decreased the level of filamentous‐actin (F‐actin) in ICC‐TRCs (Figure 4G). And both silencing or inhibiting P‐selectin remarkedly reduced the level of F‐actin in ICC‐TRCs (Figure 4H,I). Additionally, SFT significantly reduced the ratio of F‐actin/globular‐actin (F/G‐actin) in ICC‐TRCs, and so did silencing or inhibiting P‐selectin (Figure 4J,L). The ratio of F/G‐actin was also declined in SFT or KF38789 treated tumor tissues (Figure S20, Supporting Information). While silencing or inhibiting RARɑ, or over‐expressing *SELP* effectively rescued SFT‐induced decrease of the ratio of F/G‐actin in ICC‐TRCs (Figure S21, Supporting Information). As a consequence, SFT destroyed the cytoskeleton of ICC‐TRCs via RARɑ mediated down‐regulation of P‐selectin.

### 2.5 Sulfarotene Inhibits the N‐Glycosylation of PSGL1

**Figure 5:**
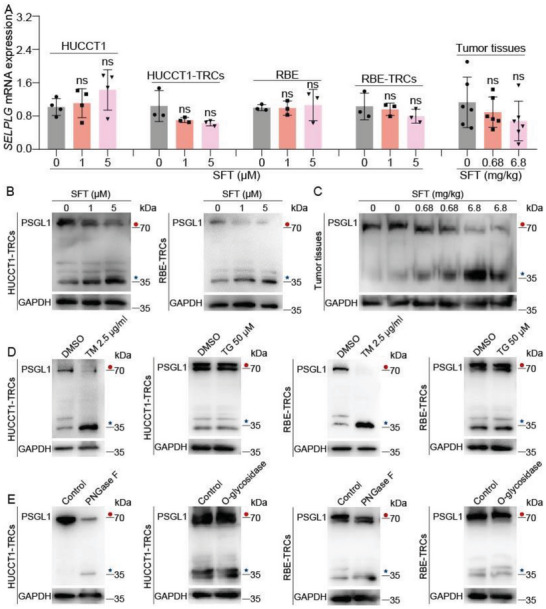
Sulfarotene inhibits the N‐glycosylation of PSGL1 in ICC‐TRCs. A) The level of PSGL1 mRNA was detected using qRT‐PCR (*n* = 3 or 6, Tukey's multiple comparisons test). B) HUCCT1‐TRCs or RBE‐TRCs were treated with SFT for 48 h, and then PSGL1 was detected using IB. C) Total proteins were exacted from tumor tissues and detected by IB. D) HUCCT1‐TRCs or RBE‐TRCs were treated with TM (2.5 µg mL^−1^) or TG (50 µm) for 48 h. E) Cell lysates from HUCCT1‐TRCs or RBE‐TRCs were treated with PNGase F or O‐glycosidase for 1 h at 37 °C in vitro. ICC‐TRCs, tumor‐repopulating cells of intrahepatic cholangiocarcinoma; SFT, sulfarotene; IB, immunoblotting; TM, tunicamycin; TG, Thiamet G; PNGase F, recombinant peptide‐*N*‐glycosidase. F) Data are presented as the mean ± SD; ns, not significant.

During the study, we found that SFT treatment elicited a stronger inhibition on ICC‐TRCs than silencing or inhibiting P‐selectin, implying a more complex mechanism of action. Given that the significant interference of SFT on cytoskeleton, we evaluated the directed impact of SFT on PSGL1, but the data showed that SFT had no significant inhibition on *SELPLG* (the encoding gene of PSGL1) mRNA in ICC‐TRCs or tumor tissues (**Figure** **5**A). Then, PSGL1 protein expression was detected in ICC‐TRCs and tumor tissues. We noticed that PSGL1 was mainly detected at ≈70 kDa (red closed circle) and ≈35 kDa (blue asterisk) (Figure 5B,C). Further data showed that a significant portion of the ≈70 kDa PSGL1 was reduced to ≈35 kDa upon SFT treatment (Figure 5B,C). PSGL1 is a well‐known glycoprotein, so we inferred that SFT may change the glycosylation pattern of PSGL1. To identify the possible glycosylation pattern, we used N‐linked glycosylation inhibitor tunicamycin (TM) and O‐glycosidase inhibitors Thiamet G (TG) to treat ICC‐TRCs. When used TM in ICC‐TRCs, glycosylation of PSGL1 was also blocked and the mobility of PSGL1 reduced from ≈70 to ≈35 kDa, but TG failed (Figure 5D). In addition, the glycosylation of endogenous PSGL1 was similarly altered when HUCCT1‐TRCs and RBE‐TRCs cell lysates were treated with recombinant peptide‐*N*‐glycosidase F (PNGase F) glycosidase that can remove the entire *N*‐glycan structure, but recombinant O‐glycosidase could not cause same alteration (Figure 5E). Hence, we inferred that SFT treatment may inhibited the N‐glycosylation of PSGL1 in ICC‐TRCs.

### 2.6 FUT8 is a Direct Target of SFT in ICC‐TRCs

Glycosylation is an enzymatic process in the endoplasmic reticulum/Golgi apparatus of cells, which is one of the major ways of protein post‐translational modification catalyzed by different glycosyltransferases and/or glycosidases.^[^ ^27^ ^]^ In view of the influence of SFT on RARɑ, we first inferred that it may act as a transcriptional regulation on glycosyltransferase/glycosidase via increasing nuclear traffic RARɑ. Accordingly, the genes encoding human glycosyltransferase and glycosidase were downloaded from the GlycoGene Database (GGDB).^[^ ^28^ ^]^ After intersection, we focused on *MGAT3* (Figure S22A, Supporting Information), an encoding gene of N‐acetylglucosamine transferase III, which links N‐acetylglucosamine to β‐mannose of N‐glycan trisaccharide core through a β1‐4 glycosidic bond and thereby catalyzes the formation of a bisecting GlcNAc structure in N‐glycans.^[^ ^29^ ^]^ Data from RNA‐seq and qRT‐PCR showed that SFT treatment significantly decreased the level of *MGAT3* mRNA in ICC‐TRCs, but not in 2D ICC cells (Figure S22B,C, Supporting Information). Hence, siRNA was used to silence *MGAT3* in ICC‐TRCs (Figure S23A,B, Supporting Information). However, silencing *MGAT3* failed to inhibit the glycosylation of PSGL1, growth of colony spheroids, migration and invasion in ICC‐TRCs (Figure S23C,D, Supporting Information). These results indicated that the changes of *MGAT3* are only additional effects after SFT treatment, but not the reason for SFT's inhibition on ICC‐TRCs.

**Figure 6:**
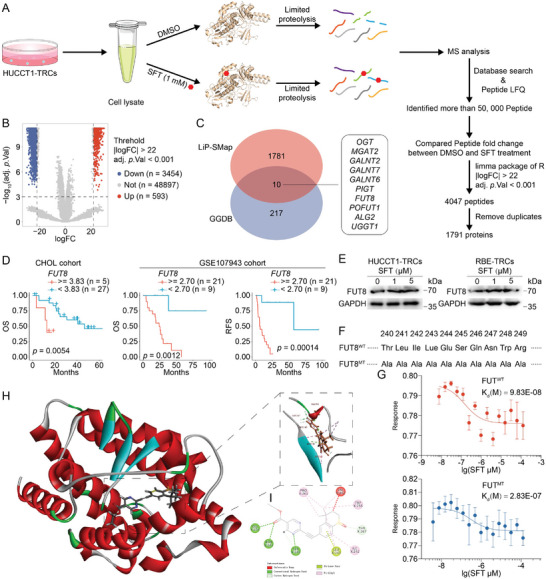
Sulfarotene directly binds to FUT8. A) Flow chart depicts the Lip‐SMap assay. Freshly prepared whole‐cell lysates from HUCCT1‐TRCs were treated with DMSO or SFT (1 mm) followed by proteinase K digestion and analysis by MS. The binding of SFT prevents proteinase K digestion, leading to the differential MS peptide profiling. B) Volcano plot displayed the differential MS peptide profiling of Lip‐SMap assay. C) Venn plot showed the intersection between the differential MS peptides and glycosyltransferase. D) Survival analysis of FUT8 in CHOL and GSE107943 cohort (Log‐rank test). E) HUCCT1‐TRCs or RBE‐TRCs were treated with SFT for 48 h, and then FUT8 was detected using immunoblotting. F) The mutated site in FUT8^MT^. G) MST assay of SFT binding to FUT8^WT^ or FUT8^MT^. H,I) Representative 3D H) and 2D I) structural diagram of autodocking for SFT and FUT8. Lip‐SMap, limited proteolysis‐mass spectrometry; TRCs, tumor‐repopulating cells; SFT, sulfarotene; MS, mass spectrometry; OS, overall survival; RFS, relapse‐free survival; MST, microscale thermophoresis.

The transcriptional influence on glycosyltransferase or glycosidases is hard to explain the effect of SFT in ICC‐TRCs, so it may act on other target beyond RARα. Limited proteolysis mass spectrometry (Lip‐SMap) is a high‐throughput method based on mass spectrometry (MS) for the interaction between small molecule drugs and proteins.^[^ ^30^ ^]^ Total proteins from HUCCT1‐TRCs was extracted and treated with DMSO or SFT (1 mm). After protease K digestion, the samples were analyzed by MS (**Figure** **6**A). Over 50 000 peptide segments were analyzed using a label free quantification (LFQ) conducted by Maxquant soft, which identified 4047 targets after differential analysis with a threshold of |logFC| > 22 and adj.*p*.val < 0.001 (Figure 6A,B). After removing duplicate, bioinformatics analysis designated that these 4047 peptides represented 1791 different proteins (Figure 6A,B). Subsequently, the UniProt database (https://www.uniprot.org) was used to transform proteins into corresponding encoding genes. After intersecting them with glycosyltransferase or glycosidase encoding genes, a total of 10 potential direct targets were obtained, including OGT, MGAT2, GALNT2, GALNT6, GALNT7, PIGT, FUT8, POFUT1, ALG2, and UGGT1 (Figure 6C; and Table S5, Supporting Information). Data of RNA‐seq indicated no significant impacts on the expression of these genes after SFT treatment (Figure S22B, Supporting Information). We inferred that SFT may regulate enzyme activity by directly binding to them.

To confirm the direct target, we evaluated the importance of the 10 potential targets in ICC. Differential analysis showed that OGT, GALNT2, GALNT6, GALNT7, PIGT, FUT8, POFUT1, ALG2, and UGGT1 were up‐regulated in ICC tissues than in normal tissues (Figure S24A,B, Supporting Information). Survival analysis showed that only FUT8 was significantly associated with OS and relapse‐free survival (RFS) in ICC (Figure 6D; and Figure S24C–E, Supporting Information). SFT treatment could not cause a significant change on the mRNA or protein of FUT8 (Figure 6E; and Figure S22B, Supporting Information). Based on the results of Lip‐SMap, SFT may bind to the segment from 240 to 249 (THR‐LEU‐ILE‐LUE‐GLU‐SER‐GLN‐ASN‐TRP‐ARG) of FUT8 (Table S5, Supporting Information). Accordingly, we constructed a mutant FUT8 via replacing these 10 residues with ALA (Figure 6F), and purified FUT8^WT^ and FUT8^MT^ proteins (Figure S25, Supporting Information). The data of MST showed a potent affinity of SFT with FUT8^WT^ protein, but a decreased affinity with FUT8^MT^ protein, with a *K*_d_ value of 9.83E‐08 and 2.83E‐07, respectively (Figure 6G). Simulated docking data showed that SFT formed stable hydrogen bonding interactions with SER245, ASN247, GLY253, THR267, and hydrophobic interactions with ILE242, TRP255, PRO261 (Figure 6F). Taken together, SFT may directly bind to the FUT8 in ICC‐TRCs.

**Figure 7:**
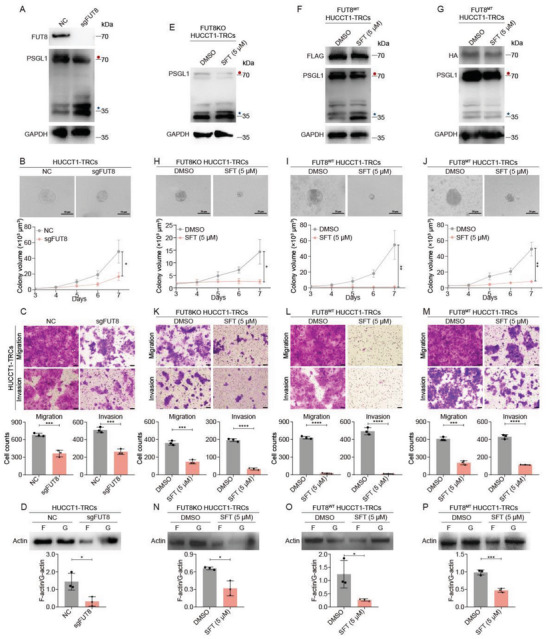
Sulfarotene inhibits ICC‐TRCs via binding to FUT8. A) The alternation of PSGL1 in FUT8KO HUCCT1‐TRCs. B–D) The effect of FUT8 knockout on colony spheroid growth B), migration and invasion C), F‐/G‐actin ratio D) in HUCCT1‐TRCs (*n* = 3, *t*‐test). E–G) FUT8KO HUCCT1‐TRCs, FUT8^WT^ HUCCT1‐TRCs, and FUT8^MT^ HUCCT1‐TRCs were treated with SFT for 48 h, and then PSGL1 was detected. H–J) Inhibition of SFT on colony spheroid growth in FUT8KO HUCCT1‐TRCs H), FUT8^WT^ HUCCT1‐TRCs I), and FUT8^MT^ HUCCT1‐TRCs J). Up panel, the representative images of ICC‐TRCs colony spheroids on day 7 (treatment from day3). Down panel, quantitative analysis of time‐dependent changes of colony spheroid sizes (*n*  =  3, *t*‐test). K–M) Inhibition of SFT on migration and invasion in FUT8KO HUCCT1‐TRCs K), FUT8^WT^ HUCCT1‐TRCs L), and FUT8^MT^ HUCCT1‐TRCs M). Up panel, the representative images. Down panel, quantitative analysis of Transwell assay calculated using Image J (*n* = 3, *t*‐test). N–P) The alteration of F‐/G‐actin ratio induced by SFT in FUT8KO HUCCT1‐TRCs N), FUT8^WT^ HUCCT1‐TRCs O), and FUT8^MT^ HUCCT1‐TRCs P). ICC‐TRCs, tumor‐repopulating cells of intrahepatic cholangiocarcinoma; SFT, sulfarotene. Data are presented as the mean ± SD; *p < 0.05, **p < 0.01, ***p < 0.001.

To investigate its role in ICC‐TRCs, we depleted FUT8 using specific single‐guide RNAs (sgRNAs) in HUCCT1‐TRCs (**Figure** **7**A). The loss of FUT8 significantly inhibited the growth of colony spheroids, migration, and invasion, and decreased F‐actin fluorescence intensity and F/G‐actin ratio in HUCCT1‐TRCs (Figure 7B–D). It also resulted in a decrease of glycosylated PSGL1 protein expression in HUCCT1‐TRCs (Figure 7A). In addition, we transfected plasmid with an empty vector, FUT8^WT^ or FUT8^MT^ into FUT8KO HUCCT1‐TRCs (Figure S26, Supporting Information). We found that SFT treatment can only reduce PSGL1 from ≈70 to ≈35 kDa in FUT8^WT^ HUCCT1‐TRCs, rather than in FUT8KO HUCCT1‐TRCs, or FUT8^MT^ HUCCT1‐TRCs (Figure 7E,G). Additionally, SFT treatment elicited a most potent inhibition on colony spheroids, migration, and invasion, as well as F/G‐actin ratio in FUT8^WT^ HUCCT1‐TRCs, while a modest inhibition in FUT8KO HUCCT1‐TRCs or FUT8^MT^ HUCCT1‐TRCs (Figure 7H,P). Taken together, inhibiting the activity of FUT8 is another mechanism for SFT's inhibition against ICC‐TRCs.

### 2.7 SFT Inhibits Core Fucosylation of PSGL1 in ICC‐TRCs

**Figure 8:**
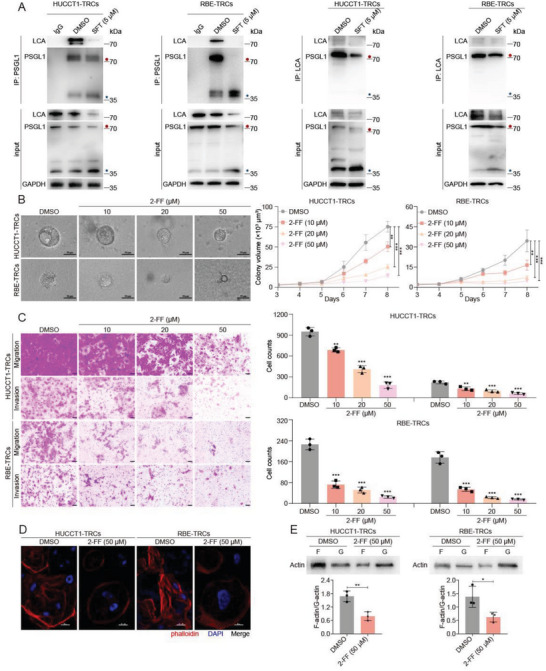
Sulfarotene inhibits the core fucosylation of PSGL1. A) HUCCT1‐TRCs or RBE‐TRCs were treated with SFT (5 µm) for 48 h, and followed PSGL1 antibody or LCA was used to pull down proteins. IB or lectin blotting was employed to detect the level of PSGL1 or core fucose structure within the pull‐down products. B) Inhibition of 2‐FF on colony spheroid growth in HUCCT1‐TRCs and RBE‐TRCs. Left panel, the representative images of colony spheroids on day 8 (treatment from day3). Right panel, quantitative analysis of time‐dependent changes of colony spheroid sizes (*n*  =  3, Tukey's multiple comparisons test). C) Effects of 2‐FF on the migration and invasion of HUCCT1‐TRCs and RBE‐TRCs. Left panel, the representative images. Right panel, quantitative analysis of Transwell assay (*n* = 3, Tukey's multiple comparisons test). D) Phalloidin staining showed the inhibition of 2‐FF on F‐actin of HUCCT1‐TRCs and RBE‐TRCs. Red, phalloidin; blue, DAPI. Scale, 20 µm. E) G‐actin/F‐actin in vivo assay kit and IB was used to investigate the alteration of F‐/G‐actin induced by 2‐FF treatment (*n* = 3, *t*‐test). TRCs, tumor‐repopulating cells; SFT, sulfarotene; 2‐FF, 2F‐Peracetyl‐Fucose; IB, immunoblotting. Data are presented as the mean ± SD; *p < 0.05, **p < 0.01, ***p < 0.001.

**Figure 9:**
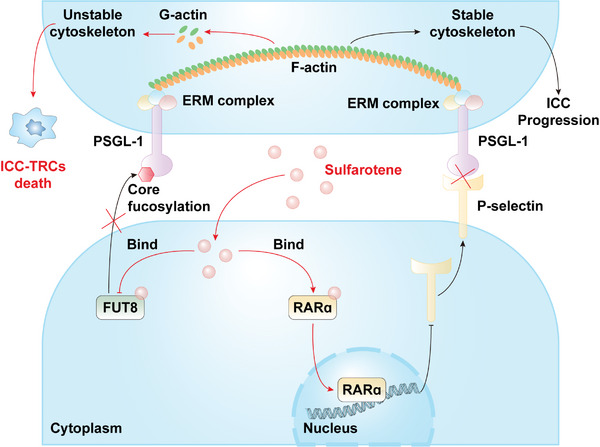
The action mode of sulfarotene in ICC‐TRCs.

*FUT8* encodes fucosyltransferase 8, the only enzyme for core fucosylation in human, which promotes the binding of fucose to the innermost *N*‐acetylglucosamine (GlcNAc) of *N*‐glycans with a ɑ‐1,6 bond.^[^ ^31^ ^]^ As the lectin from Lens culinaris agglutinin (LCA) specifically recognizes the ɑ‐1,6‐fucosylated trimannosyl‐core structure of N‐linked oligosaccharides,^[^ ^31^ ^]^ this reagent was employed to confirm whether SFT directly inhibited the core fucosylated of PSGL1. In HUCCT1‐TRCs and RBE‐TRCs, coimmunoprecipitation (CoIP) of PSGL1 followed by LCA blot showed reduced LCA binding to PSGL1 after SFT treatment in ICC‐TRCs, and consistently, LCA lectin enrichment followed by IB showed reduced PSGL1 after SFT treatment (**Figure** **8**A). Additionally, LCA staining of tumor tissues showed that SFT decreased the level of core fucosylation in ICC (Figure S27, Supporting Information). Subsequently, we used a core fucosylation inhibitor named 2F‐Peracetyl‐Fucose (2‐FF) to treat ICC‐TRCs. Our data showed that 2‐FF treatment significantly inhibited the growth of colony spheroids, migration, and invasion, and decreased F‐actin fluorescence intensity and F/G‐actin ratio in ICC‐TRCs (Figure 8B,E). The action model of SFT against ICC‐TRCs was shown in **Figure** **9**.

## 3 Discussion

As a synthetic retinoid, SFT displays outstanding anticancer activities against multiple cancer cell lines in vitro, including HCC, melanoma, lung cancer, ovarian cancer, breast cancer as well gastric cancer.^[^ ^11^, ^12^, ^13^, ^14^ ^]^ It also inhibits the growth of xenograft tumor and PDX model, and decreases lung metastasis of TRCs in vivo.^[^ ^12^, ^13^ ^]^ In the current studies, we provide experimental evidences for the excellent anticancer activity of SFT against ICC‐TRCs in vitro and in vivo, and illuminate the underlying mechanisms. For one thing, SFT induces the translocation of RARɑ from the cytoplasm to the nucleus, leading to a decreased transcriptional activity of *SELP* as well a down‐regulation of P‐selectin. For another, SFT inhibits the enzymatic activity of FUT8, resulting in a decrease of core fucosylation in PSGL1. These changes disturb the regulation of PSGL1 on cytoskeleton and lead to the death of ICC‐TRCs.

Innovative drugs are reshaping oncotherapy modalities and improving the overall patient survival.^[^ ^32^, ^33^ ^]^ Targeting retinoic acid or its receptors is a clinical available strategy to explore innovative drugs for overcoming the stemness of cancers. A well‐known case is a clinical success of the application of ATRA on acute promyelocytic leukemia (APL). ATRA effectively induces the differentiation of pathological immature cells, achieving the purpose of treating APL. This success deepens our understanding of the role of the RARα pathway played in normal hematopoiesis and leukemogenesis, and influences a generation of drug development.^[^ ^34^ ^]^ Nevertheless, the broader extended application of ATRA beyond APL have largely gone unfulfilled, primarily due to the body's rigid regulation of retinoid homeostasis and the heterogeneity of cancers, of which only a subset is susceptible to retinoids.^[^ ^35^ ^]^ Breakthroughs in the understanding of retinoid metabolism as well as the revolution in cancer genetics promote the development of synthetic retinoids with more activity, less toxicity, and higher water‐solubility.^[^ ^34^ ^]^ These structure optimizations are mainly achieved by the modification of hydrophobic units, polar terminus or link units of ATRA.^[^ ^35^ ^]^ For instance, a substitution of polar end acid groups by aryl carboxylic acid does not affect the efficacy of ATRA, and a replacement of benzoic acid with aromatic ring improves the water‐solubility.^[^ ^36^, ^37^, ^38^ ^]^ SFT is a novel synthetic retinoid from previously constructed synthetic retinoic acid compound library. Structurally, it is a racemic sulfoxide derivative of 5‐pyrimidine acid skeleton, with a significantly increased water‐solubility.^[^ ^11^ ^]^ Our previous studies indicated that SFT targets RARɑ/SOS2/RAS signal axis and thereby overcomes stemness as well as sorafenib resistance in HCC.^[^ ^12^ ^]^ In melanoma, it induces RARγ translocation from the nucleus to the cytoplasm, leading to the downloading of Cdc42, which decreases F‐actin and inhibits cytoskeletal tension, and causes death of TRCs.^[^ ^13^ ^]^ In gastric cancer, SFT exerts antitumor effects by down‐regulating the expression of WNT4 via RARα.^[^ ^14^ ^]^ These studies implied that SFT is a promising drug targeting TRCs. Our works evaluates the efficacy of SFT against more aggressive and lethal cancer type, and found it also exhibits a robust inhibition against ICC‐TRCs in vitro and in vivo. It shows that SFT may be a promising drug for breaking the clinic predicament of ICC.

RARs belonging to nuclear receptor superfamily are important transcription factors targeting diverse human genes. Previous studies demonstrated that SFT alters the intracellular localization of RARɑ or RARγ in TRCs of HCC or melanoma, respectively,^[^ ^12^, ^13^ ^]^ which may be explained by the heterogeneity between different tumor types. Our studies find that SFT can not alter the expression of RARɑ but increase the nuclear translocation of RARɑ in ICC‐TRCs. The results of RNA‐seq, qRT‐PCR, IB, dual luciferase reporter assay and ChIP‐PCR indicated that RARɑ negatively regulates the transcription of *SELP*. SFT transcriptionally inhibited the expression of P‐selectin via triggering RARɑ nuclear translocation. We found that P‐selectin is a oncogene for maintaining the stemness of ICC‐TRCs. Previous studies indicated that P‐selectin works in a physical manner, which helps cancer cells roll in the vascular and thereby promotes metastasis of cancers.^[^ ^21^ ^]^ As our understanding improves, an increasing number of studies are focusing on the role of P‐selectin in cancers beyond its physical functions. For instance, P‐selectin mediates the proliferation and invasion of glioblastoma by altering the activation state of microglia/macrophages.^[^ ^24^ ^]^ Platelets‐derived P‐selectin promotes colorectal cancer growth and metastasis by activating PSGL‐1/JNK/STAT1 signaling pathway, which promotes C5 transcription and activates the C5a/C5aR1 axis in tumor associated macrophages.^[^ ^39^ ^]^ We found that SFT treatment or P‐selectin intervention significantly destroyed the cytoskeleton of ICC‐TRCs. Stable cytoskeleton is served as a crucial structural foundation for cell's survival and activity, including cancer cells and CSCs, and thus targeting cytoskeleton is considered as a promising strategy for tumor treatment.^[^ ^26^ ^]^ To this end, several drugs were explored to directly act on some components of cytoskeleton, such as actin inhibitors (SMIFH2 and MBQ‐167), Fascin inhibitors (NP‐G2‐044 and BDP‐13176), Kinesin inhibitors (Ispinesib and SB‐743921), etc.,^[^ ^26^ ^]^ some of which have entered clinical trials (NCT04863950, NCT00089973, NCT00136513), indicating its potentiality for the development of antitumor drugs. P‐selectin has no direct connection to cytoskeleton, yet its ligand PSGL1 directly binds to cytoskeleton via ERM complex.^[^ ^25^ ^]^ Several studies demonstrated the regulation of PSGL1 on cytoskeleton via ERM complex.^[^ ^22^, ^40^, ^41^ ^]^ Accordingly, we infer that SFT can destroy PSGL1‐mediated stable cytoskeleton via transcriptionally inhibiting P‐selectin by promoting nuclear translocation of RARɑ in ICC‐TRCs.

Of note, SFT suppresses the glycosyltransferase activity of FUT8 in ICC‐TRCs. FUT8 is the only fucosyltransferase involved in core fucosylation, which transfers GDP fucose to the initial GlcNAc residue of the N‐glycan core by forming α‐1,6 glycosidic bonds, constituting the core fucose.^[^ ^31^, ^42^ ^]^ This unique biological significance is determined by the specific structure of FUT8, including a catalytic domain, an N‐terminal α‐helical domain and a C‐terminal Src homology 3 (SH3) domain.^[^ ^31^ ^]^ Compared to other glycosyltransferases, SH3 domain is the exclusive structure of FUT8, regulating its localization and core fucosylation, in which the HIS535 is the key residue.^[^ ^43^ ^]^ In addition, the residues of ARG365, GLU273, and LYS369 play crucial roles in the catalytic activity of FUT8.^[^ ^44^ ^]^ It promotes tumorigenesis and progression via altering the glycosylation pattern of key proteins, and up‐regulates in various cancer types, including liver cancer, colorectal cancer, ovarian cancer, prostate cancer, lung cancer, breast cancer, thyroid cancer, pancreatic cancer, malignant melanoma.^[^ ^45^, ^46^, ^47^, ^48^ ^]^ FUT8 also up‐regulates in the CSCs of breast cancer, esophageal cancer, and pancreatic cancer.^[^ ^49^, ^50^, ^51^ ^]^ The exciting potential to exploit FUT8 therapeutically becomes an important focal point of the oncotherapy field. We found that SFT inhibits the glycosyltransferase activity of FUT8 via directly binding to its SER245, ASN247, and ILE242 residues according to the results from Lip‐SMap, molecular docking assay and MST. These amino acids are not key sites for FUT8 enzyme activity, so we inferred that SFT may alter its spatial conformation by binding to FUT8 (data not shown), thereby affecting its glycosyltransferase activity.

PSGL1 is a glycoprotein with N‐glycosylation and O‐glycosylation. Previous studies indicated that O‐glycosylation is involved in the major functions of PSGL1, including its regulation on cell migration and adhesion.^[^ ^52^ ^]^ The predominate sialylated fucosylated O‐linked glycan, sialyl Lewis x (sLex) in O‐glycosylation of PSGL1 is necessary for P‐selectin to recognize and bind to it, and this specific O‐polysaccharide structure is also considered as the functional core of PSGL1.^[^ ^53^ ^]^ Therefore, more researches focus on the regulatory mechanism of O‐glycosylation in PSGL1. Of note, β (1,6)—N‐acetylglucosamine transferase I (C2GnT‐I) and fucosyltransferase 7 (FucT VII) can catalyze the N‐glycosylation of PSGL1. In the presence of core 2 modified PSGL1, the N‐glycan formed by C2GnT‐I catalysis plays an important role in the binding of P‐selectin and PSGL1.^[^ ^54^ ^]^ We first found the presence of core fucosylation in PSGL1, and this glycosylation pattern helps regulate the stability of cytoskeleton in ICC‐TRCs. Inhibiting core fucosylation of PSGL1 significantly destroys the cytoskeleton and thus causes cell death in ICC‐TRCs. These studies suggested that suppression of glycosylation is also a promising strategy for overcoming stemness in ICC.

In conclusions, our findings reveal a novel role of P‐selectin/PSGL1 axis which promotes stemness via remolding cytoskeleton in ICC. We also found a novel mode of action via which sulfarotene selectively inhibits ICC‐TRCs. These findings expand the potential application of SFT in cancers. Building on our project's previous study,^[^ ^11^, ^12^, ^13^ ^]^ in the future it will be interesting to see if sulfarotene can become an effective drug to overcome stemness of cancers in clinical setting.

## 4 Experimental Section

### Cell Culture and Reagents

Human ICC cell lines RBE (TCHu179) and HCCC9810 (TCHu 17) were purchased from National Collection of Authenticated Cell Cultures (Shanghai, China). Human ICC cell line HUCCT1 and mouse ICC cell line AY‐LTC2 were maintained in Liver Cancer Institute, Zhongshan Hospital, Fudan University (Shanghai, China). Human extrahepatic cholangiocarcinoma cell line TFK1 was kindly given by Eastern Hepatobiliary Surgery Hospital (Shanghai, China). All cells were cultured in RPMI‐1640 medium (11 875 500, Gibco, New York) containing 10% fetal bovine serum (FBS; 10270‐106, Gibco) and 1% penicillin‐streptomycin (1 719 675, Gibco), being kept in a humidified ThermoForma incubator (Thermo Fisher Scientific, Waltham, MA) with the condition of 37 °C and 5% CO_2_. CDDP (HY‐17394), 5‐FU (HY‐90006), GEM (HY‐17026), PEM (HY‐109099), KF38789 (HY‐103358), TM (HY‐A0098), TG (HY‐12588), 2‐FF (HY‐W096600) were purchased from MedChemExpress (New Jersey, USA). Recombinant PNGase F (P0704S) and recombinant O‐glycosidase (P0733) were from New England BioLabs (MA, USA). Salmon fibrinogen (SEA‐133) and thrombin (SEA‐135) were purchased from Sea Run Holdings Inc. (Freeport, ME, USA).

### ICC‐TRCs Sphere Formation, Growth, Migration, and Invasion Assay

ICC‐TRCs were selected using 3D fibrin gel, and the detail experimental procedure was described in the previous studies.^[^ ^7^, ^8^, ^9^, ^10^, ^11^, ^12^, ^13^, ^14^, ^15^, ^16^ ^]^ The sphere formation of ICC‐TRCs was observed using inverted microscope (Olympus, Tokyo, Japan). After treated with drugs for 48 h, the growth of ICC‐TRCs was detected using Cell Counting Kit‐8 (CCK‐8, 40203ES80, Yeasen, Shanghai, China) according to the manufacturer's instructions. The optical density (OD) values of generated formazan were measured at 450 nm by FlexStation 3 Multi‐Mode Microplate Reader (Molecular Devices Corporation, Sunnyvale, CA, USA). The inhibition rate (%) was calculated based on the formula [(OD_control_ – OD_treatment_)/(OD_control –_ OD_blank_)] × 100. The migration and invasion ability of ICC‐TRCs was evaluated using Transwell assay as previous described.^[^ ^7^, ^16^ ^]^

### Xenograft Tumor Models

Male BALB/c nude mice (4–6 weeks old) and C57BL/6 mice (4–6 weeks old) were purchased from Beijing Vital River Laboratory Animal Technology Co., Ltd. (Beijing, China). HUCCT1‐TRCs or HUCCT1 were 1:1 mixed with matrix and then subcutaneously injected into the right scapula of BALB/c nude mice. When the tumor grew to 50 mm^3^, different treatments (solvent, SFT 0.68, SFT 6.8, GEM 15, PEM 10, and KF38789 10 mg kg^−1^) were administrated to mice. As regards tumor‐bearing C57BL/6 mouse model, additionally, a total of 5 × 10^6^ AY‐LTC2‐TRCs were subcutaneously injected per mouse. After the tumor grew to 100 mm^3^, different treatments (solvent, SFT 0.68, SFT 6.8, WYC‐209A 6.8, WYC‐209B 6.8 mg kg^−1^,) were administrated to mice. The longest diameter (L) and shortest diameter (W) of tumor as well the body weight of mice was detected twice 1 week or every two days. The volume of tumor was calculated as followed: 0.5 × L × W^2^. At the end of experiments, mice were euthanized and the bore tumor was recorded, separated, weighted, and saved as two parts: one part was in −80 °C, and another in formalin.

All procedures involving mice and experimental protocols were approved by Institutional Animal Care and Use Committee of the First Affiliated Hospital of Wenzhou Medical University (WYYY‐AEC‐2023‐083 and WYYY‐AEC‐2024‐186).

### IB and CoIP

For IB, the total proteins and nuclear proteins were extracted using RIPA lysis buffer (P0013B, Beyotime, Nanjing, China) and nuclear and cytoplasmic protein extraction kit (P0028, Beyotime), respectively. Then the proteins were separated by 10% or 12% SDS‐PAGE and followed transferred to polyvinylidene fluoride (PVDF) membrane. After blocked with 5% nonfat milk for 90 min, the membrane was probed with primary antibodies (Table S6, Supporting Information) at 4 °C overnight and related secondary antibodies (Table S6, Supporting Information) at room temperature for 1 h. The protein bands were visualized under Tanon 5200 Automatic Chemiluminescence Image Analysis System (Tanon, Shanghai, China) using Chemistar highsig ECL western blotting substrate (180‐5001, Tanon). For CoIP, cells were collected and lysed in NP‐40 lysis buffer (P0013F, Beyotime). After pre‐treated with protein A/G magnetic beads (HY‐K0202, MedChemExpress) for 1 h at 4 °C, the cell lysates were used for IP with the indicated antibodies. In brief, related antibody was mixed with about 1 mg of cell lysate, and rotated slowly 4 °C for overnight. Then, the mixture was mixed with protein A/G magnetic beads and incubated at room temperature for 1 h. After washed five times, the samples were subjected to SDS‐PAGE electrophoresis.

### Lectin Blotting and Lectin Enrichment Assay

For lectin blotting, samples were dissociated by SDS‐PAGE and transferred to PVDF membranes. The membranes were probed with 5 µg mL^−1^ of LCA (B‐1045, Vector laboratories, CA) at 4 °C overnight, and incubated with horseradish peroxidase streptavidin (SA‐5004, Vector) at room temperature for 1 h. For lectin enrichment assay, 50 µL of LCA was added into cell lysate (about 1 mg) and incubated with rotation at 4 °C overnight. Then, 50 µL of agarose streptavidin (SA‐5010, Vector laboratories) was mixed, and incubation for 4 h at 4 °C. The mixture was washed and centrifugated, and the precipitation was extracted with SDS‐PAGE sample loading buffer at 100 °C for 10 min. The samples were subjected to IB and lectin blotting.

### IF

Pre‐treated ICC‐TRCs were fixed with 4% paraformaldehyde for 15 min. After fixation, ICC‐TRCs were treated with 0.5% Triton X‐100/PBS (P0096, Beyotime) for 10 min. Then, ICC‐TRCs were blocked with 5% bovine serum albumin (BSA)/PBS at room temperature for 30 min and then incubated with anti‐Ki67, cleaved‐caspase3, or RARɑ antibodies (1:200 in 5% BSA/PBS) at 4 °C overnight. After washed, ICC‐TRCs were incubated with related fluorescent‐labeled secondary antibodies (1:200 in 5% BSA/PBS) at room temperature for 1 h. Nuclear was stained with DAPI (C1005, Beyotime) at room temperature for 5 min. Finally, samples were observed on a laser confocal microscope (NIKON ECLIPSE TI, NIKON, Tokyo, Japan) with an image system of NIKON C2. All used antibodies were displayed in Table S6 (Supporting Information).

### IHC

Paraffin‐embedded tissue blocks were cut into 5 mm slices. The slices were deparaffinized with xylene and rehydrated with graded ethanol series. After removing endogenous enzymes with 3% methanol/H_2_O_2_ and blocked with 5% BSA/PBS, slices were incubated with anti‐Ki67, anti‐cleaved‐caspase3, anti‐RARɑ antibody, or anti‐SELP antibody (1:200 in 5% BSA/PBS) at 4 °C overnight. After washed, slices were treated with appropriate secondary antibodies at room temperature for 1 h. Then, the staining was completed using 3,3'‐diaminobenzidine tetrahydrochloride (DAB) kit (P0202, Beyotime) and the nuclear was stained using hematoxylin (C0105S, Beyotime) according to the manufacturer's manual. All slices were observed on a SLIDEVIEW VS200 (Olympus, Tokyo, Japan). Abovementioned antibodies were displayed in Table S6 (Supporting Information).

Semiquantitative analysis of IHC data was conducted using Image J soft. For Ki67, positive cell percentage was calculated for statistic analysis. For other indicators, the IHC score was calculated as followed

(1) IHC=Intensityscore×Positivecellscore

Intensity score was defined as follows: 0, negative; 1, weak; 2, moderate; 3, strong. Positive cell score was defined as follows: 0, < 5%; 1, 5%–25%; 2, 25%–50%; 3, 50%–75%; 4, > 75%. Five random visual fields were selected from each sample and the mean was served as the IHC score of one sample.

### qRT‐PCR

The total RNA of cells or tissues was exacted using RNA‐Quick Purification Kit (RN001, ES Science, Shanghai, China). After reverse transcription with Hifair V one‐step RT‐gDNA digestion SuperMix for qPCR (11141ES60, Yeasen), the PCR process was achieved using Hieff qPCR SYBR Green Master Mix (11202ES08, Yeasen) on a QuantStudio5 fluorescence quantitative PCR system (Applied Biosystems, Foster City, CA, USA). The conditions were set as follows: initial denaturation at 95 °C for 5 min, 40 cycles of 95 °C for 10 s, and 60 °C for 30s. The sequences of all primers were showed in Table S7 (Supporting Information).

### RNA Interference and Plasmid Transfection

To interfere the expression of genes, cells were transfected with siRNA by applying riboFECT CP (C10511‐05, RIBOBIO, Guangdong, China) according to the manufacturer's manual. The targeted sequence of siRNA was showed in Table S8 (Supporting Information).

To overexpress genes, human SELP plasmid (PGMLV‐CMV‐H_SELP‐3×Flag‐PGK‐Puro), human wild‐type FUT8 plasmid (lenti‐CMV‐H_FUT8‐3×Flag‐PGK‐Neo, ACACTCATCTTGGAATCTCAGAATTGGCGC), and mutant FUT8 plasmid (lenti‐CMV‐H_FUT8(MT)‐HA‐PGK‐Neo, GCCGCAGCAGCGGCAGCCGCGGCCGCAGCG) were constructed by Genomeditech (Shanghai, China). The plasmid was transfected into cells using Hieff Trans Liposomal Transfection Reagent (40802ES02, Yeasen) according to the manufacturer's instructions.

### Molecular Docking Assay

For predicting the binding site between SFT and target proteins, the structure file of SFT using ChemDraw 20.0, input it into Chem 3D 20.0 software and adjusted it to a 3D structure was prepared. Molecular mechanics (MM2) force field was used to minimize energy of SFT. It was output as a pdb format file. Then, the 3D model structure of proteins (RARɑ, UniProt ID: P10276; FUT8, 2DE0) was downloaded from PDB database (https://www.rcsb.org/). The molecular docking was conducted using AutoDock Vina 1.1.2^[^ ^55^ ^]^ and visualized using Discovery Studio 2018. For predicting the binding site between RARɑ and the transcriptional domain of *SELP*, the DNA 3D structure of *SELP* was constructed using HDOCK Server.^[^ ^56^ ^]^ Then the hybrid docking strategy was used to predict the binding site between RARɑ and the transcriptional domain of *SELP*.

### Expression and Purification of Recombinant Proteins

At first, the target gene was linked to pSumo‐Mut vector. To express target proteins, the recombinant plasmid was transformed into competent cells of *Escherichia coli* (Arctic Express) and grown in a Luria–Bertani (LB) medium with kanamycin (50 µg mL^−1^) or ampicillin (100 µg mL^−1^) with a shaking of 200 rpm at 37 °C. When the OD value at 600 nm reaching to 0.6–0.8, isopropyl β‐D‐thiogalactoside (ITPG, 367‐93‐1, Sigma, St. Louis, MO, USA) was added, and the recombinant cells were cultivated with a shaking of 200 rpm at 15 °C overnight to induce the protein expression. The recombinant cells were harvested by centrifugation at 4000 rpm for 15 min at 4 °C. Next, the denaturation of the inclusion body protein was performed. After ultrasonication (power: 400 W, working time: 4 s, intermittent time: 8 s, total time: 20 min), cell lysate was centrifugated at 10 000 rpm for 20 min at 4 °C and the precipitation was washed using inclusion body washing buffer (20 mm Tris, 1 mm EDTA, 2 m urea, 1 m NaCl, 1% Triton X‐100, pH 8.0) three times. The precipitation was dissolved using lysis buffer (20 mm Tris, 5 mm DTT, 0.15 m NaCl, 8 m urea, pH 8.0) and incubated at 4 °C overnight. After centrifugation at 10 000 rpm for 15 min at room temperature, the supernatant was gradually diluted in multiple gradients of 20 mm Tris‐HCl, 0.15 m NaCl, pH 8.0 buffer and stirred slowly, and then put the protein solution into a dialysis bag and dialyzed overnight in a buffer (20 mm Tris HCl, 0.15 m NaCl, pH 8.0). Finally, to purify the proteins, the supernatant solution was loaded at a flow rate of 0.5 mL min^−1^ onto a Ni‐IDA‐Sepharose Cl‐6B affinity chromatography column. Then, the column was washed with Ni‐IDA binding buffer at a flow rate of 0.5 mL min^−1^ until the effluent's OD_280_ value reaching the baseline, and followed with Ni‐IDA washing buffer (20 mm Tris HCl, 20 mm imidazole, 0.15 m NaCl, 8 m urea, pH 8.0) at a flow rate of 1 mL min^−1^ until the effluent's OD_280_ value reaching the baseline. The bound proteins were eluted with Ni‐IDA elution buffer (20 mm Tris HCl, 250 m imidazole, 0.15 m NaCl, 8m urea, pH 8.0) at a flow rate of 1 mL min^−1^.

### MST Assay

To evaluate the binding affinity between SFT and target proteins, an MST assay was conducted by Monolith NT Automated (NanoTemper Technologies). In briefly, the purified recombinant protein was labeled with RED‐tris‐NTA 2nd generation dye solution (MO‐L018, NanoTemper Technologies) according to the manufacturer's manual. Then, 50 nm labeled proteins were mixed with different concentrations of SFT by pipetting up and down several times. The interaction of labeled proteins and SFT was measured at 20% LED and High MST power. The data were collected by the MO.Control Software that further determined the *K*_d_ values.

### RNA‐seq and Related Bioinformatics Analysis

After HUCCT1‐TRCs or HUCCT1 cells were treated with SFT for 48 h, total RNA was isolated using TRIzol reagent (15 596 026, Invitrogen, CA, USA). The quantity and purity of RNA was monitored using Nanodrop2000 (NanoDrop, Wilmington, DE, USA). The mRNA was separated from total RNA using oligo dT magnetic beads (25‐61005, Thermo Fisher, CA, USA) and subsequently fragmented using fragmentation buffer. Then, the fragmented mRNA was reversely transcripted to cDNA. Then the cDNA was subjected to end‐repair, phosphorylation and “A” base addition according to Illumina's library construction protocol. Libraries were size‐selected for cDNA target fragments of 300 bp on 2% Low Range Ultra Agarose followed by PCR amplified using Phusion DNA polymerase (NEB) for 15 PCR cycles. After quantified by Qubit 4.0, paired‐end RNA‐seq library was sequenced with the NovaSeq 6000 sequencer (Shanghai Majorbio Bio‐pharm Biotechnology Co., Ltd., Shanghai, China).

Critical genes responsive for SFT treatment were identified and ranked based on an integrated analysis of RNA‐seq. In detail, the expression level of each transcript was calculated according to the transcripts per million reads (TPM) method. The edgeR package of R (version 4.2.0)^[^ ^57^ ^]^ was employed to analyze the DEGs between two different groups, setting the threshold as |logFC| > 1 and *p* value < 0.05. The pheatmap package of R was used to analyze the trend of genes with SFT concentration changes. The KEGG enrichment analysis was conducted using the clusterProfiler package of R.^[^ ^58^ ^]^ In addition, Cytoscape soft^[^ ^59^ ^]^ was applied to perform the MCODE algorithm aiming to select hub genes, setting the threshold as node score cutoff of 0.2 and K‐core of 2. And the network centrality analysis, including betweenness centrality, degree centrality, and closeness centrality, was performed using Cytoscape soft.

### Dual Luciferase Reporter Assay

The plasmids for RARα overexpression (pCDNA3.1(+)‐H_RARA), RARα control (pCDNA3.1(+)‐H_RARA‐NC), and human wild‐type SELP (PGL3‐basic‐H_SELP promoter(−1941 to +50)^WT^(GGGTCTCGGGCAGTTTA, TTCCTTTCACATGACCTA)‐luciferase) and mutant SELP (PGL3‐basic‐H_SELP promoter(‐1941 to +50)^MU^(TTTGAGATTTACTGGGC, GGAAGGGACACGTCAAGC)‐luciferase) were constructed by Genomeditech. Plasmids were transiently transfected into ICC‐TRCs. After cultured for 48 h, the luciferase activities were detected by a dual luciferase reporter gene assay kit (RG027, Beyotime) according to manufacturer's manual.

### ChIP‐PCR

After treated with SFT for 48 h, HUCCT1‐TRCs were crosslinked in 1% formaldehyde solution at room temperature for 10 min and quenched with 125 mm glycine. The chromatin was exacted from cross‐linked cells by adding lysis buffer and further fragmented using sonicate. The chromatin segments were immunoprecipitated with 10 µL anti‐RARα or control IgG. The protein‐DNA complexes were eluted and separated. Protein samples were used to IB for quality control. Additionally, two primers (*SELP* prime A and B) were designed for the promoter region of *SELP*. Accordingly, PCR was performed to quantify the DNA samples.

### F/G‐actin Ratio Detection Assay

G‐actin and F‐actin were separated using the G‐actin/F‐actin in vivo assay kit (#BK037, Cytoskeleton Inc., Denver, CO, USA) according to the manufacturer's instructions. In brief, cells or tissues were lysed in prewarmed lysis and F‐actin stabilizing buffer supplemented with 1% protease inhibitor and 1% ATP. For tissues, 25G syringe needle was used to homogenize samples. After incubated at 37 °C for 10 min, the lysate was centrifuged at 350 × g for 5 min at room temperature. The supernatant (300 µL) was centrifuged at 100 000 × g for 1 h at 37 °C to pellet F‐actin with G‐actin leaving in the supernatant. The pellet was resuspended with 300 µL of F‐actin destabilizing buffer on ice for 1 h with pipetting up and down every 15 min. Isochoric G‐actin and F‐actin fractions were mixed with 5 × SDS sample buffer and subjected to IB. The data analysis was conducted using Image J soft.

### Phalloidin Staining

Phalloidin selectively binds to the F‐actin of eukaryotes, so it was used to detect the level of F‐actin in ICC‐TRCs. Pre‐treated ICC‐TRCs were fixed, permeabilized, and then incubated with phalloidin staining kit (G1041, Servicebio technology CO., LTD, Wuhan, China) for 2 h at room temperature. After the nuclear stained with DAPI, the cells were observed with a laser confocal microscope.

### Glycosylation Analysis in ICC‐TRCs

To confirm glycosylation patterns of PSGL1 in ICC‐TRCs, cell lysates were treated with recombinant PNGase F and O‐glycosidase. Briefly, the cell lysates were mixed with glycoprotein denaturing buffer at 100 °C for 10 min. The mixture was added with NP‐40, glycobuffer, and recombinant glycosidase (Neuraminidase was necessary for O‐glycosidase) at 37 °C for 1 h, then the reaction was stopped by 5 × SDS sample buffer, which was next used for IB.

### Lip‐SMap

Lip‐SMap is a high‐throughput method to identify the potential targets of small molecular drugs.^[^ ^30^ ^]^ In detail, proteins (1 mg) were exacted from HUCCT1‐TRCs using Native cell lysate (20 mm HEPES, 155 mm NaCl, 1 mm MgCl_2_, 1 mm KH_2_PO_4_, 3 mm Na_2_HPO_4_, pH 7.5). The cell lysate was homogeneously mixed using a 25G syringe needle, incubated with ice bath for 20 min, and centrifuged with 16 000 × g at 4 °C for 5 min. The supernatant was incubated with DMSO or SFT (1 mm) at 25 °C for 10 min. Proteinase K (5 µg, Sigma‐Aldrich) was added to each sample for the following 4 min, and the reaction was immediately stopped by heating at 98 °C for 3 min. Sodium deoxycholate was next added to the samples. For quality control, 20 µL of samples were assessed using SDS‐PAGE electrophoresis. Complete digestion was conducted with trypsin at a ratio of 1:50 (trypsin:protein) for 16 h at 37 °C, and then the reaction was ended by formic acid (pH < 2). Subsequently, a MS analysis was performed on these samples by National Facility for Protein Science in Shanghai (NFPS). The MS data were compared to a UniProt human database^[^ ^60^ ^]^ by Andromeda algorithm built‐in MaxQuant engine. LFQ algorithm was used for peptide quantification with a delayed normalization method to normalize peptide intensity quantification across all the samples. The limma package of R^[^ ^61^ ^]^ was employed to analyzed the differential peptides between DMSO and SFT group.

### Differential Gene Analysis and Survival Analysis in Public ICC Data

RNA‐seq data of human ICC CHOL and GSE107943 was downloaded from TCGA and GEO database.^[^ ^62^, ^63^ ^]^ After the raw data transformed into log_2_(TPM + 1), the DGEs were analyzed using limma package of R.^[^ ^61^ ^]^ The Survival package of R was used to calculate the optimal cutoff value and perform survival analysis.

### CRISPR/Cas9 Mediated Gene Knockout

To establish FUT8‐knockout HUCCT1, the specific sgRNA targeting human FUT8 (ATCTGACAGAACTGGTTCAG) and Cas9 plasmid were purchased from Genomeditech. Both the FUT8 sgRNA and Cas9 plasmid were packaged with lentivirus virus. At first, HUCCT1 cells were infected with lentiviral particles carrying Cas9 and blasticidin resistance (MOI = 100) for 16 h and then replaced with complete media for a 48 h cultivation. The cells were treated with blasticidin (30 µg mL^−1^) for 72 h, and this step was repeated again to obtain stable cells. Next, the cells were infected with lentiviral particles carrying FUT8 sgRNA and puromycin resistance (MOI = 100), and stable cells were subsequently selected by puromycin for 72 h. Then FUT8‐knockout clones were isolated by single‐cell dilution cloning from the positive polyclonal sgRNA‐transduced populations, which were further identified by IB. LentiCRISPR plasmid was used as a negative control.

### Caspase3 Activity Detection

The caspase3 activity was detected in DMSO or different drugs treated ICC‐TRCs or 2D ICC cells using caspase3 activity assay kit (C1116, Beyotime) according to the manufacturer's instructions.

### Statistics

Statistics analyses and plotting were performed by R (version 4.2.0) and Graphpad Prism 8.0 software. For experimental data, *t‐*test was used to analyze the difference between two groups, while Tukey's multiple comparisons test was performed to achieve multiple comparisons within a set of experiments. All data were expressed as mean  ± standard deviation (SD) and significance was defined as *p* < 0.05 unless otherwise mentioned.

## Conflict of Interest

The authors declare no conflict of interest.

## Author Contributions

Dr. Xiaojing Du, Miss Zhuoran Qi, Miss Sinuo Chen, and Miss Jinlan Wu made equal contributions to the research work.

[Correction added on 12 December 2024 after online publication: Author Contributions is added.]

## Supporting information



Supporting Information

## Data Availability

The data that support the findings of this study are available from the corresponding author upon reasonable request.

## References

[1] D. Moris , M. Palta , C. Kim , P. J. Allen , M. A. Morse , M. E. Lidsky , Cancer J. Clin. 2023, 73, 198. 10.3322/caac.21759 36260350

[2] Z. J. Brown , S. M. Ruff , T. M. Pawlik , Expert Rev. Anticancer Ther. 2023, 23, 257. 36744395 10.1080/14737140.2023.2176846

[3] Y. Liu , X. Liang , W. Dong , Y. Fang , J. Lv , T. Zhang , R. Fiskesund , J. Xie , J. Liu , X. Yin , X. Jin , D. Chen , K. Tang , J. Ma , H. Zhang , J. Yu , J. Yan , H. Liang , S. Mo , F. Cheng , Y. Zhou , H. Zhang , J. Wang , J. Li , Y. Chen , B. Cui , Z. W. Hu , X. Cao , F. Xiao‐Feng Qin , B. Huang , Cancer Cell 2018, 33, 480. 29533786 10.1016/j.ccell.2018.02.005

[4] H. Li , Y. Zhou , M. Wang , H. Wang , Y. Zhang , R. Peng , R. Zhang , M. Zhang , M. Zhang , P. Qiu , L. Liu , Q. Zhao , J. Liu , Cancer Sci. 2021, 112, 4593. 34449943 10.1111/cas.15120 PMC8586666

[5] Y. Li , Y. Song , P. Li , M. Li , H. Wang , T. Xu , X. Yu , Y. Yu , Y. Tai , P. Chen , X. Cai , X. Wang , L. Xiang , R. Deng , X. Zhang , L. Gao , X. Wang , J. Liu , F. Cao , J. Immunother. Cancer 2020, 8, e000111. 32152220 10.1136/jitc-2019-000111 PMC7061898

[6] J. Li , Y. Liu , H. Jiang , T. Wang , K. Li , X. Lao , G. Liao , Y. Liang , Transl. Oncol. 2023, 33, 101681.37137218

[7] X. Du , X. Zhang , Z. Qi , Z. Zeng , Y. Xu , Z. Yu , X. Cao , J. Xia , Comput. Struct. Biotechnol. J. 2023, 21, 5174. 37920816 10.1016/j.csbj.2023.09.020 PMC10618119

[8] X. Du , Z. Qi , J. Xu , M. Guo , X. Zhang , Z. Yu , X. Cao , J. Xia , Mol. Oncol. 2022, 16, 3703. 36062307 10.1002/1878-0261.13305 PMC9580891

[9] Z. Li , Z. Xu , W. Chen , X. Du , C. Ou , Z. Luo , R. Wang , C. Zhang , C. Ge , M. Han , F. Wang , R. He , W. Sun , J. Ma , X. Liang , Z. Liu , Nat. Chem. Biol. 2024, 20, 1341. 38720107 10.1038/s41589-024-01612-6 PMC11427348

[10] J. Xu , L. Zhou , X. Du , Z. Qi , S. Chen , J. Zhang , X. Cao , J. Xia , Metabolites 2023, 13, 1132. 37999228 10.3390/metabo13111132 PMC10673379

[11] J. Chen , X. Cao , Q. An , Y. Zhang , K. Li , W. Yao , F. Shi , Y. Pan , Q. Jia , W. Zhou , F. Yang , F. Wei , N. Wang , B. Yu , Nat. Commun. 2018, 9, 1406. 29643385 10.1038/s41467-018-03877-7 PMC5895803

[12] F. Qi , W. Qin , Y. Zhang , Y. Luo , B. Niu , Q. An , B. Yang , K. Shi , Z. Yu , J. Chen , X. Cao , J. Xia , J. Exp. Clin. Cancer Res. 2021, 40, 280. 34479623 10.1186/s13046-021-02085-4 PMC8418008

[13] Y. Zhang , Q. Dong , Q. An , C. Zhang , E. Mohagheghian , B. Niu , F. Qi , F. Wei , S. Chen , X. Chen , A. Wang , X. Cao , N. Wang , J. Chen , Adv. Sci. 2022, 9, e2203173. 10.1002/advs.202203173 PMC9631059 36031407

[14] Z. Qian , W. Lin , X. Cai , J. Wu , K. Ke , Z. Ye , F. Wu , Cancer Biol. Ther. 2024, 25, 2299288. 38178596 10.1080/15384047.2023.2299288 PMC10773637

[15] J. Liu , Y. Tan , H. Zhang , Y. Zhang , P. Xu , J. Chen , Y. C. Poh , K. Tang , N. Wang , B. Huang , Nat. Mater. 2012, 11, 734. 22751180 10.1038/nmat3361 PMC3405191

[16] X. Du , Z. Qi , Y. Jiao , W. Wu , Q. Huang , X. Sun , S. Hu , Cell Signal. 2024, 118, 111126. 38453126 10.1016/j.cellsig.2024.111126

[17] D. Chaker , C. Desterke , N. Moniaux , M. A. Bani , N. Oudrhiri , J. Faivre , A. G. Turhan , A. Bennaceur‐Griscelli , F. Griscelli , Stem Cells 2024, 42, 301 38262709 10.1093/stmcls/sxae006

[18] M. Moustafa , K. K. Dähling , A. Günther , L. Riebandt , D. J. Smit , K. Riecken , C. Schröder , R. Zhuang , T. Krech , M. Kriegs , B. Fehse , J. R. Izbicki , L. Fischer , B. Nashan , J. Li , M. Jücker , Cancers 2022, 14, 1882. 35454789 10.3390/cancers14081882 PMC9024696

[19] J. C. Lu , L. L. Wu , Y. N. Sun , X. Y. Huang , C. Gao , X. Y. Guo , H. Y. Zeng , X. D. Qu , Y. Chen , D. Wu , Y. Z. Pei , X. L. Meng , Y. M. Zheng , C. Liang , P. F. Zhang , J. B. Cai , Z. B. Ding , G. H. Yang , N. Ren , C. Huang , X. Y. Wang , Q. Gao , Q. M. Sun , Y. H. Shi , S. J. Qiu , A. W. Ke , G. M. Shi , J. Zhou , Y. D. Sun , J. Fan , Nat. Commun. 2024, 15, 621. 38245530 10.1038/s41467-024-44795-1 PMC10799889

[20] Y. Tan , X. Wang , H. Song , Y. Zhang , R. Zhang , S. Li , W. Jin , S. Chen , H. Fang , Z. Chen , K. Wang , Blood 2021, 137, 1503. 32854112 10.1182/blood.2020005698 PMC7976511

[21] H. Läubli , L. Borsig , Semin. Cancer Biol. 2010, 20, 169. 20452433 10.1016/j.semcancer.2010.04.005

[22] B. Huang , Y. Ling , J. Lin , X. Du , Y. Fang , J. Wu , Protein Cell. 2017, 8, 103. 28097631 10.1007/s13238-016-0364-4 PMC5291781

[23] R. Nolo , S. Herbrich , A. Rao , P. Zweidler‐McKay , S. Kannan , V. Gopalakrishnan , Oncotarget 2017, 8, 86657. 29156825 10.18632/oncotarget.21364 PMC5689715

[24] E. Yeini , P. Ofek , S. Pozzi , N. Albeck , D. Ben‐Shushan , G. Tiram , S. Golan , R. Kleiner , R. Sheinin , S. Israeli Dangoor , S. Reich‐Zeliger , R. Grossman , Z. Ram , H. Brem , T. M. Hyde , P. Magod , Nat. Commun. 2021, 12, 1912. 33771989 10.1038/s41467-021-22186-0 PMC7997963

[25] S. D. Zaongo , Y. Liu , V. Harypursat , F. Song , H. Xia , P. Ma , Y. Chen , Front. Immunol. 2021, 12, 710121. 34434194 10.3389/fimmu.2021.710121 PMC8380821

[26] Y. Li , D. Wang , H. Ge , C. Güngör , X. Gong , Y. Chen , Pharmaceuticals 2022, 15, 1369. 36355541 10.3390/ph15111369 PMC9698833

[27] S. Barkeer , S. Chugh , S. K. Batra , M. P. Ponnusamy , Neoplasia 2018, 20, 813. 30015157 10.1016/j.neo.2018.06.001 PMC6037882

[28] Q. Yang , T. Liu , T. Wu , T. Lei , Y. Li , X. Wang , Plant Physiol. 2022, 190, 340. 35789395 10.1093/plphys/kiac297 PMC9434254

[29] S. S. Pinho , C. A. Reis , J. Paredes , A. M. Magalhães , A. C. Ferreira , J. Figueiredo , X. G. Wen , F. Carneiro , F. Gärtner , R. Seruca , Hum. Mol. Genet. 2009, 18, 2599. 19403558 10.1093/hmg/ddp194

[30] S. Chen , X. Liu , C. Peng , C. Tan , H. Sun , H. Liu , Y. Zhang , P. Wu , C. Cui , C. Liu , D. Yang , Z. Li , J. Lu , J. Guan , X. Ke , R. Wang , X. Bo , X. Xu , J. Han , J. Liu , Cell Metab. 2021, 33, 565. 33657393 10.1016/j.cmet.2021.02.007

[31] Y. Huang , H. L. Zhang , Z. L. Li , T. Du , Y. H. Chen , Y. Wang , H. H. Ni , K. M. Zhang , J. Mai , B. X. Hu , J. H. Huang , L. H. Zhou , D. Yang , X. D. Peng , G. K. Feng , J. Tang , X. F. Zhu , R. Deng , Nat. Commun. 2021, 12, 2672. 33976130 10.1038/s41467-021-22618-x PMC8113546

[32] X. Du , X. Lu , X. Cao , Innovation Med. 2023, 1, 100008.

[33] X. Huang , X. Cao , Innovation Med. 2023, 1, 100043.

[34] Y. Nagai , A. J. Ambinder , Cancers 2023, 15, 3535. 37509198 10.3390/cancers15143535 PMC10377082

[35] C. Liang , G. Qiao , Y. Liu , L. Tian , N. Hui , J. Li , Y. Ma , H. Li , Q. Zhao , W. Cao , H. Liu , X. Ren , Eur. J. Med. Chem. 2021, 220, 113451. 33895500 10.1016/j.ejmech.2021.113451

[36] A. le Maire , S. Alvarez , P. Shankaranarayanan , A. R. Lera , W. Bourguet , H. Gronemeyer , Curr. Top Med. Chem. 2012, 12, 505. 22242853 10.2174/156802612799436687

[37] K. Ohta , E. Kawachi , N. Inoue , H. Fukasawa , Y. Hashimoto , A. Itai , H. Kagechika , Chem. Pharm. Bull. 2000, 48, 1504. 10.1248/cpb.48.1504 11045459

[38] W. Zhao , S. Li , R. Chen , J. Ni , X. Huang , S. Li , X. Lu , X. Cao , Innovation Life 2023, 1, 100014.

[39] X. Li , X. Chen , S. Gong , J. Zhao , C. Yao , H. Zhu , R. Xiao , Y. Qin , R. Li , N. Sun , X. Li , F. Dong , T. Zhao , Y. Pan , J. Yang , Theranostics 2023, 13, 2040. 37064877 10.7150/thno.80555 PMC10091882

[40] Y. Liu , Y. Song , S. Zhang , M. Diao , S. Huang , S. Li , X. Tan , Cell Discovery 2020, 6, 53. 32802403 10.1038/s41421-020-0184-9 PMC7400672

[41] K. R. Snapp , C. E. Heitzig , G. S. Kansas , Blood 2002, 99, 4494. 12036880 10.1182/blood.v99.12.4494

[42] C. Liao , J. An , S. Yi , Z. Tan , H. Wang , H. Li , X. Guan , J. Liu , Q. Wang , J. Cancer 2021, 12, 4109. 34093814 10.7150/jca.58268 PMC8176256

[43] S. Tomida , M. Takata , T. Hirata , M. Nagae , M. Nakano , Y. Kizuka , J. Biol. Chem. 2020, 295, 7992. 32350116 10.1074/jbc.RA120.013079 PMC7278346

[44] M. A. Järvå , M. Dramicanin , J. P. Lingford , R. Mao , A. John , K. E. Jarman , R. Grinter , E. D. Goddard‐Borger , J. Biol. Chem. 2020, 295, 6677. 32220931 10.1074/jbc.RA120.013291 PMC7212660

[45] K. Bastian , E. Scott , D. J. Elliott , J. Munkley , Int. J. Mol. Sci. 2021, 22, 455. 33466384 10.3390/ijms22010455 PMC7795606

[46] R. Honma , I. Kinoshita , E. Miyoshi , U. Tomaru , Y. Matsuno , Y. Shimizu , S. Takeuchi , Y. Kobayashi , K. Kaga , N. Taniguchi , Oncology 2015, 88, 298. 25572677 10.1159/000369495

[47] M. Noda , H. Okayama , Y. Kofunato , S. Chida , K. Saito , T. Tada , M. Ashizawa , T. Nakajima , K. Aoto , T. Kikuchi , W. Sakamoto , H. Endo , S. Fujita , M. Saito , T. Momma , S. Ohki , K. Kono , PLoS One 2018, 13, e0200315. 29975776 10.1371/journal.pone.0200315 PMC6033451

[48] E. Scott , J. Munkley , Int. J. Mol. Sci. 2019, 20, 1389. 30893936 10.3390/ijms20061389 PMC6470778

[49] C. Liang , T. Fukuda , T. Isaji , C. Duan , W. Song , Y. Wang , J. Gu , Biochim. Biophys. Acta. Gen. Subj. 2021, 1865, 129870. 33571582 10.1016/j.bbagen.2021.129870

[50] Z. Sadeghzadeh , A. Khosravi , M. S. Jazi , J. Asadi , Glycoconj. J. 2020, 37, 319. 32157457 10.1007/s10719-020-09917-z

[51] H. F. Yang , M. Yu , H. D. Jin , J. Q. Yao , Z. L. Lu , I. B. Yabasin , Q. Yan , Q. P. Wen , Front. Physiol. 2017, 8, 510. 28798691 10.3389/fphys.2017.00510 PMC5526971

[52] Z. Ye , H. Guo , L. Wang , Y. Li , M. Xu , X. Zhao , X. Song , Z. Chen , R. Huang , J. Mol. Cell Cardiol. 2022, 165, 54. 34974060 10.1016/j.yjmcc.2021.12.012

[53] R. Tinoco , D. C. Otero , A. A. Takahashi , L. M. Bradley , Trends Immunol. 2017, 38, 323. 28262471 10.1016/j.it.2017.02.002 PMC5411281

[54] M. Prorok‐Hamon , F. Notel , S. Mathieu , C. Langlet , M. Fukuda , A. El‐Battari , Biochem. J. 2005, 391, 491. 15926890 10.1042/BJ20050344 PMC1276950

[55] J. Eberhardt , D. Santos‐Martins , A. F. Tillack , S. Forli , J. Chem. Inf. Model 2021, 61, 3891. 34278794 10.1021/acs.jcim.1c00203 PMC10683950

[56] Y. Yan , D. Zhang , P. Zhou , B. Li , S. Y. Huang , Nucl. Acids Res. 2017, 45, W365. 28521030 10.1093/nar/gkx407 PMC5793843

[57] M. D. Robinson , D. J. McCarthy , G. K. Smyth , Bioinformatics 2010, 26, 139. 19910308 10.1093/bioinformatics/btp616 PMC2796818

[58] T. Wu , E. Hu , S. Xu , M. Chen , P. Guo , Z. Dai , T. Feng , L. Zhou , W. Tang , L. Zhan , X. Fu , S. Liu , X. Bo , G. Yu , Innovation 2021, 2, 100141. 34557778 10.1016/j.xinn.2021.100141 PMC8454663

[59] P. Shannon , A. Markiel , O. Ozier , N. S. Baliga , J. T. Wang , D. Ramage , N. Amin , B. Schwikowski , T. Ideker , Genome Res. 2003, 13, 2498. 14597658 10.1101/gr.1239303 PMC403769

[60] E. Coudert , S. Gehant , E. de Castro , M. Pozzato , D. Baratin , T. Neto , C. J. A. Sigrist , N. Redaschi , A. Bridge , Bioinformatics 2023, 39, btac793.36484697

[61] M. E. Ritchie , B. Phipson , D. Wu , Y. Hu , C. W. Law , W. Shi , G. K. Smyth , Nucl. Acids Res. 2015, 43, e47. 25605792 10.1093/nar/gkv007 PMC4402510

[62] F. Farshidfar , S. Zheng , M. C. Gingras , Y. Newton , J. Shih , A. G. Robertson , T. Hinoue , K. A. Hoadley , E. A. Gibb , J. Roszik , K. R. Covington , C. C. Wu , E. Shinbrot , N. Stransky , A. Hegde , J. D. Yang , E. Reznik , S. Sadeghi , C. S. Pedamallu , A. I. Ojesina , J. M. Hess , J. T. Auman , S. K. Rhie , R. Bowlby , M. J. Borad , C. G. A. Network , A. X. Zhu , J. M. Stuart , C. Sander , R. Akbani , et al., Cell Rep. 2017, 18, 2780. 28297679 10.1016/j.celrep.2017.02.033 PMC5493145

[63] N. Oishi , M. R. Kumar , S. Roessler , J. Ji , M. Forgues , A. Budhu , X. Zhao , J. B. Andersen , Q. H. Ye , H. L. Jia , L. X. Qin , T. Yamashita , H. G. Woo , Y. J. Kim , S. Kaneko , Z. Y. Tang , S. S. Thorgeirsson , X. W. Wang , Hepatology 2012, 56, 1792. 22707408 10.1002/hep.25890 PMC3458130

