# Supplementary material for: Synthetic Retinoid Sulfarotene Selectively Inhibits Tumor‐Repopulating Cells of Intrahepatic Cholangiocarcinoma via Disrupting Cytoskeleton by P‐Selectin/PSGL1 N‐Glycosylation Blockage
Source: Adv Sci (Weinh). 2024 Nov 28;12(3):2407519. doi: 10.1002/advs.202407519 (PMC11744644; doi:10.1002/advs.202407519)
Supplement: Supplementary file 1 — Supporting Information [file ADVS-12-2407519-s001.docx]

Supporting Information

Title Synthetic retinoid sulfarotene selectively inhibits tumor-repopulating cells of intrahepatic cholangiocarcinoma *via* disrupting cytoskeleton by P-selectin/PSGL1 N-glycosylation blockage

*Xiaojing Du*^1,2^†, *Zhuoran Qi*^1^†, *Sinuo Chen*^1^†, *Jinlan Wu*^3^†, *Ye Xu*^1^, *Sunkuan Hu*^4^, *Zhijie Yu*^5,6^, *Jiayun Hou^7^*, Y*uan Fang*^8^*, *Jinglin Xia*^1,9^*, *Xin Cao*^10^*

**Supplementary materials and methods**

***Colony formation assay***

HUCCT1 or RBE (5 × 10^3^ cells/well) were incubated in a 12-well plate and attached overnight. After different treatments for 1 week, the cells were stained with 1% crystal violet solution (V5265, Sigma). The colonies were recorded using camera, and followed the crystal violet was eluted using 30% acetic acid (1 ml). Then, 100 μl eluent was added to a 96-well plate, and its OD value was detected at 600 nm using FlexStation 3 Multi-Mode Microplate Reader.

***EdU staining***

EdU staining is a commonly used assay for detecting cell proliferation. It was performed on 2D ICC cells using Cell-Light EdU Apollo567 In Vitro Kit (C10310-1, RIBOBIO) according to the manufacturer’s instructions.

***Scratch assay***

HUCCT1 or RBE cells were seeded in a pre-marked 6-well plate. Until reaching 100% cell confluence, a 200 μl pipette tip was used to scratch the surface of the cells. The cell debris were cleaned using PBS. The scratches at 0 h, 24 h and 48 h were photographed after treatment. Image J soft was used to calculate the scratch area and migration index was determined as followed. Migration index = (scratch area of 0 h - scratch of 24 h or 48 h)/scratch area of 0 h × 100%.

***Fluo-4 calcium assay***

Fluo-4 calcium assay kit (S1061S, Beyotime) was used to detect the concentration of intracellular calcium in ICC-TRCs according to the manufacturer's instructions.

***LCA staining and H&E staining***

For LCA staining, after deparaffinization and rehydration, the slices were blocked with Carbo-Free^TM^ Blocking Solution (SP-5040, Vector laboratories) at room temperature for 30 min. The slices were incubated with 20 μg/ml LCA/PBS at room temperature for 45 min, and then with horseradish peroxidase streptavidin at room temperature for 30 min. The staining was completed using DAB kit and the nuclear was stained using hematoxylin (C0105S, Beyotime) according to the manufacturer's manual. For H & E staining, after deparaffinization and rehydration, the slices of tumor tissues were stained with hematoxylin and eosin according to the manufacturer's manual. All slices were observed on a SLIDEVIEW VS200 (Olympus, Tokyo, Japan).

***DMPK analysis***

DMPK analysis, including *in vitro* permeability test, *in vitro* metabolites identification, *in vivo* stability and toxicity assay, was conducted by WUXI AppTec.

***Statistics***

R (version 4.2.0) and Graphpad Prism 8.0 software were applied to statistics analyses and plotting. T test and Tukey's multiple comparisons test was performed to achieve the statistics analysis of experimental data. All data were expressed as mean  ± SD and significance was defined as *p* ≤ 0.05 unless otherwise mentioned.


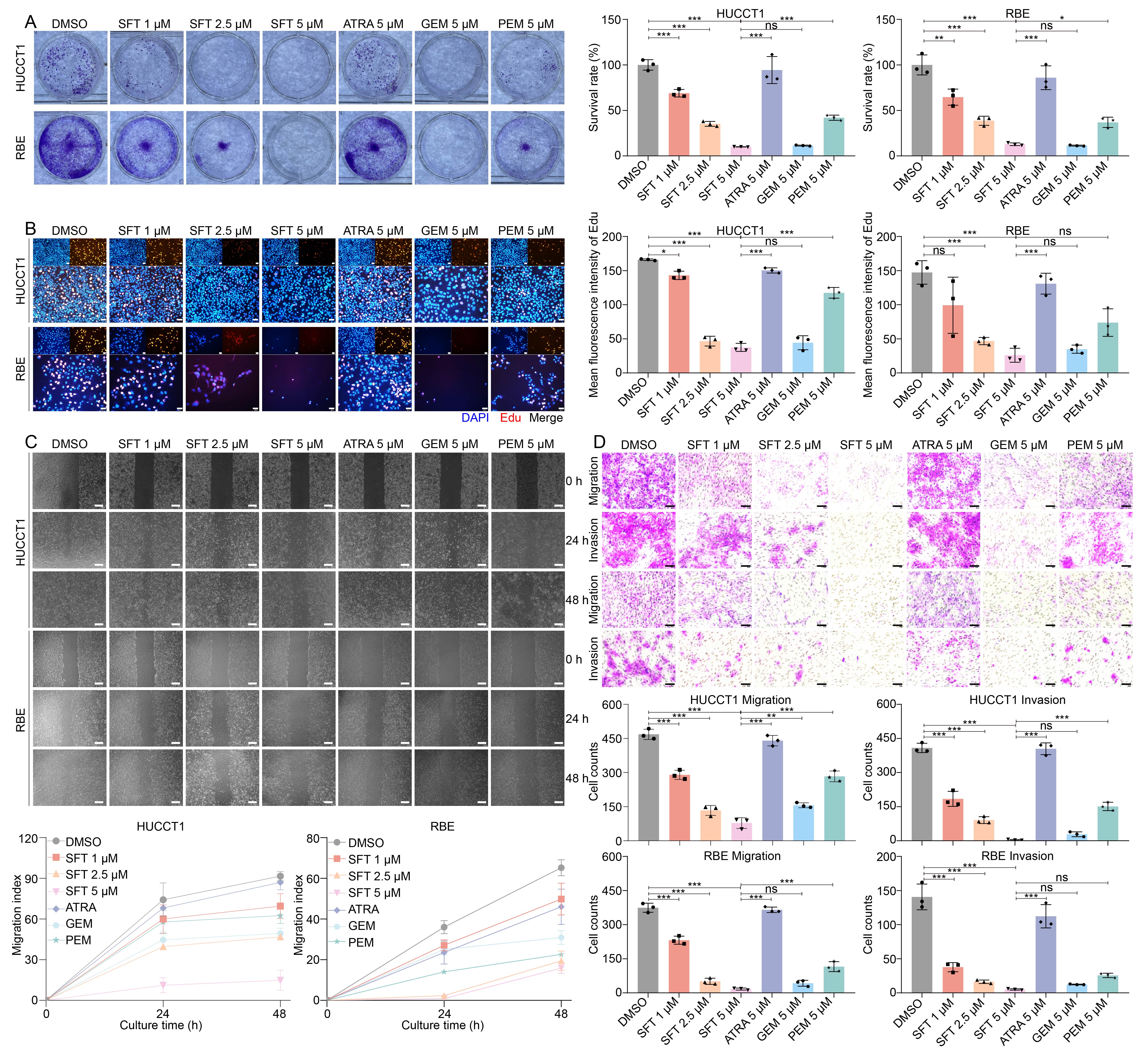


Figure S1. The inhibition of SFT on 2D ICC cell lines.

A. HUCCT1 or RBE cells were treated with SFT (1, 2.5 and 5 μM), ATRA (5 μM), GEM (5 μM) and PEM (5 μM) and cultured for 1 weeks, then cell colony was visualized using crystal violet and quantified using OD value after 30% acetic acid eluting (*n* = 3, Tukey's multiple comparisons test). B. HUCCT1 or RBE cells were treated with SFT (1, 2.5 and 5 μM), ATRA (5 μM), GEM (5 μM) and PEM (5 μM) for 48 h, and EdU staining was performed. The fluorescence intensity was calculated using Image J soft (*n* = 3, Tukey's multiple comparisons test). C. HUCCT1 or RBE cells were treated with SFT (1, 2.5 and 5 μM), ATRA (5 μM), GEM (5 μM) and PEM (5 μM) for 48 h, the scratches were photographed at 0 h, 24 h and 48 h after treatment and quantified using Image J. D. After HUCCT1 or RBE cells were treated with SFT (1, 2.5 and 5 μM), ATRA (5 μM), GEM (5 μM) and PEM (5 μM) for 48 h, transwell assay was used to detect the effect of agents on the migration and invasion of ICC cells. Up panel, the representative images. Down panel, quantitative analysis of Transwell assay calculated using Image J (*n* = 3, Tukey's multiple comparisons test). ICC, Intrahepatic cholangiocarcinoma; SFT, sulfarotene; ATRA, all-trans retinoic acid; GEM, gemcitabine; PEM, pemigatinib. Data are presented as the mean ± SD; **p* < 0.05, ***p* < 0.01, ****p* < 0.001. ns, not significant.

**
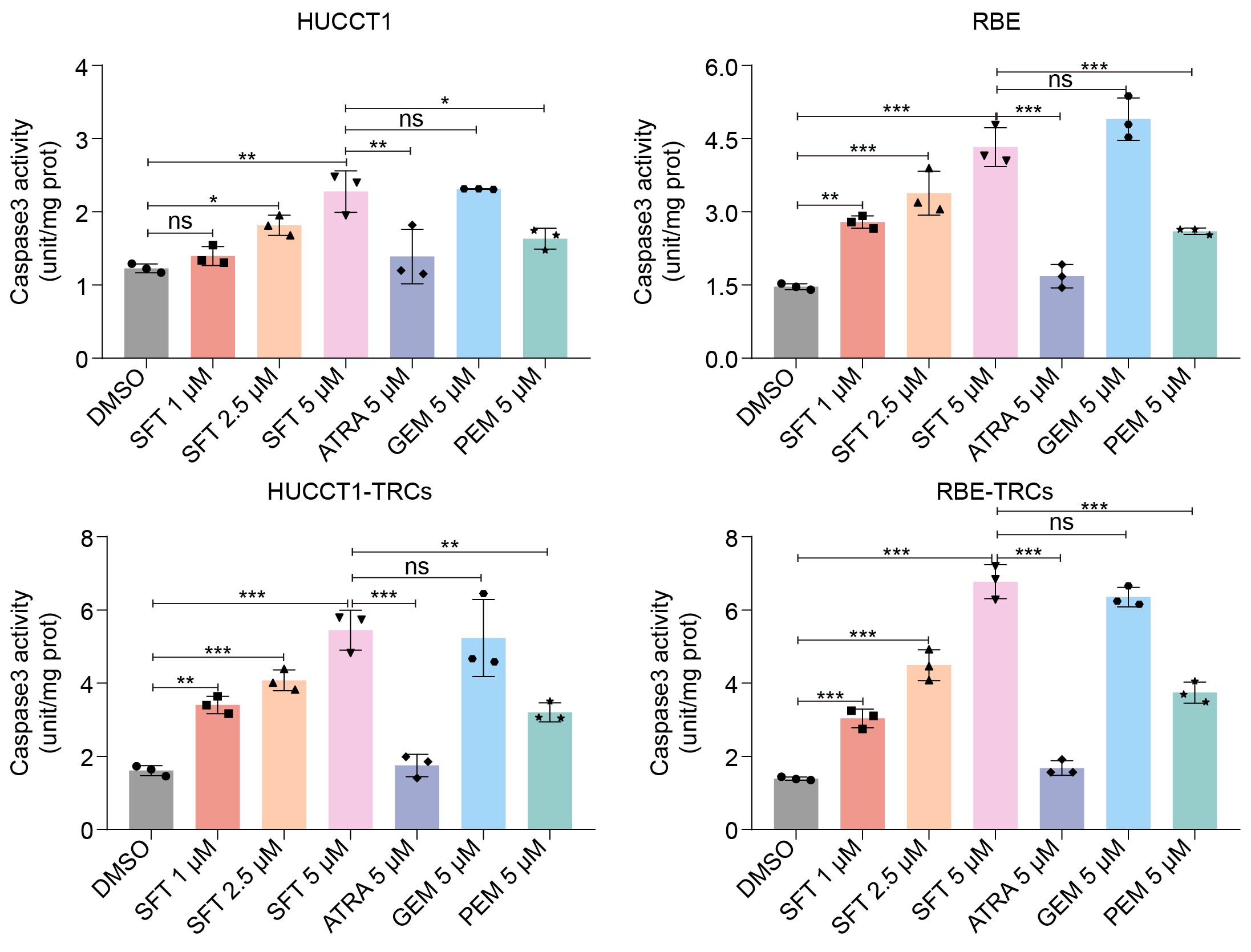
**

**Figure S2. SFT increased caspase3 activity**.

HUCCT1, RBE, HUCCT1-TRCs, and RBE-TRCs were treated with SFT (1, 2.5 and 5 μM), ATRA (5 μM), GEM (5 μM) and PEM (5 μM) for 48 h (*n* = 3, Tukey's multiple comparisons test). The caspase3 activity was detected by caspase3 activity assay kit. SFT, sulfarotene; TRCs, tumor-repopulating cells; ATRA, all-trans retinoic acid; GEM, gemcitabine; PEM, pemigatinib. Data are presented as the mean ± SD; **p* < 0.05, ***p* < 0.01, ****p* < 0.001. ns, not significant.


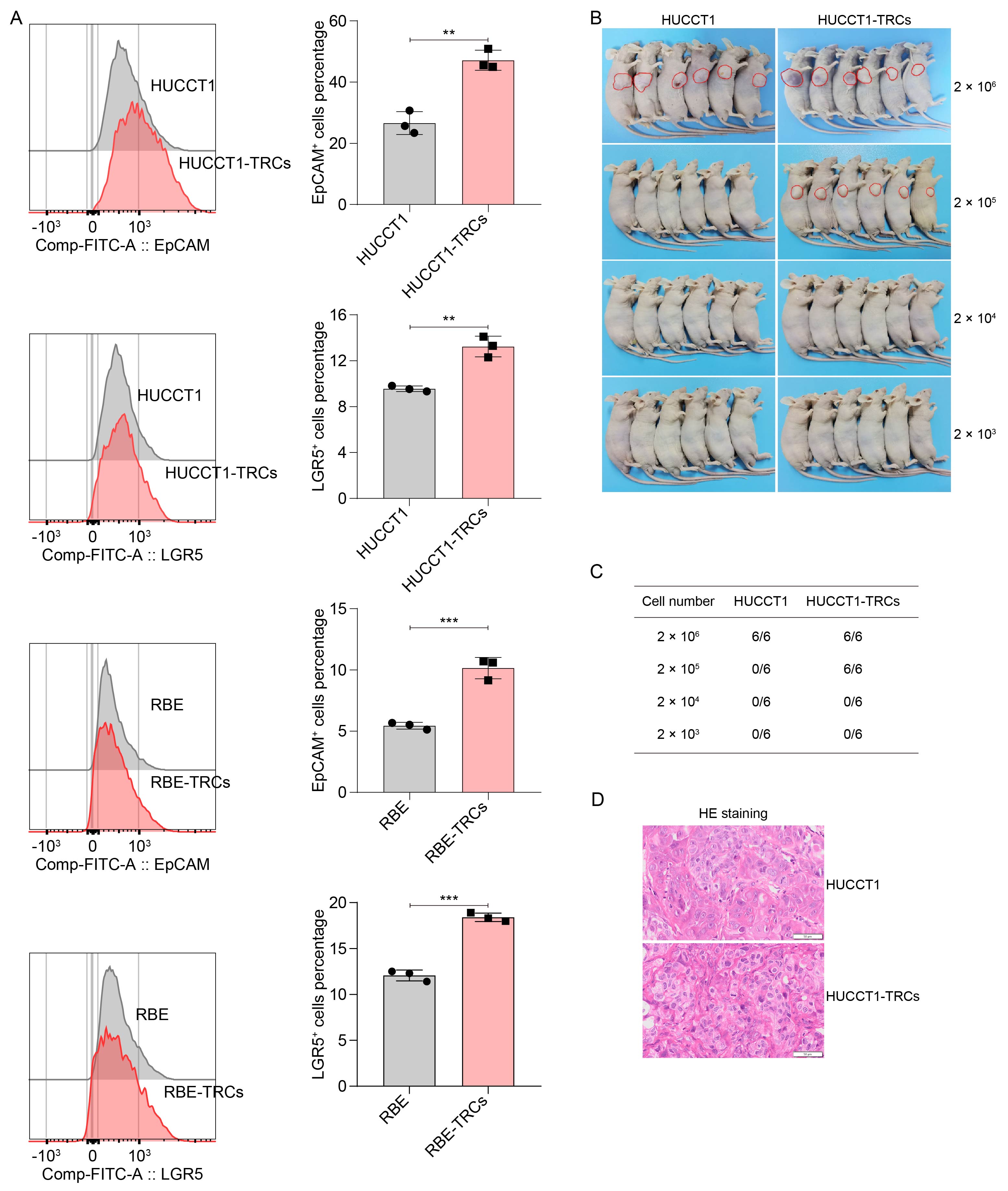


**Figure S3. HUCCT1-TRCs are more prone to tumorigenesis.**

A. EpCAM and LGR5 positive cells were detected by flow cytometry (*n* = 3, t test). Data are presented as the mean ± SD; ***p* < 0.01, ****p* < 0.001. B. Different number cells of HUCCT1 and HUCCT1-TRCs were injected into mice. The pictures showed the subcutaneous tumor at 8 weeks after injection. C. The table showed the tumor formation rate of different cells. D. The H & E staining of different cell formed tumor. TRCs, tumor-repopulating cells.


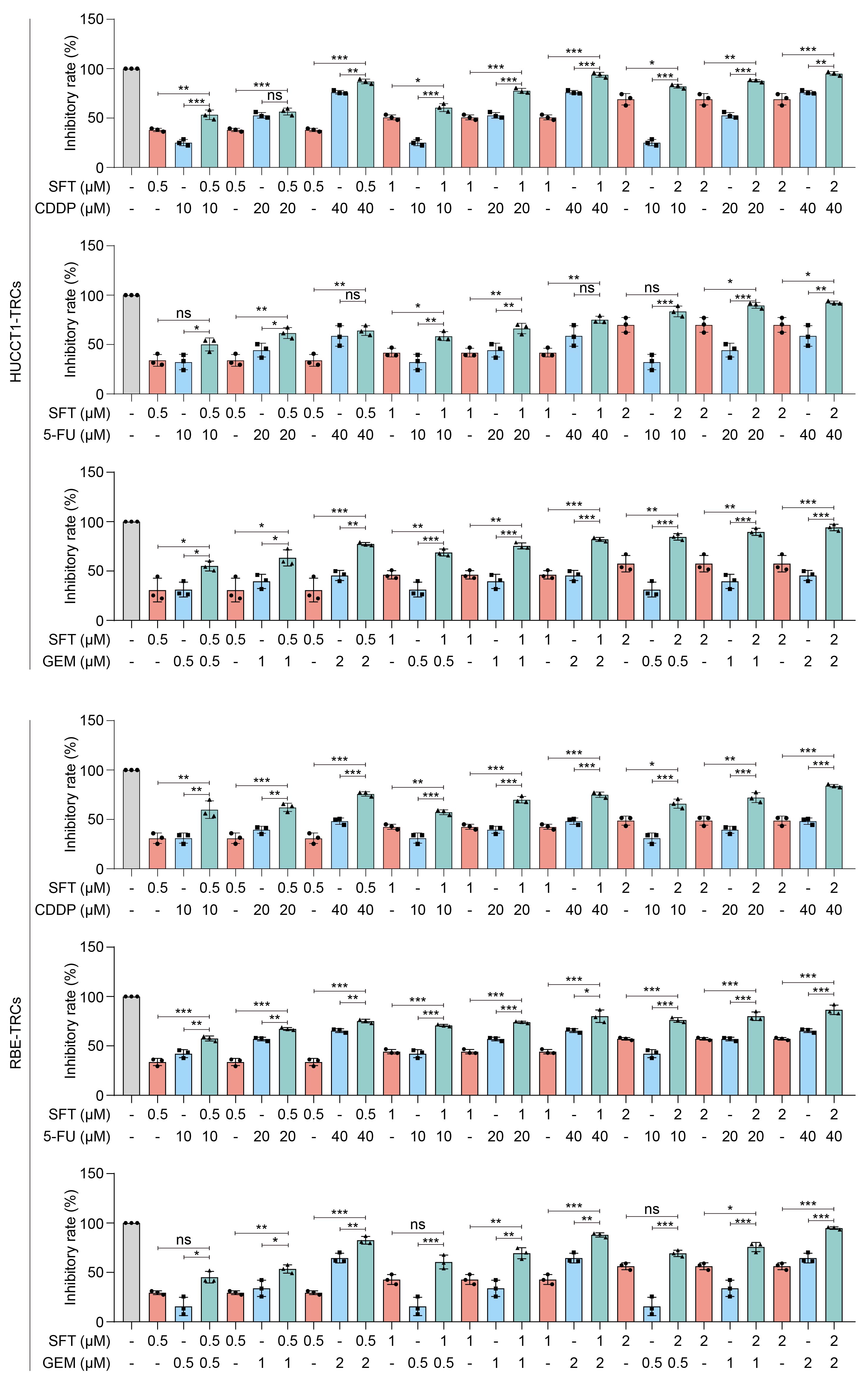


**Figure S4. The combination efficacy of SFT and chemotherapy on ICC-TRCs.**

The inhibitory rate of SFT alone, chemotherapy (5-FU, CDDP, and GEM) alone or combination was detected using CCK8 on HUCCT1-TRCs and RBE-TRCs. ICC, Intrahepatic cholangiocarcinoma; TRCs, tumor-repopulating cells; SFT, sulfarotene; 5-FU, 5-fluorouracil; CDDP, cisplatin; GEM, gemcitabine. Data are presented as the mean ± SD; *n* = 3, Tukey's multiple comparisons test; ***p* < 0.01, ****p* < 0.001. ns, not significant.


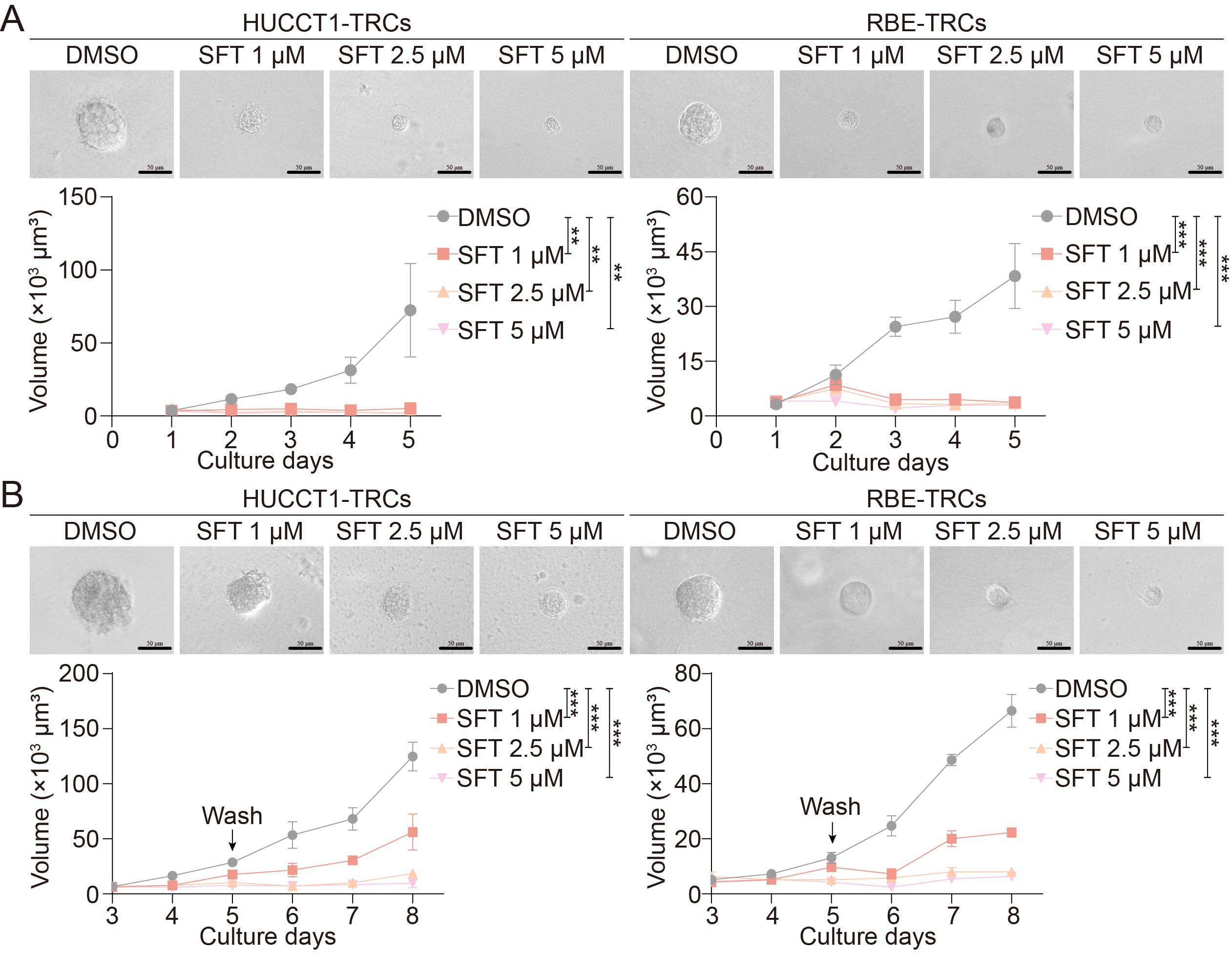


**Figure S5. SFT inhibited the formation of colony spheroid.**

A. HUCCT1-TRCs or RBE-TRCs were treated with SFT (1, 2.5 and 5 μM) immediately when they were cultured in 3D fibro gel (*n* = 3, Tukey's multiple comparisons test). B. After cultured in 3D fibro gel for 72 h, HUCCT1-TRCs or RBE-TRCs were treated with SFT (1, 2.5 and 5 μM) for 48 h. Then eluting drugs and kept on recording (*n* = 3, Tukey's multiple comparisons test). TRCs, tumor-repopulating cells; SFT, sulfarotene. Data are presented as the mean ± SD; ***p* < 0.01, ****p* < 0.001. ns, not significant.


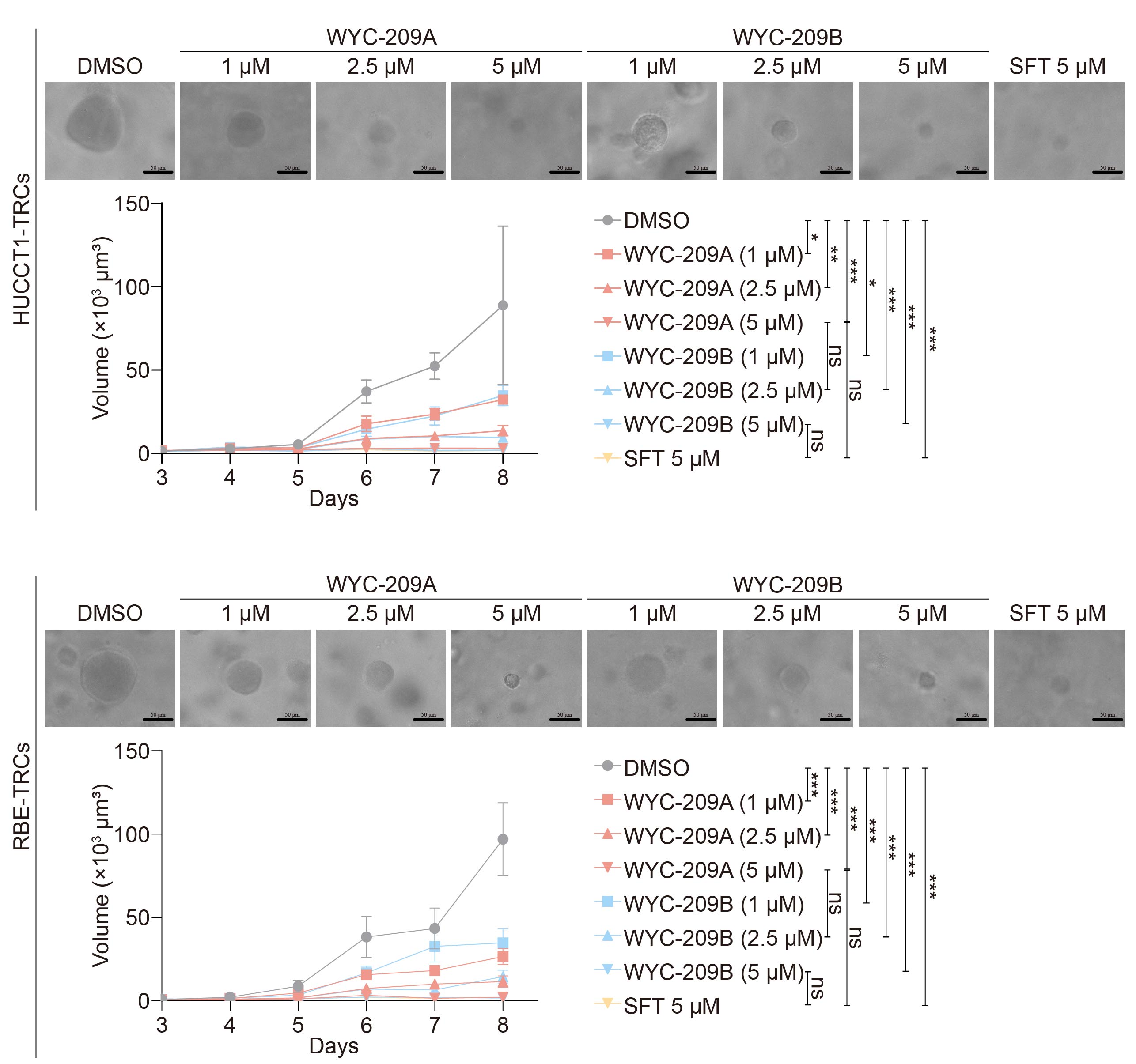


**Figure S6. WYC-209A and WYC-209B inhibited the growth of colony spheroid.**

After HUCCT1-TRCs or RBE-TRCs cultured for 72 h, the cells were treated with WYC-209A (1, 2.5 and 5 μM), WYC-209B (1, 2.5 and 5 μM) , and SFT (5 μM) (*n* = 3, Tukey's multiple comparisons test). Data are presented as the mean ± SD; **p* < 0.05, ***p* < 0.01, ****p* < 0.001. ns, not significant.


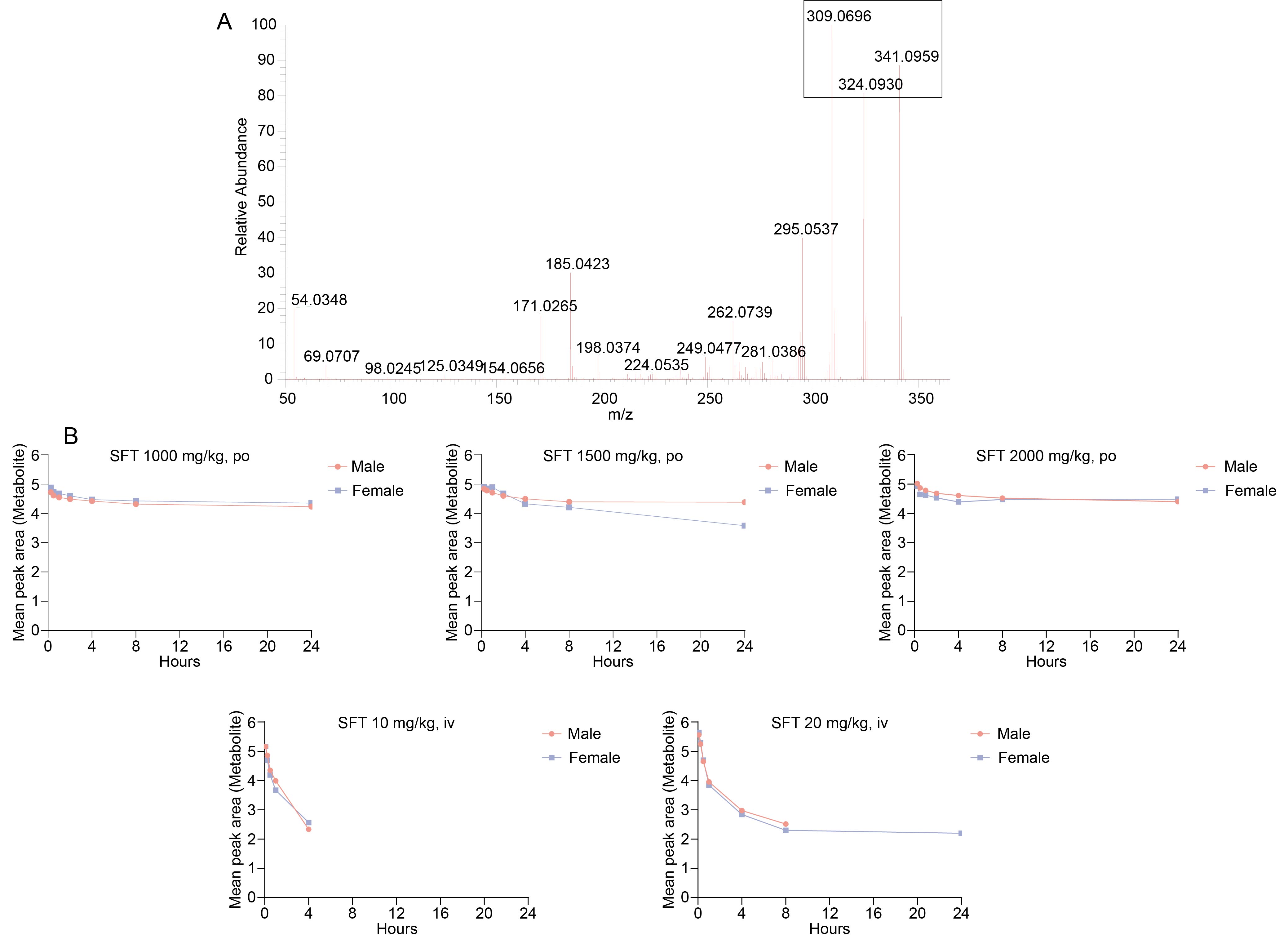


**Figure S7. The stability analysis of SFT.**

A. After iv administration of SFT, the metabolites were detected using mass spectrometry. B. The degradation of SFT metabolites was detected after oral administration (po; 0.25, 0.5, 1, 2, 4, 8, 24 h) or iv administration (0.083, 0.25, 0.5, 1, 4, 8, 24 h) of SFT on rat. iv, intravenous injection.

**
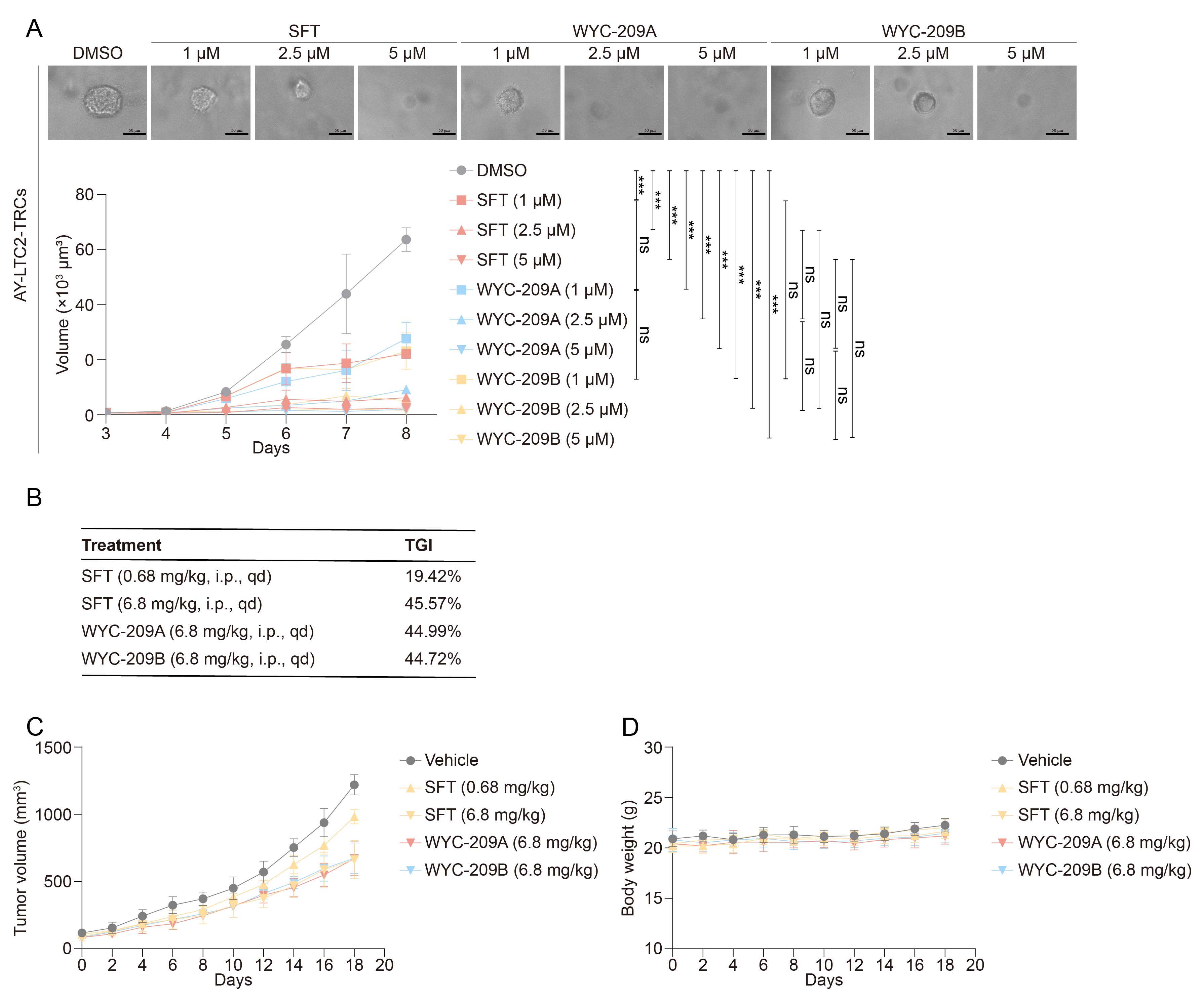
**

**Figure S8. SFT inhibits AY-LTC2-TRCs *in vitro* and *in vivo*.**

A. The inhibition of SFT, WYC-209A and WYC-209B on the growth of AY-LTC2-TRCs colony spheroid (*n* = 3, Tukey's multiple comparisons test). B & C. Inhibitory effects of SFT, WYC-209A and WYC-209B on the growth of xenograft tumor of AY-LTC2-TRCs subcutaneously transplanted to the flanks of C57BL/6 mice (*n* = 6). After tumor grew to 100 mm^3^, mice were treated with SFT (0.68 or 6.8 mg/kg, i.p., qd) compared to WYC-209A (6.8 mg/kg, i.p., qd), WYC-209B (6.8 mg/kg, i.p., qd) and the carrier. D. The weight of mice changed after treatment of SFT, WYC-209A and WYC-209B. TGI, tumor growth inhibition. Data are presented as the mean ± SD; ****p* < 0.001. ns, not significant.


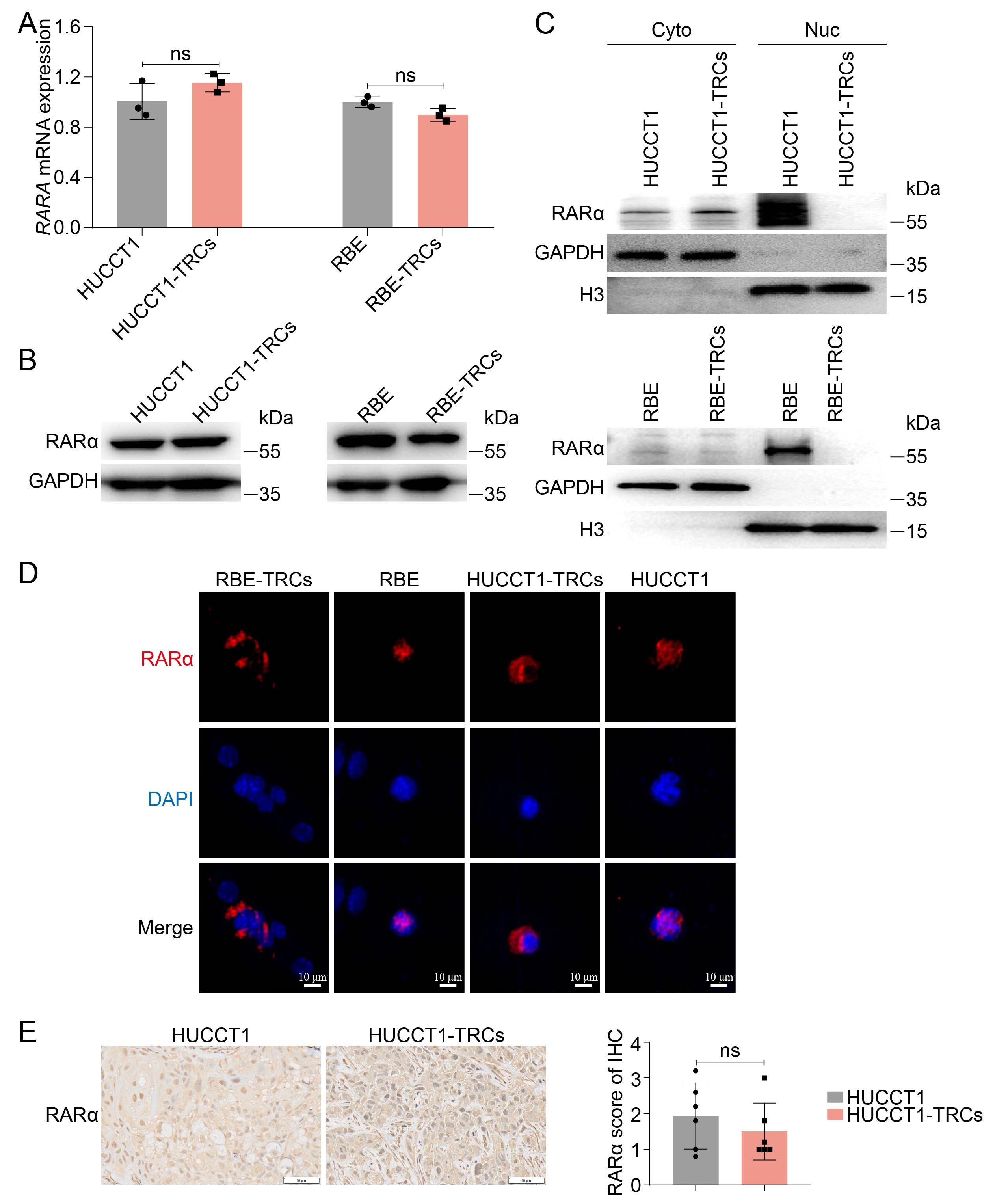


**Figure S9. The expression and cellular location of RARɑ.**

A & B. The level of RARɑ mRNA and protein was detected using qRT-PCR (A, *n* = 3, t test) and IB (B), respectively. C. Nuclear and cytoplasmic proteins were separated and detected using IB. D. The cellular location of RARɑ was detected using IF. Scale: 10 μm. E. The expression and cellular location of RARɑ was investigated using IHC in tumor tissues and quantified using Image J (*n* = 6, t test). TRCs, tumor-repopulating cells; SFT, sulfarotene; IB, immunoblotting; IF, immunofluorescence. Data are presented as the mean ± SD; ns, not significant.


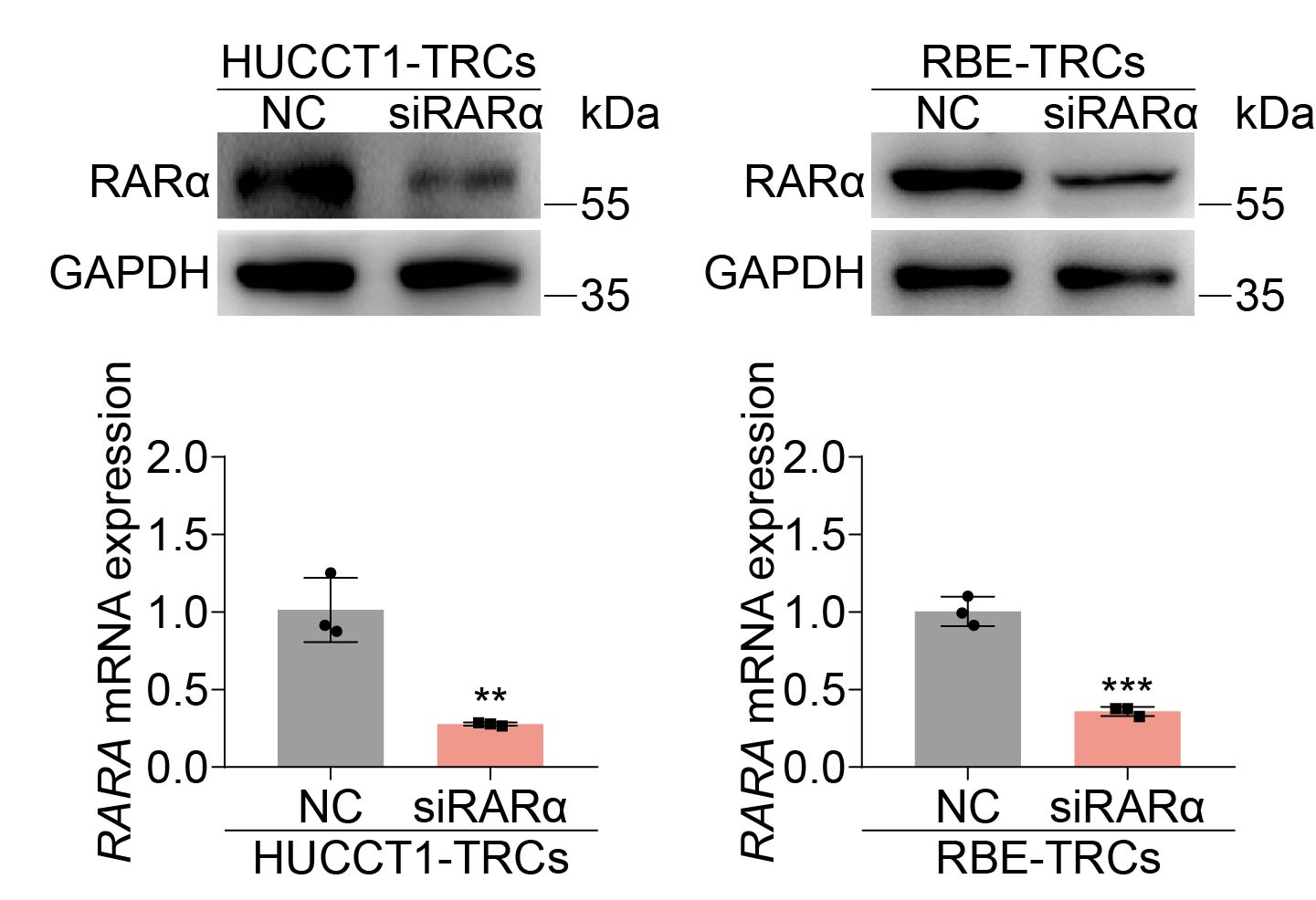


**Figure S10. Effect verification of siRARɑ in HUCCT1-TRCs and RBE-TRCs.**

The level of RARɑ protein and *RARA* mRNA was detected at 72 h post-transfection of siRARɑ. TRCs, tumor-repopulating cells.

**
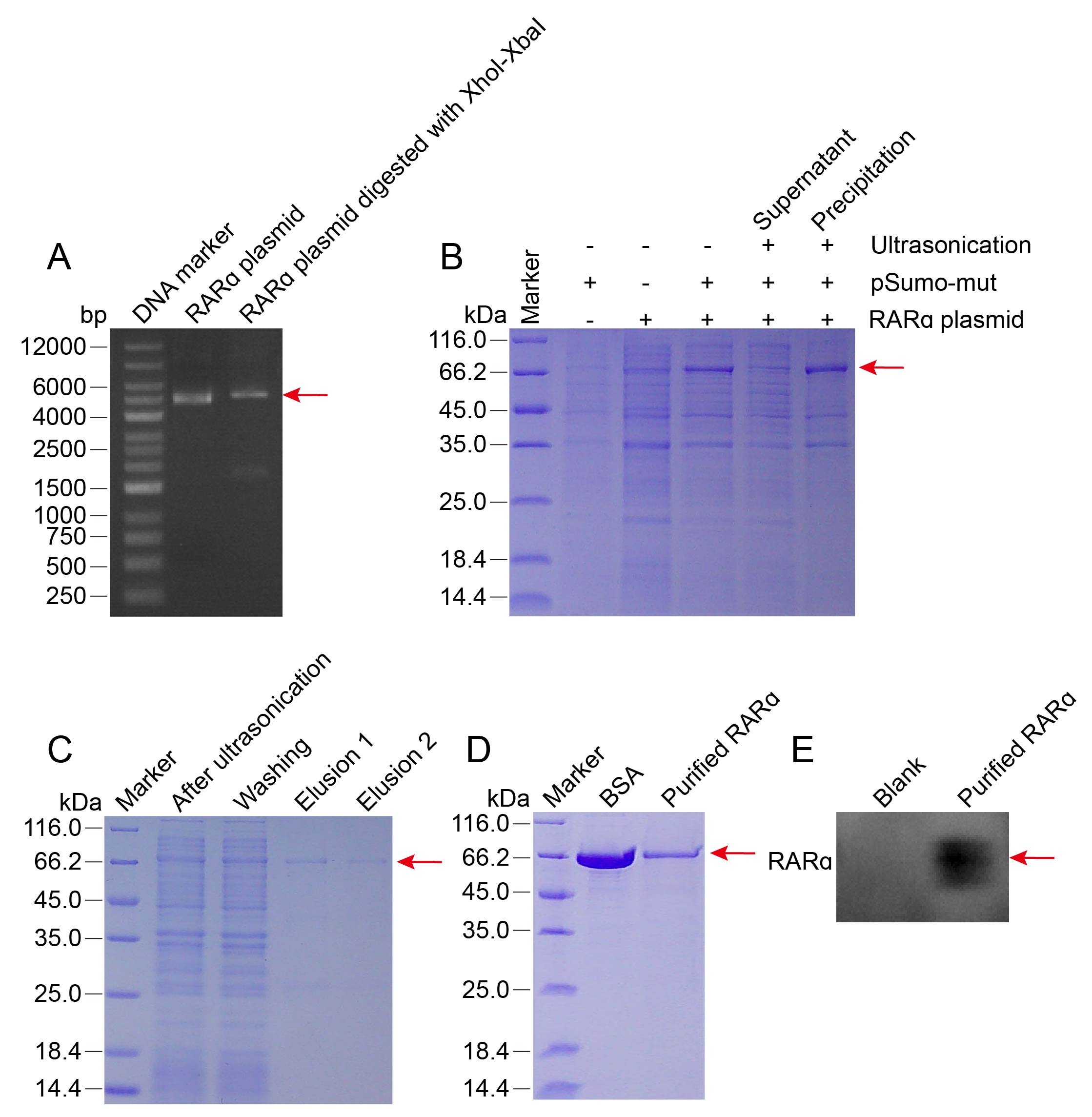
**

**Figure S11. Identification of the purified RARɑ protein.**

A. Restriction digestion map. B. RARɑ expression identification by SDS-PAGE analysis. C. SDS-PAGE analysis of RARɑ purification. D. Quality control of RARɑ by SDS-PAGE analysis. E. Quality control of RARɑ by IB. IB, immunoblotting.


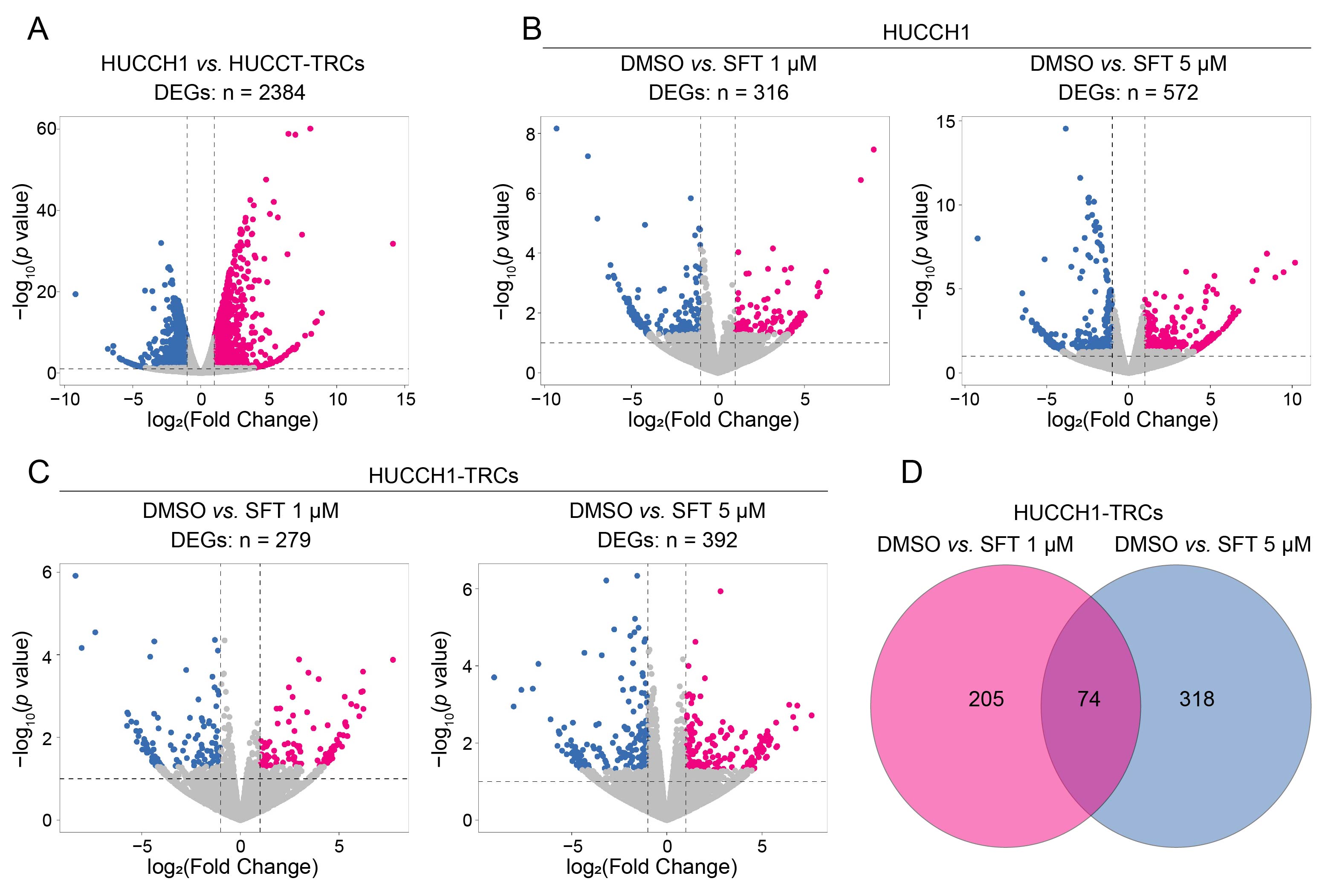


**Figure S12. Differentially gene analysis of HUCC1 and HUCCT1-TRCs after RNA-seq.**

A. Volcano plot showed the DEGs between HUCCT1 and HUCCT1-TRCs. B-C. Volcano plot showed the DEGs in SFT (1 and 5 μM) treated HUCCT1 (B) and HUCCT1-TRCs (C). D. Venn plot showed the overlapped DEGs. *n* = 3. TRCs, tumor-repopulating cells; DEGs, differentially expressed genes; SFT, sulfarotene.


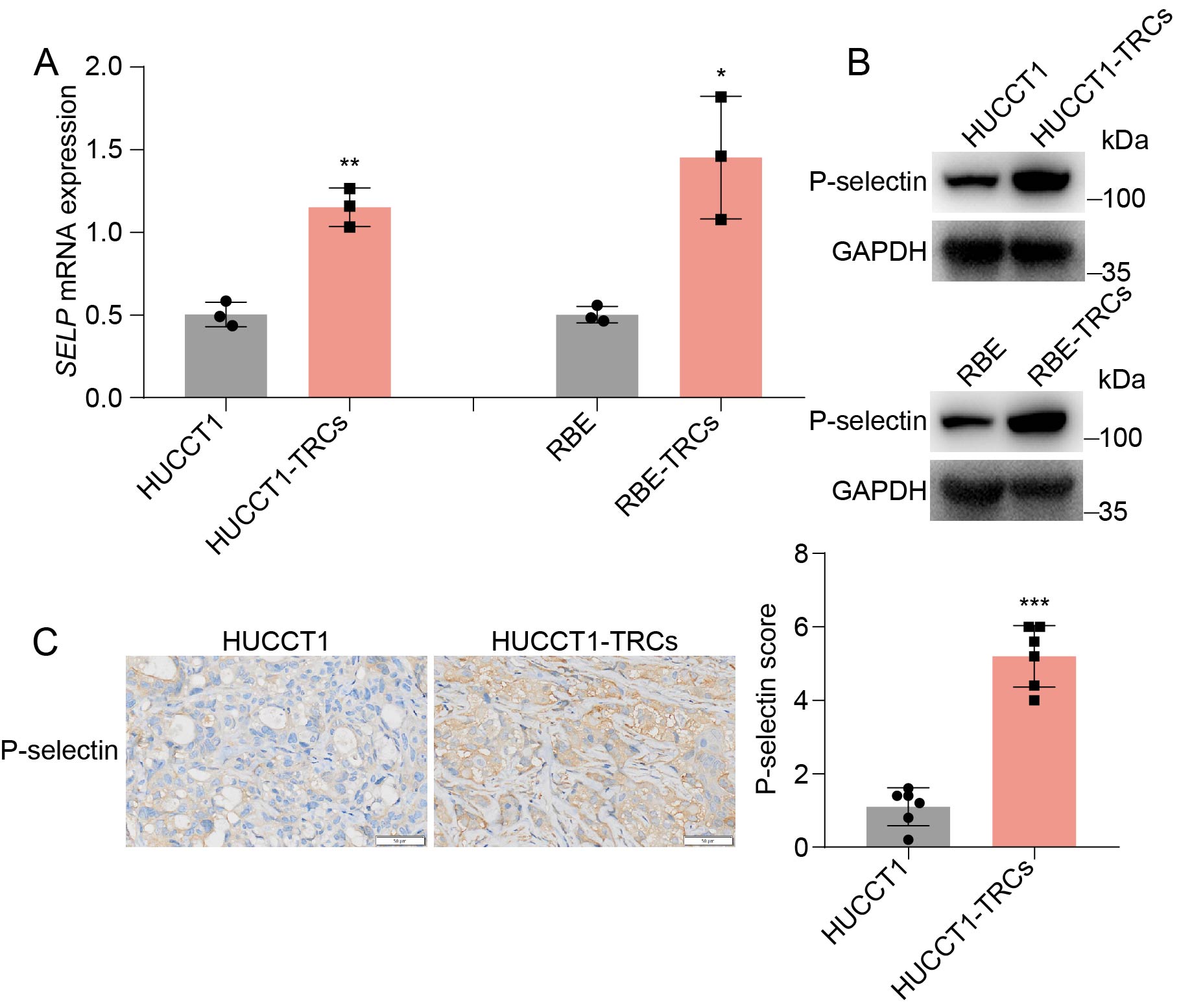


**Figure S13. P-selectin was up-regulated in ICC-TRCs.**

A. The level of *SELP* mRNA in 2D ICC cells and ICC-TRCs was detected using qRT-PCR (*n* = 3, t test). B. The level of P-selectin in 2D ICC cells and ICC-TRCs was detected using IB. C. The level of P-selectin in tumor tissues was detected using IHC and analyzed using Image J (*n* = 6, t test). ICC-TRCs, tumor-repopulating cells of intrahepatic cholangiocarcinoma; qRT-PCR, quantitative reverse transcription polymerase chain reaction; IB, immunoblotting; IHC, immunohistochemistry. Data are presented as the mean ± SD; **p* < 0.05, ***p* < 0.01, ****p* < 0.001.


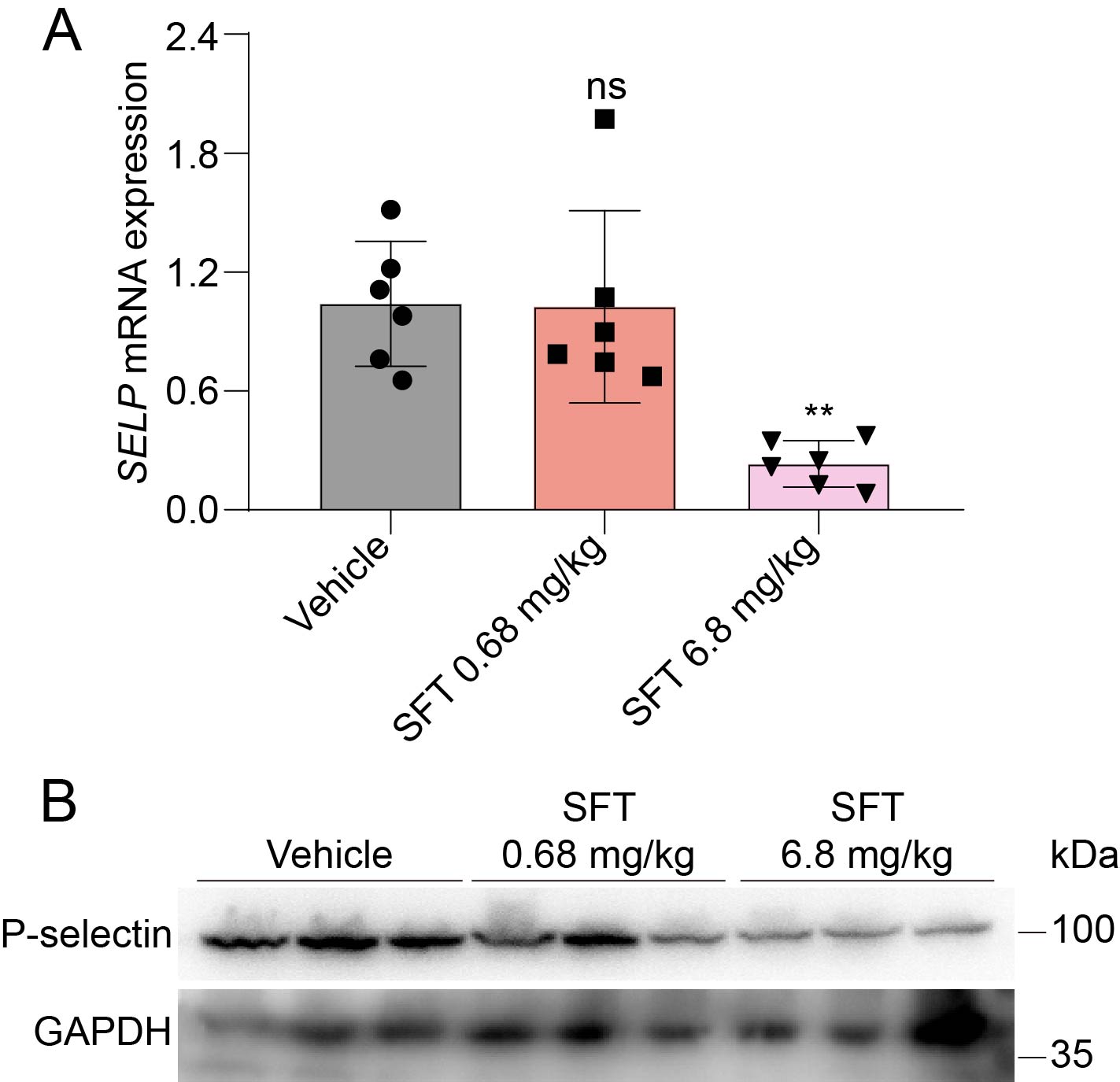


**Figure S14. SFT decreased the level of P-selectin.**

A. The mRNA was exacted from tumor tissues and the level of *SELP* mRNA was detected using qRT-PCR (*n* = 6, Tukey's multiple comparisons test). B. The level of P-selectin in tumor tissues was detected using IB. SFT, sulfarotene; qRT-PCR, quantitative reverse transcription polymerase chain reaction; IB, immunoblotting. Data are presented as the mean ± SD; ***p* < 0.01, ns, not significant.


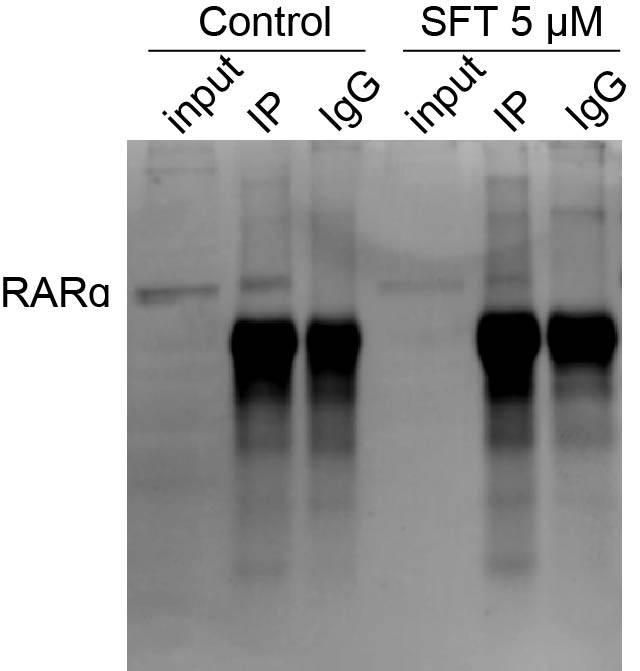


**Figure S15. Quality control of RARɑ pull down by immunoblotting.**


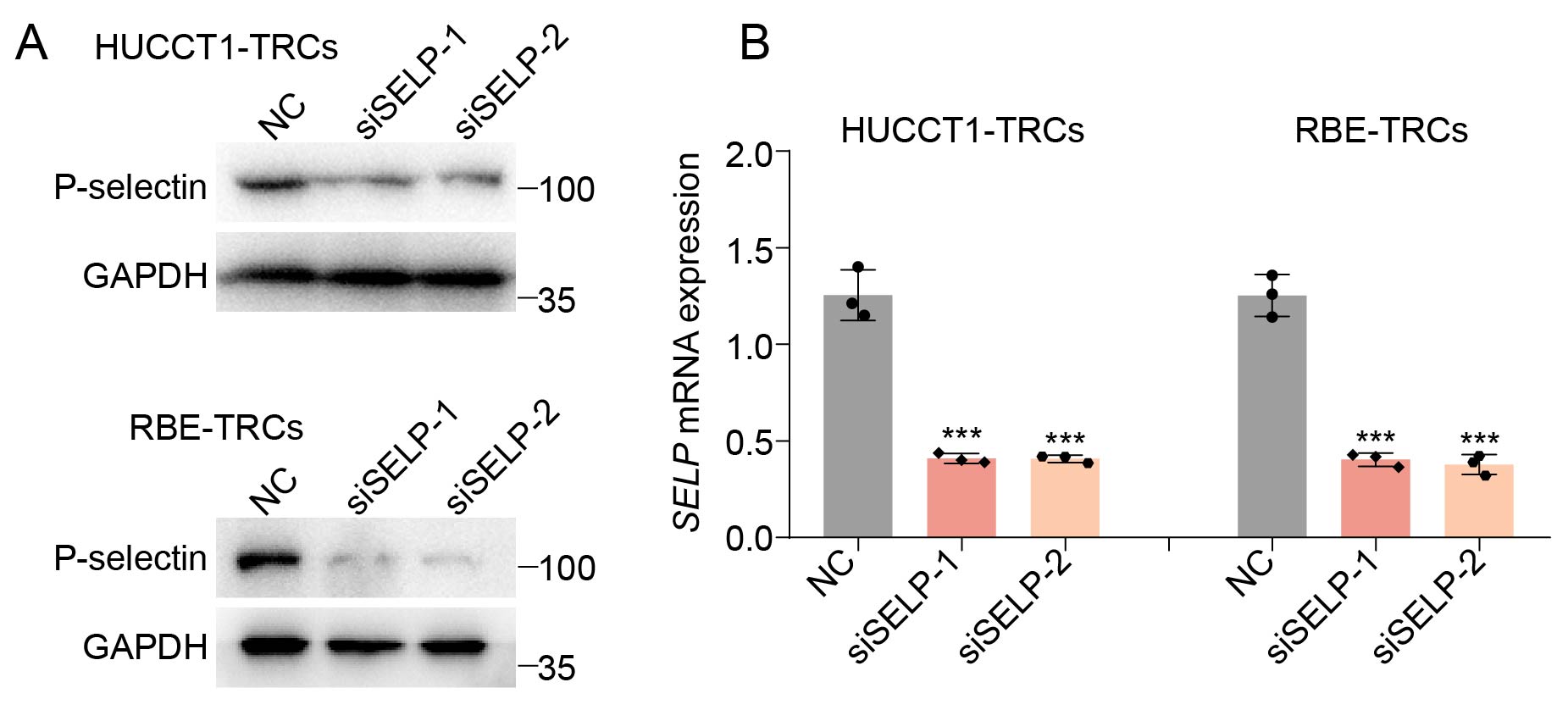


**Figure S16. The expression of SELP was silenced in ICC-TRCs**.

The level of P-selectin and *SELP* mRNA was detected by IB (A) and qRT-PCR (B, *n* = 3, t test), respectively. Data are presented as the mean ± SD; ICC-TRCs, tumor-repopulating cells of intrahepatic cholangiocarcinoma; IB, immunoblotting; qRT-PCR, quantitative reverse transcription polymerase chain reaction. ****p* < 0.001.


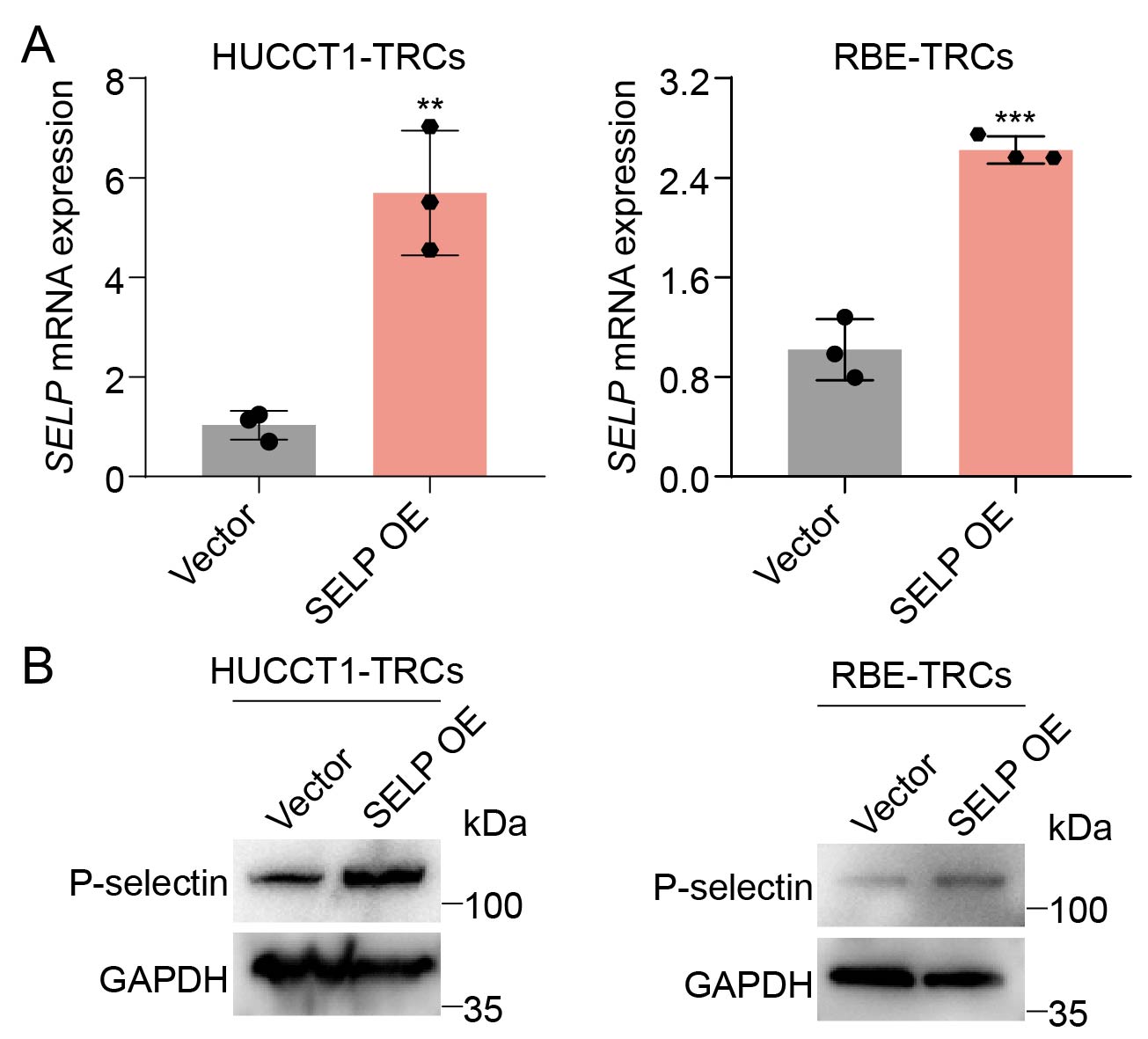


**Figure S17. P-selectin was over-expressed in ICC-TRCs.**

The level of *SELP* mRNA and P-selectin after transfection was detected by qRT-PCR (A, *n* = 3, t test) and IB (B), respectively. Data are presented as the mean ± SD; ICC-TRCs, tumor-repopulating cells of intrahepatic cholangiocarcinoma; OE, over-expression; qRT-PCR, quantitative reverse transcription polymerase chain reaction; IB, immunoblotting. ***p* < 0.01, ****p* < 0.001.


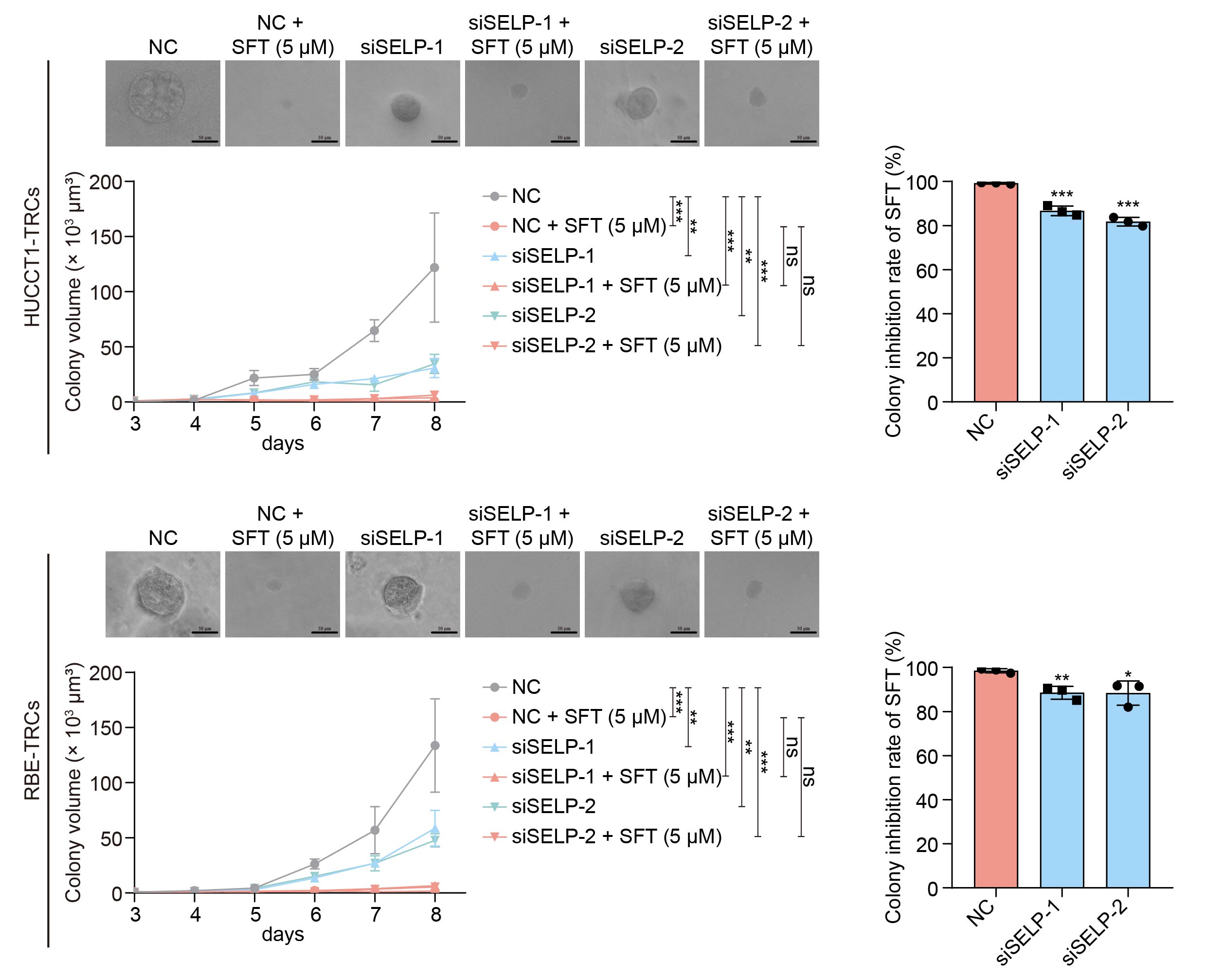


**Figure S18. Silencing P-selectin decreased the inhibition of SFT on ICC-TRCs.**

ICC-TRCs, tumor-repopulating cells of intrahepatic cholangiocarcinoma; SFT, sulfarotene. Data are presented as the mean ± SD; *n* = 3, Tukey's multiple comparisons test or t test; **p* < 0.05, ***p* < 0.01, ****p* < 0.001.


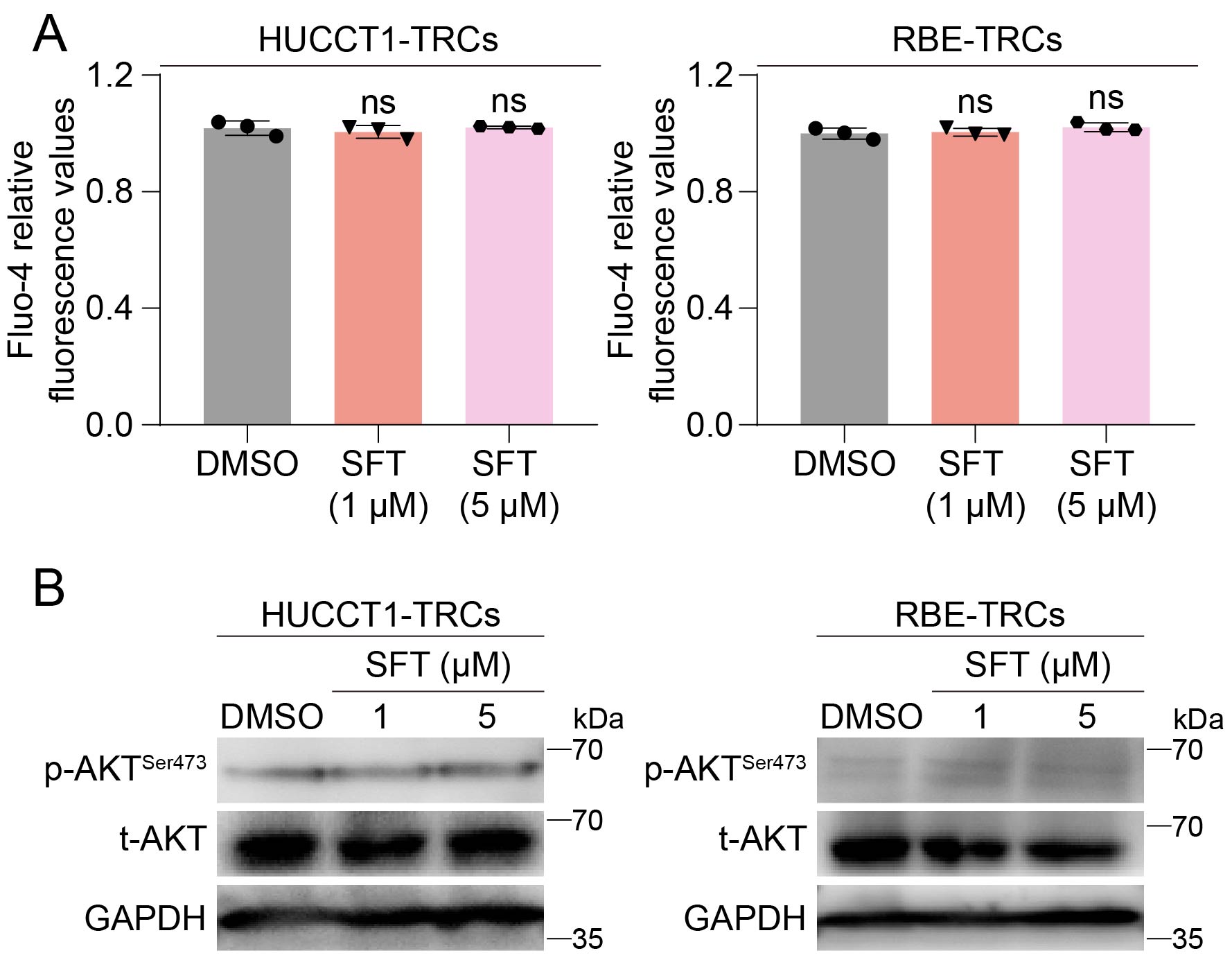


**Figure S19. SFT had no effects on calcium influx and AKT signaling.**

A. HUCCT1-TRCs or RBE-TRCs were treated with SFT (1 μM or 5 μM ) for 48 h, the concentration of intracellular calcium was detected using Fluo-4 calcium assay kit (*n* = 3, Tukey's multiple comparisons test). B. HUCCT1-TRCs or RBE-TRCs were treated with SFT (1 μM or 5 μM ) for 48 h, IB was used to investigate the alteration of p-AKT caused by SFT treatment. SFT, sulfarotene; TRCs, tumor-repopulating cells; IB, immunoblotting. Data are presented as the mean ± SD; ns, not significant.

**
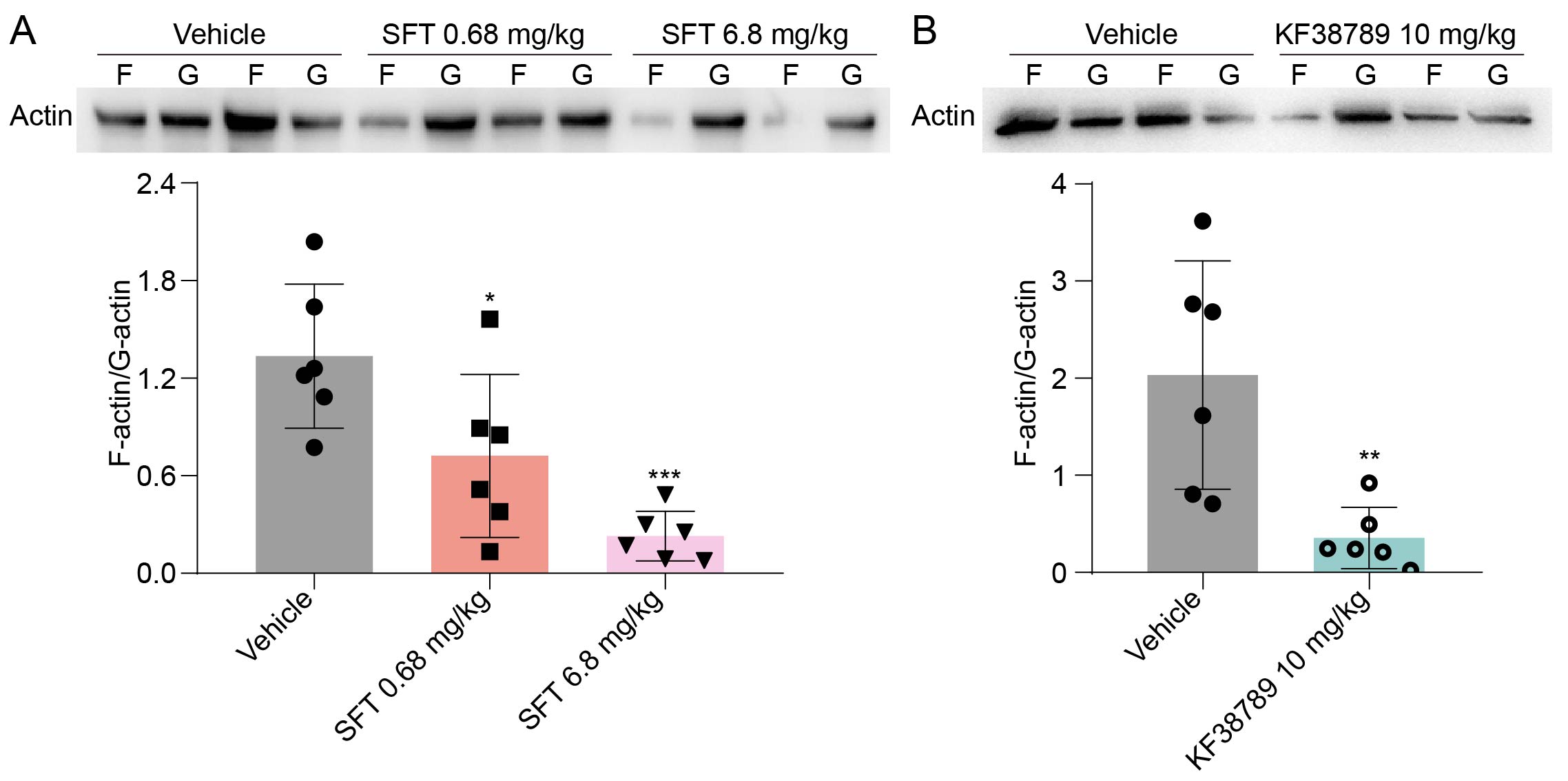
**

**Figure S20. Inhibiting P-selectin decreased F-/G-actin ratio *in vivo*.**

A-B. IB was used to investigate the alteration of F-/G-actin induced by SFT or KF38789 treatment in tumor tissues (*n* = 6, Tukey's multiple comparisons test or t test). SFT, sulfarotene; IB, immunoblotting. Data are presented as the mean ± SD; **p* < 0.05, ***p* < 0.01, ****p* < 0.001.


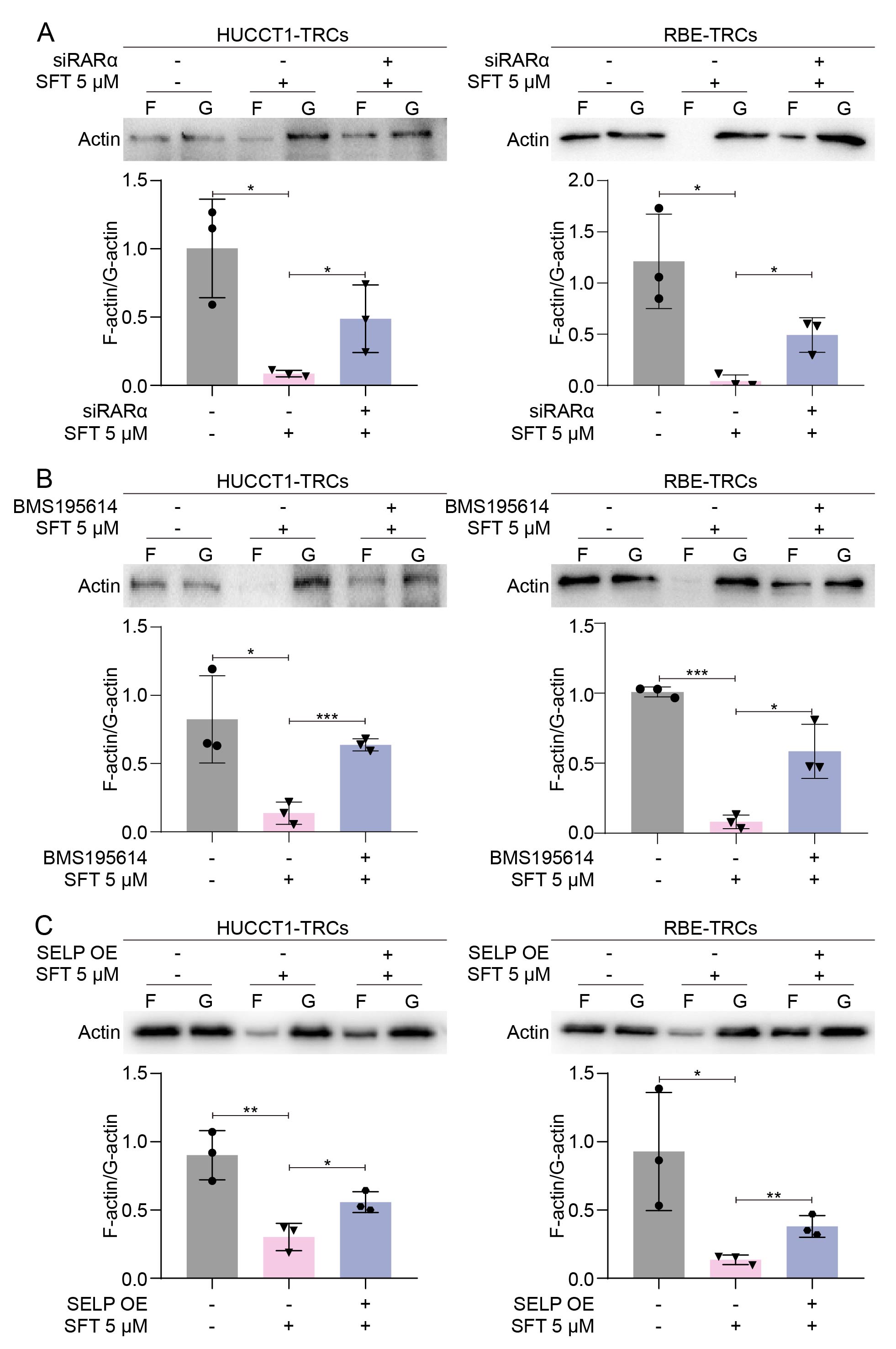


**Figure S21. SFT decreased F-/G-actin ratio *via* RARɑ/P-selectin axis *in vitro*.**

A & B. ICC-TRCs were treated with SFT (5 μM) for 48 h with or without RARɑ silence (A) or inhibition (B), and than IB was used to investigate the alteration of F-/G-actin ratio (*n* = 3, Tukey's multiple comparisons test). C. ICC-TRCs were treated with SFT (5 μM) for 48 h with or without *SELP* over-expression, and than IB was used to investigate the alteration of F-/G-actin ratio (*n* = 3, Tukey's multiple comparisons test). SFT, sulfarotene; ICC-TRCs, tumor-repopulating cells of intrahepatic cholangiocarcinoma; IB, immunoblotting. Data are presented as the mean ± SD; **p* < 0.05, ***p* < 0.01, ****p* < 0.001.


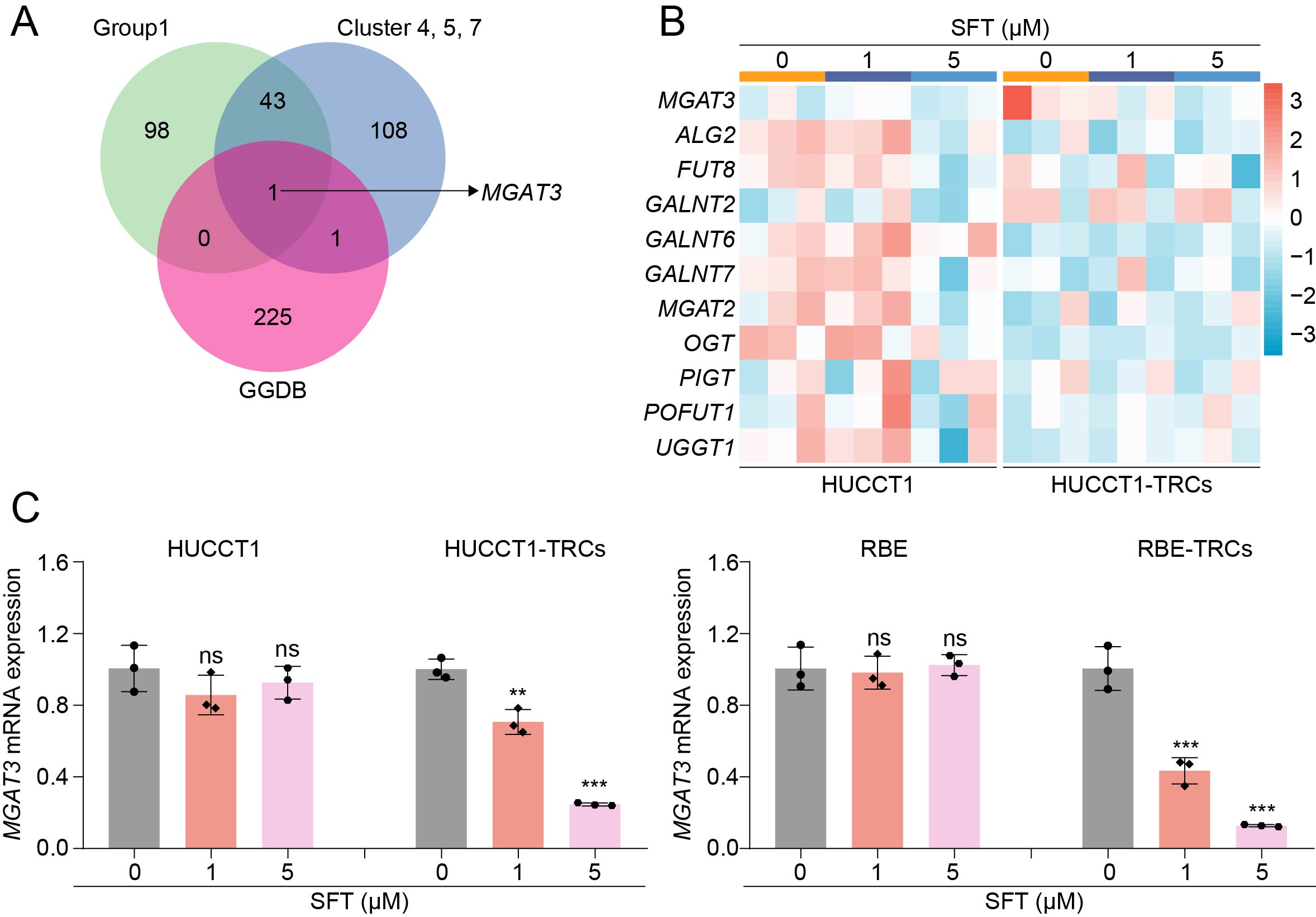


**Figure S22. The effect of SFT on mRNA of glycosyltransferase or glycosidases.**

A. Venn plot showed the overlapped genes among group 1, cluster 4,5,7 and genes from GGDB. B. Heatmap plot showed the change of candidate glycosyltransferase or glycosidases induced by SFT treatment in HUCCT1 and HUCCT1-TRCs. C. HUCCT1, HUCCT1-TRCs, RBE, RBE-TRCs were treated with SFT for 48 h, and MGAT3 mRNA was detected using qRT-PCR (*n* = 3, Tukey's multiple comparisons test). SFT, sulfarotene; GGDB, GlycoGene Database; TRCs, tumor-repopulating cells. Data are presented as the mean ± SD; ***p* < 0.01, ****p* < 0.001. ns, not significant.


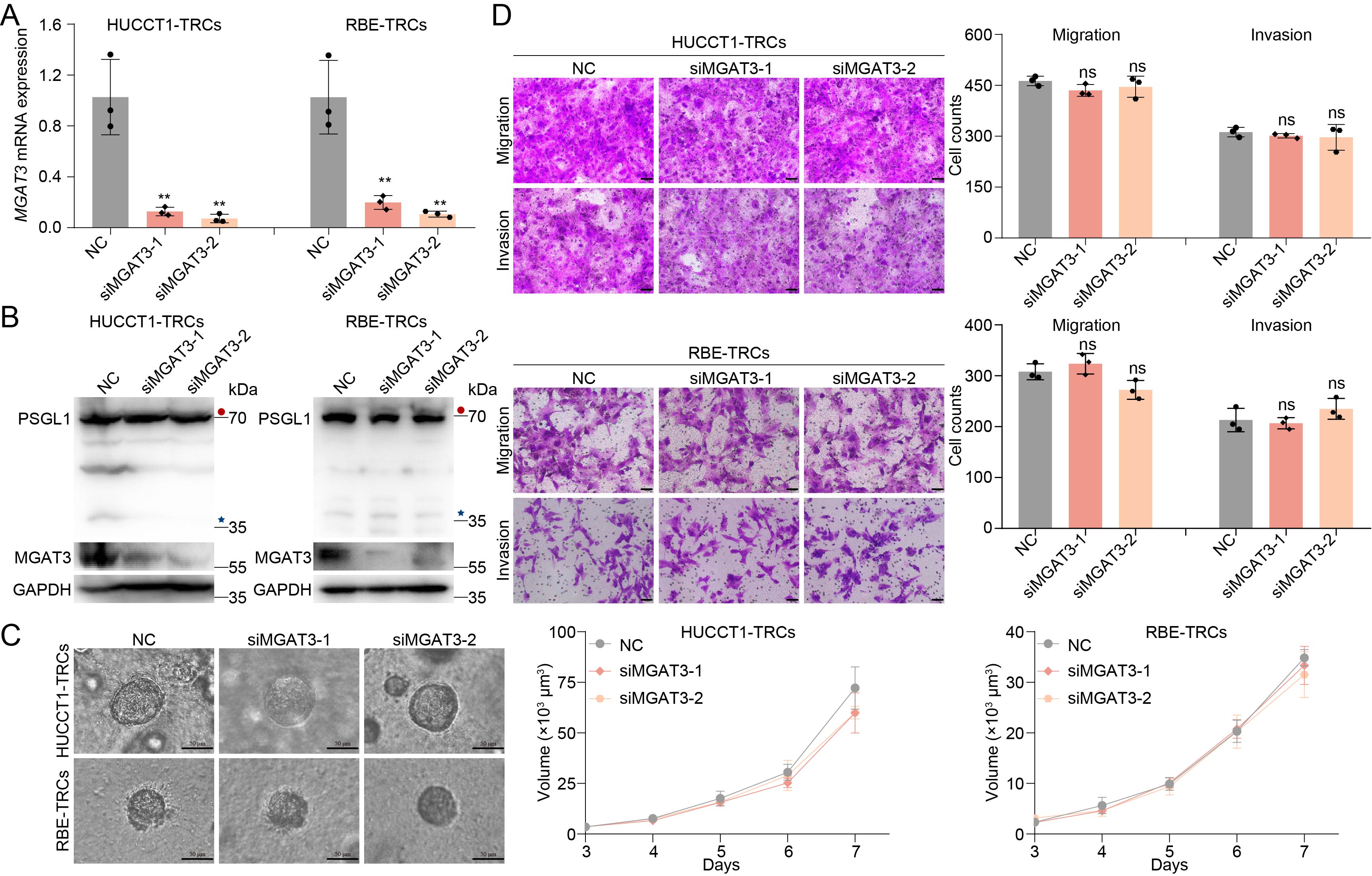


**Figure S23 Silencing MGAT3 failed to inhibit ICC-TRCs.**

A. The level of MGAT3 was detected using qRT-PCR after silenced by siRNA in ICC-TRCs (*n* = 3, t test). B. IB was employed to investigate the effects of MGAT3 silence on the glycosylation of PSGL1. C. Inhibition of silencing MGAT3 on colony spheroid growth in ICC-TRCs. Left panel, the representative images of ICC-TRCs colony spheroids on day 7. Right panel, quantitative analysis of time-dependent changes of colony spheroid sizes (*n* = 3, t test). D. Effects of silencing MGAT3 on the migration and invasion of ICC-TRCs. Left panel, the representative images. Right panel, quantitative analysis of Transwell assay calculated using Image J (*n* = 3, t test). ICC-TRCs, tumor-repopulating cells of intrahepatic cholangiocarcinoma; qRT-PCR, quantitative reverse transcription polymerase chain reaction; IB, immunoblotting. Data are presented as the mean ± SD; ***p* < 0.01. ns, not significant.


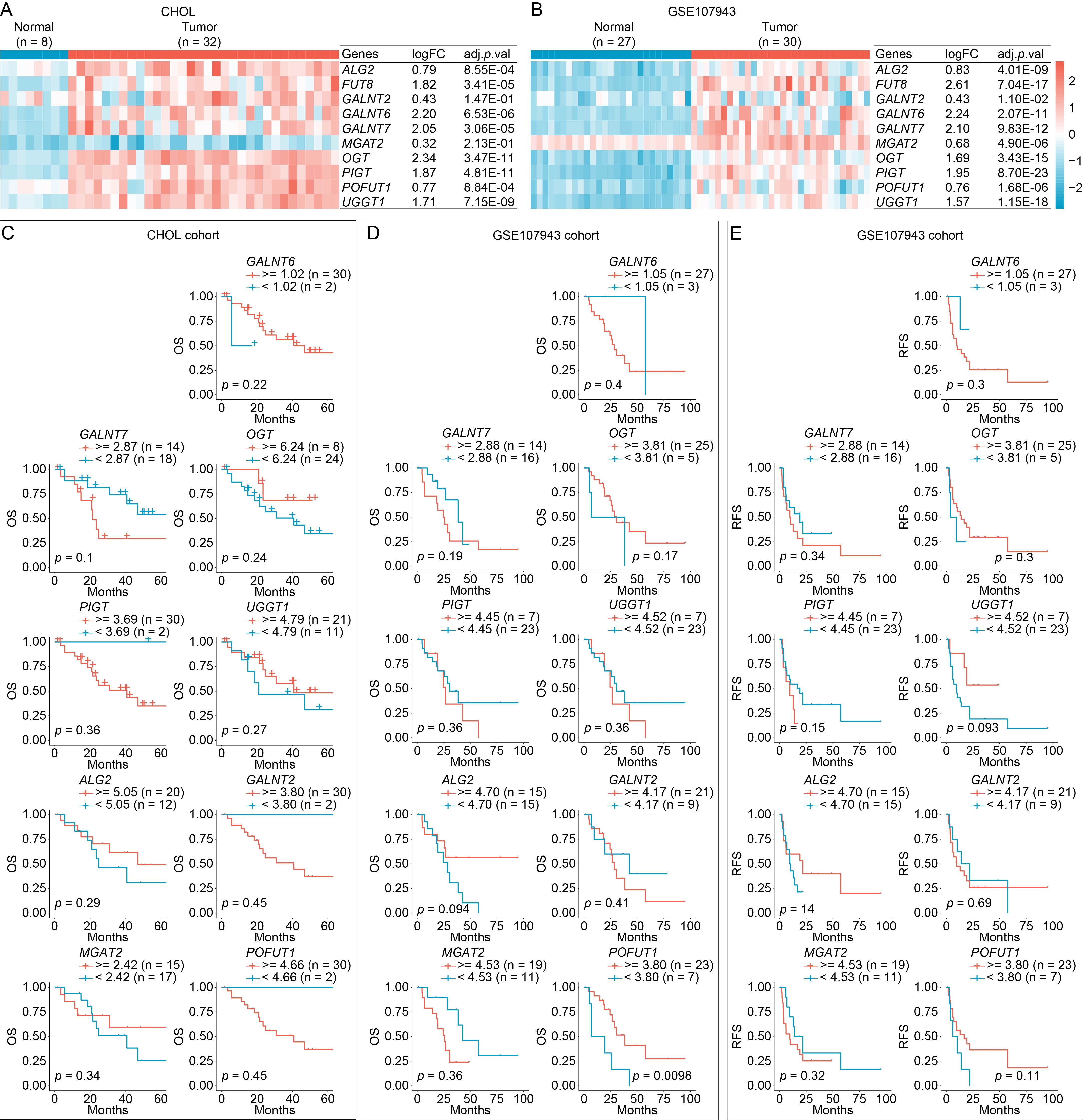


**Figure S24. The expression and survival analysis of candidate glycosyltransferase or glycosidases in ICC.**

A & B. Heatmap showed the expression of candidate glycosyltransferase or glycosidases between normal and tumor tissues in CHOL (A) and GSE107943 (B) cohort. C & D. Survival analysis of candidate glycosyltransferase or glycosidases in CHOL (C) and GSE107943 (D) cohort (Log-rank test). E. The relationship between RFS and candidate glycosyltransferase or glycosidases in GSE107943 cohort (Log-rank test). ICC, intrahepatic cholangiocarcinoma; OS, overall survival; RFS, relapse-free survival.


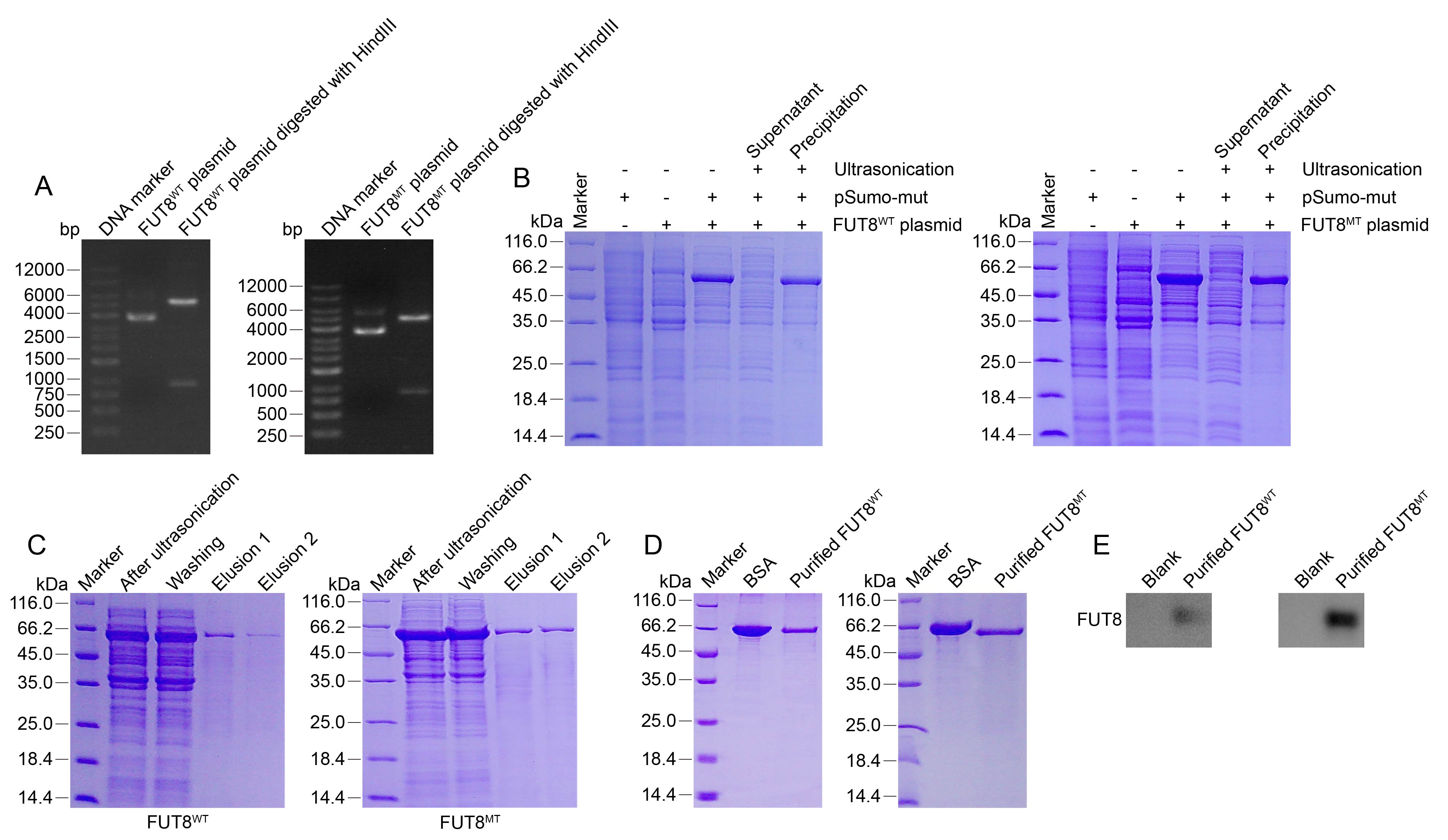


**Figure S25. Identification of the purified FUT8^WT^ and FUT8^MT^ protein.**

A. Restriction digestion map. B. FUT8^WT^ and FUT8^MT^ expression identification by SDS-PAGE analysis. C. SDS-PAGE analysis of FUT8^WT^ and FUT8^MT^ purification. D. Quality control of FUT8^WT^ and FUT8^MT^ by SDS-PAGE analysis. E. Quality control of FUT8^WT^ and FUT8^MT^ by IB. IB, immunoblotting.


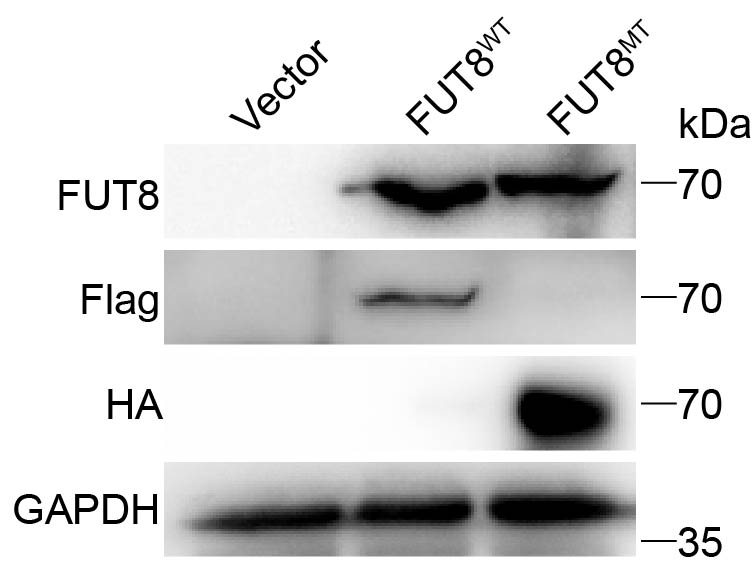


**Figure S26. Validation of the over-expression of FUT8.**

FUT8^WT^ and FUT8^MT^ plasmid were transfected into FUT8KO HUCCT1-TRCs, and IB was used to detect the over-expression effect. TRCs, tumor-repopulating cells; IB, immunoblotting.


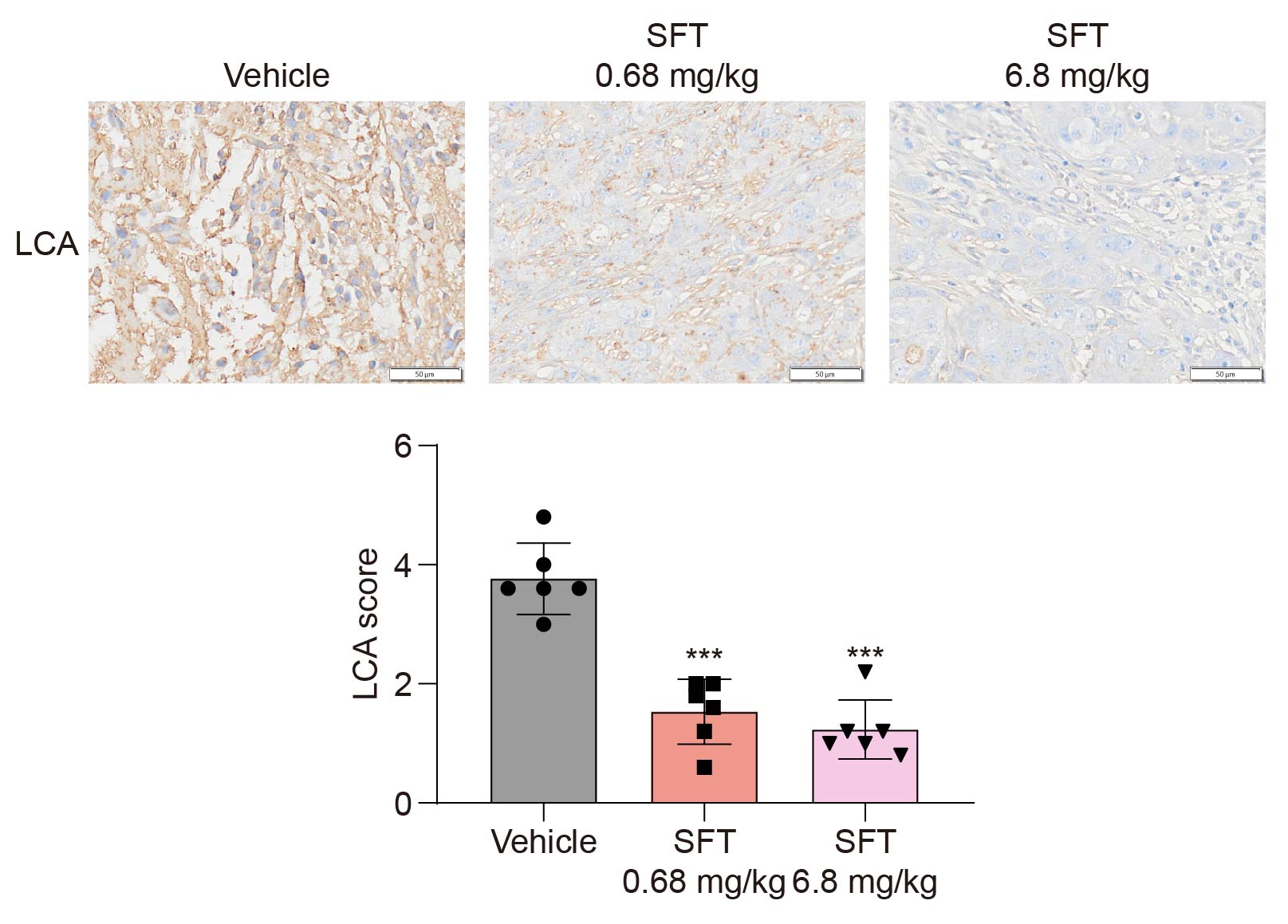


**Figure S27. SFT decreased the level of LCA *in vivo*.**

LCA staining of tumor tissues (*n* = 6, Tukey's multiple comparisons test). SFT, sulfarotene; LCA, Lens culinaris agglutinin. Data are presented as the mean ± SD; ****p* <

**Table S1 The IC_50_ of drugs in different cells.**

|  | **IC_50_ (μM)** | | | | | | | |
| --- | --- | --- | --- | --- | --- | --- | --- | --- |
| **Drugs** | **RBE** | **HUCCT1** | **HCCC9810** | **TFK1** | **AY-LTC2** | **RBE-TRCs** | **HUCCT1-TRCs** | **AY-LTC2-TRCs** |
| SFT | 3.83 ± 0.45 | 7.11 ± 1.45 | 9.08 ± 3.56 | 5.21 ± 0.57 | 3.24 ± 0.82 | 1.17 ± 0.44 | 1.53 ± 0.49 | 1.67 ± 0.19 |
| WYC-209A | 6.37 ± 0.51 | 5.27 ± 0.12 | / | / | 3.76 ± 0.84 | 2.22 ± 0.39 | 1.76 ± 0.11 | 1.17 ± 0.52 |
| WYC-209B | 6.39 ± 0.40 | 6.88 ± 0.53 | / | / | 3.82 ± 0.73 | 1.35 ± 0.64 | 1.51 ± 0.29 | 1.16 ± 0.23 |
| ATRA | > 100 | > 100 | > 100 | > 100 | / | > 100 | > 100 | / |
| 5-FU | 40.49 ± 4.52 | 8.05 ± 2.31 | 24.16 ± 6.24 | 8.05 ± 2.31 | / | 49.75 ± 4.14 | 12.47 ± 2.46 | / |
| CDDP | 9.39 ± 0.61 | 29.01 ± 1.95 | 4.23 ± 1.04 | 15.88 ± 1.79 | / | 29.11 ± 0.58 | 41.31 ± 4.68 | / |
| GEM | 0.05 ± 0.01 | 0.38 ± 0.16 | 0.17 ± 0.10 | 0.02 ± 0.01 | / | 1.06 ± 0.32 | 3.10 ± 0.49 | / |
| PEM | 7.95 ± 1.84 | 14.84 ± 2.40 | 61.66 ± 9.05 | 2.71 ± 0.62 | / | 10.13 ± 1.04 | 13.55 ± 3.16 | / |

Table S2 The results of Caco-2 permeability assay.

| **Compound** | **Mean Papp (cm/s)** | | **Efflux ratio** | **Mean recovery (%)** | | **Rank** | |
| --- | --- | --- | --- | --- | --- | --- | --- |
|  | **A to B** | **B to A** |  | **A to B** | **B to A** | **Absorption rate** | **Efflux transport substrate** |
| **SFT** | 0.63 | 0.19 | 0.3 | 0.77 | 77.86 | Moderate | Poor or none |

Papp, apparent permeability coefficient.

**Table S3 The metabolites of SFT *in vitro* and *in vivo***

| **ID** | **Metabolic change** | **Relative abundance (UV peak area%)** | | | | | | | | | | | |
| --- | --- | --- | --- | --- | --- | --- | --- | --- | --- | --- | --- | --- | --- |
|  |  | **Liver microsome (*In vitro*)** | | | | | **Liver cell (*In vitro*)** | | | | | **Rat (*In vivo*)** | |
|  |  | **Human** | **Monkey** | **Dog** | **Rat** | **Mouse** | **Human** | **Monkey** | **Dog** | **Rat** | **Mouse** | **PO** | **IV** |
| M1 | Hydrolysis, S-cysteine binding, and hydrogenation |  |  |  |  |  | 1.36 | + | + | + | + |  |  |
| M2 | Hydrolysis, S-cysteine binding, and hydrogenation |  |  |  |  |  | 1.61 | 0.37 | + | + | + |  |  |
| M3 | Hydrolysis, S-glutathione binding, and hydrolysis of glutamic acid and hydrogenation |  |  |  |  |  | 1.46 | + | + | + | ND |  |  |
| M4 | Hydrolysis, S-glutathione binding, and hydrogenation |  |  |  |  |  | 0.66 | 1.07 | 1.11 | 3.36 | 5.7 |  |  |
| M4a | Hydrolysis, S-glutathione binding, and hydrolysis of glutamic acid and hydrogenation |  |  |  |  |  |  |  |  |  |  | + | + |
| M5 | Hydrolysis, S-glutathione binding, and hydrogenation | 0.11 | 0.16 | 0.21 | 0.45 | 2.04 | 1.8 | 2.5 | 1.07 | **30.38** | **14.49** | + | + |
| M6 | Double oxidation, S-cysteine binding, and hydrogenation |  |  |  |  |  | + | 2.92 | ND | ND | + |  |  |
| M7 | Monooxidation, S-glutathione binding, and hydrogenation |  |  |  |  |  | + | 5.91 | ND | + | + |  |  |
| M8 | S-glutathione binding, hydrolysis, deglutamination, and hydrogenation | 0.17 | 0.46 | 0.18 | ND | ND | **28.2** | **11.83** | 2.02 | + | ND | + | + |
| M9 | S-glutathione binding, hydrolysis, deglutamination, and hydrogenation | 0.05 | 0.18 | 0.04 | ND | ND | **17.82** | 2.93 | + | + | ND |  |  |
| M10 | Hydrolysis and double oxidation | 8.63 | **23.05** | 1.08 | 0.5 | 0.1 | + | 0.63 | + | ND | 1.71 |  |  |
| M11 | S-glutathione binding and hydrogenation | + | 0.19 | 0.16 | ND | ND | 5.04 | **10.41** | **17.19** | 7.18 | 7.94 | 5.14 | 4.37 |
| M12 | S-glutathione binding and hydrogenation | + | 0.21 | 0.43 | 0.15 | 0.13 | **12.89** | **25.87** | 6.72 | **22.08** | **27.8** |  |  |
| M13 | Hydrolysis and double oxidation | 0.42 | 1.1 | + | ND | ND |  |  |  |  |  |  |  |
| M14 | Monooxidation, S-glutathione binding, hydrolysis, deglutamination, and hydrogenation |  |  |  |  |  | 1.69 | 0.77 | + | ND | ND |  |  |
| **M15** | **Hydrolysis** | **78.49** | **61.83** | **45.31** | **95.6** | **96.58** | **12.47** | 7.58 | **37.07** | **11.41** | **31.04** | **20.59** | **23.18** |
| M16 | Monooxidation, S-glutathione binding and hydrogenation |  |  |  |  |  | 2.35 | 4.93 | 2.29 | 2.65 | 1.77 |  |  |
| M17 | S-glutathione binding and hydrogenation | 0.07 | 0.25 | 0.38 | 0.06 | 0.03 | 4.24 | 3.75 | 3.12 | 7.55 | 1.57 | **55.82** | **72.45** |
| M17a | Hydrolysis and hydrogenation |  |  |  |  |  |  |  |  |  |  | + | + |
| M18 | S-glutathione binding and hydrogenation | 0.19 | 0.45 | 0.6 | 0.27 | 0.09 | 5.91 | 7.59 | 1.06 | 8.23 | 2.7 |  |  |
| M19 | Hydrolysis and monooxidation | **10.05** | 4.92 | **15.71** | 2.92 | 0.97 |  |  |  |  |  |  |  |
| M20 | Hydrolysis, reduction, S-glutathione binding, and hydrogenation |  |  |  |  |  | 0.49 | 1.39 | 3.7 | 3.12 | 2.61 |  |  |
| M21 | Demethylation | 0.29 | ND | 1.09 | ND | ND |  |  |  |  |  |  |  |
| M22 | Reduction, S-glutathione binding and hydrogenation |  |  |  |  |  | + | 2.81 | 8.39 | 0.67 | 0.93 |  |  |
| M23 | Trioxidation | 0.11 | 6.66 | ND | ND | ND |  |  |  |  |  |  |  |
| M24 | Reduction, S-glutathione binding and hydrogenation |  |  |  |  |  | + | 5.3 | **12.41** | 0.78 | 0.75 |  |  |
| M25 | Double oxidation | 0.79 | 0.26 | 3.59 | ND | ND |  |  |  |  |  |  |  |
| SFT |  | 0.62 | 0.27 | **24.2** | 0.07 | 0.06 | 0.31 | 0.26 | 3.44 | 0.19 | 0.22 | + | + |
| M26 | Monooxidation and hydrogenation |  |  |  |  |  | 1.26 | 0.23 | 0.21 | + | + | + | + |
| M27 | Monooxidation | ND | ND | 7.01 | ND | ND |  |  |  |  |  |  |  |
| M28* | Sulfoxide reduction and hydrolysis |  |  |  |  |  |  |  |  |  |  | 7.83 | + |
| M29* | Sulfoxide reduction, hydrolysis and hydrogenation |  |  |  |  |  |  |  |  |  |  | **10.63** | + |
| Summary |  | 100 | 100 | 100 | 100 | 100 | 100 | 100 | 100 | 100 | 100 | 100 | 100 |

+, the UV peak signal at 254-360 nm wavelength is too weak to be integrated;

ND, not detected;

*, M28 and M29 cannot be separated, and the UV peak area is calculated from the ratio of mass spectrometry peak areas.

Table S4 The toxicity assess of SFT in rats.

| **Treatment** | **Survival rate** | **Symptoms** | **Weight change** | **Food intake** |
| --- | --- | --- | --- | --- |
| Vehicle, po |  |  |  |  |
| Male (n = 2) | 100% | Normal | Normal | Normal |
| Female (n = 2) | 100% | Normal | Normal | Normal |
| SFT 1000 mg/kg, po |  |  |  |  |
| Male (n = 3) | 100% | Normal | 2.89% reduction | Normal |
| Female (n = 3) | 100% | Normal | 3.38% reduction | Normal |
| SFT 1500 mg/kg, po |  |  |  |  |
| Male (n = 3) | 100% | Normal | 8.83% reduction | Normal |
| Female (n = 3) | 100% | Normal | 4.69% reduction | Normal |
| SFT 2000 mg/kg, po |  |  |  |  |
| Male (n = 3) | 100% | Emaciation (33%), Diarrhea (33%) | 11.96% reduction | Decreased |
| Female (n = 3) | 100% | Diarrhea (100%) | 6.49% reduction | Normal |
| Vehicle, iv |  |  |  |  |
| Male (n = 2) | 100% | Normal | Normal | Normal |
| Female (n = 2) | 100% | Normal | Normal | Normal |
| SFT 10 mg/kg, iv |  |  |  |  |
| Male (n = 3) | 100% | Normal | Normal | Normal |
| Female (n = 3) | 100% | Normal | Normal | Normal |
| SFT 20 mg/kg, iv |  |  |  |  |
| Male (n = 3) | 100% | Decreased activity within 15 min after iv, and recovered after 1-3 h. | Normal | Normal |
| Female (n = 3) | 100% |  | Normal | Normal |

**Table S5 The possible binding site between glycosyltransferase and sulfarotene identified by Lip-SMap.**

| **ID** | **Proteins** | **Sequence** | **Start** | **End** | **logFC** | ***p* value** |
| --- | --- | --- | --- | --- | --- | --- |
| O15294 | OGT1_HUMAN | AFLDSLPDVK | 743 | 752 | -25.0 | 7.78E-3 |
| O15294 | OGT1_HUMAN | GSVAEAEDCYNTALR | 307 | 321 | -23.0 | 1.62E-12 |
| O15294 | OGT1_HUMAN | LCPTHADSLNNLANIK | 322 | 337 | -23.3 | 2.20E-12 |
| O15294 | OGT1_HUMAN | LCPTHADSLNNLANIKR | 322 | 338 | -23.6 | 2.48E-13 |
| O15294 | OGT1_HUMAN | QIVCDWTDYDER | 474 | 485 | -21.5 | 5.26E-3 |
| O15294 | OGT1_HUMAN | VAASQLTCLGCLELIAK | 955 | 971 | -25.4 | 2.15E-14 |
| Q10469 | MGAT2_HUMAN | SLVYQLNFDQTLR | 93 | 105 | -22.9 | 4.42E-12 |
| Q10471 | GALT2_HUMAN | NKFNQVESDKLR | 102 | 113 | 26.7 | 2.10E-12 |
| Q10471 | GALT2_HUMAN | VLTFLDSHCECNEHWLEPLLER | 219 | 240 | -25.6 | 4.27E-12 |
| Q86SF2 | GALT7_HUMAN | LEGWQGNPPPIYVGSSPTLK | 444 | 463 | -22.9 | 1.88E-12 |
| Q8NCL4 | GALT6_HUMAN | NLGTNQCLDVGENNR | 503 | 517 | -22.4 | 1.63E-13 |
| Q8NCL4 | GALT6_HUMAN | SLGPDTRPPECVDQK | 155 | 169 | -24.4 | 3.33E-10 |
| Q969N2 | PIGT_HUMAN | GELSTLLYNTHPYR | 366 | 379 | -23.2 | 2.70E-10 |
| Q9BYC5 | FUT8_HUMAN | TLILESQNWR | 240 | 249 | -22.6 | 3.21E-13 |
| Q9H488 | OFUT1_HUMAN | DLQGRPSSFFGMDRPPK | 367 | 383 | -22.2 | 4.67E-14 |
| Q9H488 | OFUT1_HUMAN | STAAPLTMTMCLPDLK | 273 | 288 | -25.3 | 6.00E-3 |
| Q9H553 | ALG2_HUMAN | MVQQSDLGQYVTFLR | 287 | 301 | -23.1 | 2.83E-12 |
| Q9NYU2 | UGGG1_HUMAN | NYLSPTFK | 1293 | 1300 | 24.1 | 1.61E-14 |
| Q9NYU2 | UGGG1_HUMAN | TIDLCNNPMTK | 1489 | 1499 | -22.7 | 8.36E-3 |
| Q9NYU2 | UGGG1_HUMAN | VEEDVASDLVMK | 938 | 949 | -23.9 | 6.54E-3 |

Table S6 The source of antibodies.

| **Antibodies** | **Reference** | **Company** | **Application** |
| --- | --- | --- | --- |
| RARɑ | A19551 | ABclonal | IB, IHC |
| RARɑ | 10331-1-AP | Proteintech | CHIP, IF |
| RARβ | ab124701 | Abcam | IB |
| RARγ | ab187159 | Abcam | IB |
| GAPDH | AC033 | ABclonal | IB |
| H3 | A2348 | ABclonal | IB |
| P-selectin | A4989 | ABclonal | IB, IHC |
| PSGL1 | A1660 | ABclonal | IB |
| PSGL1 | ab227836 | Abcam | IP |
| FUT8 | 66118-1-Ig | Proteintech | IB |
| HA | AE008 | ABclonal | IB |
| FLAG | AE005 | ABclonal | IB |
| p-AKT^Ser473^ | T40067 | Abmart | IB |
| t-AKT | T55561 | Abmart | IB |
| MGAT3 | A24347 | ABclonal | IB |
| Ki67 | A20018 | ABclonal | IF, IHC |
| Cleaved-caspase3 | #9664 | Cell Signaling Technology | IF, IHC |
| HRP-labeled Goat Anti-Rabbit IgG (H+L) | A0208 | Beyotime | IB |
| HRP-labeled Goat Anti-Mouse IgG(H+L) | A0216 | Beyotime | IB |
| HRP-conjugated Mouse Anti-Rabbit IgG Light Chain | AS061 | ABclonal | IP |
| Cy3-labeled Goat Anti-Rabbit IgG (H+L) | A0516 | Beyotime | IF |
| FITC-labeled Goat Anti-Rabbit IgG (H+L) | A0562 | Beyotime | IF |

Table S7 The sequence of primers.

| **Genes** | **Sequence** |
| --- | --- |
| *EpCAM* | F - ‘CGCAGCTCAGGAAGAATGTGT’ |
|  | R - ‘GCTCTCATCGCAGTCAGGAT’ |
| *SOX2* | F - ‘CCTACAGCATGTCCTACTCGCA’ |
|  | R - ‘CTGGAGTGGGAGGAAGAGGTAAC’ |
| *CD90* | F - ‘CCATCATAACCCGATCCAGG’ |
|  | R - ‘GGGTGAACTGCTGGTATTCT’ |
| *LGR5* | F - ‘ACCTGAACTAAGAACACTGACTCT’ |
|  | R - ‘CTGATTGCAGACGGTTTGAGG’ |
| *CD117* | F - ‘GAGATGTGACTCCCGCCATC’ |
|  | R - ‘TCACAGGTAGTCGAGCGTTTC’ |
| *RARA* | F - ‘CTACCCCGCATCTACAAGCC’ |
|  | R - ‘GGTCGTTTCTCACAGACTCCTT’ |
| *RARB* | F - ‘TAATCTGTGGAGACCGCCAG’ |
|  | R - ‘ACACGCTCTGCACCTTTAGC’ |
| *RARG* | F - ‘CTGTGCGAAATGACCGGAAC’ |
|  | R - ‘CTGCACTGGAGTTCGTGGTA’ |
| *SELP* | F - ‘AAGCCCTGACTTACTTTGGTGG’ |
|  | R - ‘ATATGTTCCTAGGTGGCTGTGAGG’ |
| *SELP* prime A | F - ‘ACAAATAGTGAGGATTTGCTCTGT’ |
|  | R - ‘GAGAGTAGGCACACAGTGGT’ |
| *SELP* prime B | F - ‘GCACGTTGGAATTTGGGACC’ |
|  | R - ‘GGGTCTGGACTCTGAACTGC’ |
| *SELPLG* | F-‘CTCTGTTACTCACAAGGGCATT’ |
|  | R-‘CCAGCGCCAAGATTAGGATGG’ |
| *MGAT3* | F-‘CCCACTCTACTCCCACTCG’ |
|  | R-‘CGCACGAAATACTCGGTGGT’ |
| *GAPDH* | F - ‘ATCATCAGCAATGCCTCCTG’ |
|  | R- ‘ATGGACTGTGGTCATGAGTC’ |
| *Cas9 (hSpCsn1)* | F-‘CTACGACGACGACCTGGACAA’ |
|  | R-‘GCCTTGGTGATCTCGGTGTTC’ |

Table S8 The targeted sequence of siRNA.

| **siRNA** | **Targeted sequence** |
| --- | --- |
| siRARɑ | ATTACTGACCTGCGAAGCA |
| siSELP-1 | CTGCAACGCTGGATTCACA |
| siSELP-2 | CCTGAAGATTCCTGAACGA |
| siMGAT3-1 | GTGTGCGAGTCCAACTTCA |
| siMGAT3-2 | GCCACAAGGTGCTCTATGT |
